# Describing Biological Vulnerability in Small, Vulnerable Newborns in Urban Burkina Faso (DenBalo): Gut Microbiota, Immune System, and Breastmilk Assembly

**DOI:** 10.3390/nu16234242

**Published:** 2024-12-09

**Authors:** Lionel Olivier Ouédraogo, Lishi Deng, Cheick Ahmed Ouattara, Anderson Compaoré, Moctar Ouédraogo, Alemayehu Argaw, Carl Lachat, Eric R. Houpt, Queen Saidi, Filomeen Haerynck, Justin Sonnenburg, Meghan B. Azad, Simon J. Tavernier, Yuri Bastos-Moreira, Laeticia Celine Toe, Trenton Dailey-Chwalibóg

**Affiliations:** 1 Department of Food Technology, Safety and Health, Faculty of Bioscience Engineering, Ghent University, 9000 Ghent, Belgium; lionel.olivier.ouedraogo@ugent.be (L.O.O.); lishi.deng@ugent.be (L.D.); alemayehuargaw.alemayehu@ugent.be (A.A.); carl.lachat@ugent.be (C.L.); yuri.bastosmoreira@ugent.be (Y.B.-M.); laeticiaceline.toe@ugent.be (L.C.T.); 2 Centre Muraz, Bobo-Dioulasso 01 BP 390, Burkina Faso; 3 Agence de Formation de Recherche et d’Expertise en Santé pour l’Afrique (AFRICSanté), Bobo-Dioulasso 01 BP 298, Burkina Faso; ouattaracheickahmed@gmail.com (C.A.O.); discompa4523@gmail.com (A.C.); obmoctar@gmail.com (M.O.); 4 Division of Infectious Diseases and International Health, Department of Medicine, University of Virginia, Charlottesville, VA 22903, USA; erh6k@uvahealth.org (E.R.H.); kez5rk@uvahealth.org (Q.S.); 5 Primary Immunodeficiency Research Lab (PIRL) at Ghent University Hospital (UZGent), 9000 Ghent, Belgium; filomeen.haerynck@uzgent.be (F.H.); simon.tavernier@irc.vib-ugent.be (S.J.T.); 6 Department of Microbiology and Immunology and Center for Human Microbiome Studies, Stanford University, Stanford, CA 94305, USA; jsonnenburg@stanford.edu; 7 Department of Pediatrics and Child Health, University of Manitoba, Winnipeg, MB R3T 2N2, Canada; meghan.azad@umanitoba.ca; 8 Manitoba Interdisciplinary Lactation Center (MILC), Children’s Hospital Research Institute of Manitoba, Winnipeg, MB R3E 3P4, Canada; 9 Center for Primary Immunodeficiency, Ghent University Hospital, 9000 Ghent, Belgium; 10 Jeffrey Modell Diagnosis and Research Center, Ghent University Hospital, 9000 Ghent, Belgium; 11 Center for Medical Genetics, Ghent University Hospital, 9000 Ghent, Belgium; 12 Department of Biomedical Molecular Biology, Ghent University, 9000 Ghent, Belgium; 13 Unit of Molecular Signal Transduction in Inflammation, VIB-UGent Center for Inflammation Research, 9052 Ghent, Belgium; 14 Center of Excellence in Mycotoxicology and Public Health, MYTOX-SOUTH® Coordination Unit, Faculty of Pharmaceutical Sciences, Ghent University, 9000 Ghent, Belgium; 15 Unité Nutrition et Maladies Métaboliques, Institut de Recherche en Sciences de la Santé (IRSS), Bobo-Dioulasso 01 BP 545, Burkina Faso

**Keywords:** small vulnerable newborns, preterm birth, small for gestational age, low birth weight, immune system, metagenomics, metabolomics, proteomics, DenBalo

## Abstract

*Background*: Small vulnerable newborns (SVNs), including those born preterm, small for gestational age, or with low birth weight, are at higher risk of neonatal mortality and long-term health complications. Early exposure to maternal vaginal microbiota and breastfeeding plays a critical role in the development of the neonatal microbiota and immune system, especially in low-resource settings like Burkina Faso, where neonatal mortality rates remain high. *Objectives*: The DenBalo study aims to investigate the role of maternal and neonatal factors, such as vaginal and gut microbiota, immune development, and early nutrition, in shaping health outcomes in SVNs and healthy infants. *Methods*: This prospective cohort observational study will recruit 141 mother-infant pairs (70 SVNs and 71 healthy controls) from four health centers in Bobo-Dioulasso, Burkina Faso. The mother-infant pairs will be followed for six months with anthropometric measurements and biospecimen collections, including blood, breast milk, saliva, stool, vaginal swabs, and placental biopsies. Multi-omics approaches, encompassing metagenomics, metabolomics, proteomics, and immune profiling, will be used to assess vaginal and gut microbiota composition and functionality, immune cell maturation, and cytokine levels at critical developmental stages. *Conclusions*: This study will generate comprehensive data on how microbiota, metabolomic, and proteomic profiles, along with immune system development, differ between SVNs and healthy infants. These findings will guide targeted interventions to improve neonatal health outcomes and reduce mortality, particularly in vulnerable populations.

## 1. Introduction

Preterm birth, low birth weight (LBW), and small-for-gestational-age (SGA) newborns, collectively referred to as small vulnerable newborns (SVNs), account for the majority of neonatal mortality and are at higher risk for adverse health outcomes [1]. Understanding the biological processes that influence their growth and survival is crucial for designing targeted interventions to improve neonatal and their long-term health outcomes, particularly in Burkina, where the neonatal mortality rate remains alarmingly high at 24.6 per 1000 live births in 2022 [2], with an intra-hospital mortality rate as high as 27.8% in the Hauts-Bassins region [3]. In this context, SVNs contribute to these mortality statistics, with LBW prevalence estimated at 8–11% [4,5], preterm birth rates at 5.4–5.7% [6], and SGA infants accounting for 27.9% of births [7].

The neonatal period, encompassing the first days and weeks of life, is a critical window for the establishment of key biological systems essential for growth, development, and adaptation to life outside the womb [8]. These systems include the gut microbiota and immune system, which play a crucial role in shaping long-term health outcomes.

Vaginal delivery is a key event that exposes neonates to maternal vaginal and intestinal microbiota, providing the first gut microbial inoculum [9,10,11,12,13]. These early colonizers are linked to changes in the gut’s protein composition and the metabolic environment, suggesting they play a foundational role in shaping the developing microbiome [14]. However, whether the assembly and maturation of gut microbiota differ based on delivery term and birthweight, and how this impacts infant growth and development, remains unclear.

Neonatal nutrition is another significant critical factor that influences both gut microbiota and immune function. Breastfeeding is a primary determinant of infant gut colonization after birth [15], initiating tropic priming of the newborn gut and providing essential immunological components [16]. Despite its importance, the interplay between neonatal microbiome and immune system development, particularly in the context of nutrition, has not been extensively studied. This gap is even more pronounced in SVNs, who are predisposed to health challenges such as gut dysbiosis caused by physiological immaturity and prenatal/postnatal factors (e.g., prenatal maternal illness, rapid delivery, antibiotic provision) [17] and immature immune systems with compromised innate and adaptive immunity, exacerbated by complications related to preterm birth [18]. However, studies showed that the colostrum from mothers of SGA neonates is compositionally similar to that of full-term neonates [19]. In contrast, colostrum from mothers of preterm newborns, who are often characterized as LBW due to prematurity [20], exhibits a distinct composition. It contains higher levels of protein, fat, free amino acids, sodium, and bioactive milk components, such as human milk oligosaccharides (HMOs), cytokines, and lactoferrin [21,22]. Despite these differences, the association between early milk composition and infant growth and development remains inadequately explored. Consequently, it is unclear which specific components are essential for fostering a healthy gut microbiota and supporting a robust immune system in infants [17,18,19,20,21,22].

The neonatal period is also marked by heightened vulnerability to infectious diseases, with neonatal infections contributing to 40% of mortality in children under five [23]. Early microbial colonization plays a vital role in shaping the immune system and providing protection against infections [24]. Studies have shown that the neonatal immune system undergoes rapid development in the first week of life, with long-term health implications [8,25,26]. However, the effects of SVN status on immune system development and the role of human milk and the gut microbiome in these processes remain poorly understood [18,27].

Major advances in systems biology have facilitated unbiased and integrated analyses of high-dimensional omics databases, equipping researchers with essential bioinformatic tools to explore the neonatal microbiome and immunome [8]. This progress paves the way for a transformative shift toward impactful research that examines the interplay between these biological systems and newborn nutrition, addressing critical gaps in our understanding of neonatal health.

The DenBalo study aims to investigate the role of maternal and neonatal factors, such as vaginal and gut microbiota, immune development, and the composition of human milk, in shaping health outcomes in SVNs and healthy infants in urban Burkina Faso. To achieve this, the project will pursue three main objectives:

1. To independently describe and compare the gut microbiota, immune system, and breastmilk (collectively referred to as “bio-networks” in this protocol) in SVNs and healthy controls, the latter comprising full-term neonates of normal birth weight;
2. To detail the assembly and development of these bio-networks at high resolution during the first days and weeks of life;
3. To integrate these bio-network data using multi-omics systems biology approaches for comprehensive analysis.

## 2. Materials and Methods

### 2.1. Study Setting and Participants

The DenBalo study follows a multicenter, prospective cohort design conducted at three health and social promotion centers (CSPSand one medical center (i.e., four health centers) with a surgical unit (CMA) in the Dô Health District, Bobo-Dioulasso, Burkina Faso. These health centers were selected based on data from a previous study conducted by our research group on postnatal weight loss in newborns, which allowed for real-time assessment of birth rate and evaluated the CSPS staff’s capacity to manage research demands.

**Figure 1:**
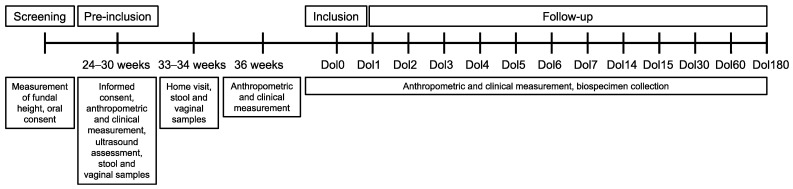
Flow chart of the DenBalo study schedule.

Initial sample size calculations were based on data from the health centers. A sample of 140 mother-infant dyads (70 SVNs and 70 controls) was targeted. These participants will be followed up until the infants reach six months of age. The recruitment process and follow-up schedule are outlined in Figure 1 and further described below.

### 2.2. Recuitment Procedure

The selection and inclusion of participants will proceed in three steps:

***Step 1. Screening***

During routine antenatal consultations (ANC), women aged 15 to 45 years in their early third trimester, with a symphysial fundal height between 24 and 27 cm, will be screened for eligibility (see Table 1). Project midwives will explain the study objectives, obtain oral consent, and refer potential eligible women for an ultrasound at the CMA of Dô to confirm gestational age (GA).

***Step 2. Pre-inclusion***

In the third trimester, traditional fetal biometric parameters such as abdominal circumference, biparietal diameter, and head circumference become less accurate for estimating GA [1]. Instead, the combination of transcerebellar diameter (TCD and femur length (FL), with an accuracy of ±15.1 days compared to the gold standard, will be used to estimate GA [28].

Following ultrasound assessments, FL and TCD will be measured in duplicate (i.e., on different images) to estimate GA. Any difference between repeated measurements of more than ±1 week of GA (i.e., >1.8 mm for TCD or >0.24 mm for FL) will result in a mandatory third measurement on a new image [29]. The quality control criteria for TCD and FL assessments are shown in Table 2.

Gynecologists will visually assess the quality of their own images according to predefined criteria and rank them in descending order, from best to worst [i.e., 1st, 2nd, (and possibly 3rd)]. GA will be calculated using the Alliance for Maternal Newborn Health Improvement (AMANHI) formula based on two best measurements [29].

$$GA_{AMANHI}=e^{[\left(0.3825021\times \ln\left(TCD\right)+0.3321277\times \ln\left(FL\right)\right]}$$

Singleton pregnancies with GA between 24 and 29 weeks (6 days) will be eligible for pre-inclusion (Table 1). After pre-inclusion, at 33 to 3 weeks of gestation, a team of sociologists will visit the participant’s family/family-in-law to explain the high-frequency nature of the study and facilitate data collection. The study’s aims, methods, procedures, risks, and limitations will be thoroughly explained to the participants. Informed consent will be obtained, and a highly visible “green card” will be attached to the participant’s health booklet to facilitate identification at the CSPS.

***Step 3: Inclusion***

At 36 weeks of gestation, study staff will contact pre-included participants by phone to encourage them to present at the CSPS with their green card when labor begins. Participants should show their “green card” upon arrival for direct referral to the project midwife. The midwife will assess for membranes, as intact membranes are required for vaginal microbiota sampling. If the membranes are intact, newborns will be categorized into either the SVN group or the control group based on inclusion criteria (Table 1). Sequential recruitment will be followed, starting with the first SVN enrolled. A matching control neonate will then be recruited for each SVN, with the process continuing until the target sample size is achieved over a 12-month recruitment period.

The inclusion and exclusion criteria by stage are presented in Table 1.

### 2.3. Data Collection

Table 3 shows the overview of the time schedule and measurements of this study. Anthropometric, clinical measures and a series of biospecimen samples will be collected from women and their infants at multiple time points covering pregnancy and lactation, including 24–30 weeks, 33–34 weeks, and 36 weeks at birth, day of life (Dol) 1, 2, 3, 4, 5, 6, 7, 14, 15, 30, 60, and 180.

#### 2.3.1. Metadata

***Anthropometric and clinical measurements***

During pre-inclusion and at each visit after inclusion, anthropometric measurements from all women will be taken, and infant’s anthropometric measurements will be taken at each visit after birth. Maternal weight will be measured to the nearest 100 g with a Seca 876 scale, height to the nearest 1 cm with a ShorrBoard Infant/Child/Adult, and MUAC will be measured to the nearest 1 mm with a Seca 212 measuring tape. Infant weight will be measured to the nearest 10 g using a Seca 384 scale and height will be measured to the nearest 1 mm using a Seca 416 infantometer. Infant chest circumference and MUAC will be measured to the nearest 1 mm with a Seca 212 measuring tape. At birth, the infant’s anthropometric measurements will be taken within 6 h of birth. Measurements will be taken in duplicate, and a third measurement will be taken if there is a large discrepancy between measures (e.g., >0.3 kg for weight, >3 cm for height, and >1 cm for MUAC) between the first two measurements.

Clinical measurements (e.g., signs of fever, vomiting, diarrhea, cough, difficulty breathing, and running nose) will be taken along with the anthropometric measures at each visit. Additionally, at birth, the infant’s nutritional and health status will be assessed using the Integrated Management of Neonatal and Childhood Illness approach. Cardiovascular evaluation (APGAR score [30]) will be taken within 1 min of birth, and a neurological and physical maturity examination (Dubowitz score [31]) will be carried out within 12 h of birth.

***Household visit questionnaires***

Socioeconomic and demographic information from all participants will be collected during the first household visit (e.g., 33–34 weeks of gestation). The project sociologist will ask questions on household members’ characteristics, household possessions, household food security, and water sanitation and hygiene (WaSH), which is a framework used to assess the quality and accessibility of water sanitation and hygiene facilities and services. A diet quality survey will be used to assess the dietary diversity of the women.

#### 2.3.2. Cord Blood

Within 30 min of birth, 5 mL of arterial whole blood will be collected from the umbilical cord by puncturing the artery with a needle. From this, 500 µL will be transferred into BD Vacutainer^®^ plastic whole blood tubes with spray-coated K2 potassium salt of ethylene diamine tetra acetic acid (EDTA) (BD, Franklin Lakes, NJ, USA), and one drop will be used to collect a 10 µL sample with the volumetric absorptive microsampling (VAMS) kit. The VAMS devices will be obtained from Neoteryx (Torrance, CA, USA).

The remaining blood will be transferred into a 4 mL EDTA tube and then further transferred to two 2 mL sterile cryotubes (Biosigma, Cona, VE, Italy) after gently mixing by tilting it ten times. The 500 µL microtainer EDTA tube, 10 µL VAMS, and the two 2 mL cryotubes will be stored at −80 °C until transfer to the designated laboratories for analysis.

The 500 µL samples will be centrifuged using a microcentrifuge (VWR International, Leuven, Belgium) to obtain plasma samples, which will be used for cytokines and chemokines, immunophenotyping, and proteomics analysis. The two 2 mL samples will be used for black carbon, mitochondrial DNA content, and telomere length analyses using a previously validated method [32]. The 10 µL VAMS will be used for untargeted metabolomics analysis.

#### 2.3.3. Capillary Blood

Capillary blood samples will be collected from mothers on the day of delivery and from infants at Dol 1, 3, 4, 7, 30, and 60. The collection zones for mothers are fingers, and for infants are heels. A total of 500 µL of blood will be collected from the incision site onto an EDTA microtainer tube. After this, 40 µL and 20 µL of blood samples will be collected using the VAMS kit. The 500 µL microtainer EDTA tube and VAMS samples will be stored at −80 °C before they are transferred to laboratories.

The 500 µL samples will be centrifugated to obtain plasma samples for cytokines and chemokines, immunophenotyping, and proteomics analysis. The 20 µL VAMS will be used for mycotoxins analysis, and the 10 µL VAMS will be used for untargeted metabolomics analysis.

#### 2.3.4. Placental Biopsy

The placenta will be placed on a tray with the fetal side facing up, and then any excess blood will be cleaned off using phosphate-buffered saline-soaked sterile gauze. An area approximately 2 cm from the umbilical cord, between the large veins, will be identified for sampling. A 1.5 cm deep strip of placental tissue will be cut using the scalpel sleeve. Two tissue samples will be collected, with each sample dipped into a phosphate-buffered saline-filled falcon tube and shaken gently to remove maternal blood. This process will be repeated three times, and the cleaned samples will then be placed into two 2 mL cryotubes. Samples will be stored in a −80 °C freezer before they are transferred to the laboratory. The placental samples will be used for DNA adductomics analysis.

#### 2.3.5. Vaginal Samples

Vaginal samples will be collected at 29–30 weeks and 33–34 weeks of gestation and on the day of delivery using sterile swabs. The sampling is performed by gently scraping both vaginal walls in circular motions for at least 20 s. The swab will be inserted into a 2 mL cryotube, cut to fit, and the tube will be tightly sealed. On the day of delivery, before membrane rupture, an OMR-130 kit (DNA Genotek^®^ Ottawa, ON, Canada) will also be used to collect vaginal samples following the same swabbing process. The samples will then be inserted into the stabilizing liquid tube and sealed. The samples will be stored in the −80 °C freezer before they are transferred to the laboratory. These swab samples will be used for cytokines and chemokines analyses, and the OMR-130 kit samples will be used for vaginal metagenomic analysis.

#### 2.3.6. Breastmilk Samples

Colostrum samples will be collected by manual expression at Dol0 or Dol1 at four intervals within the first 24 h postpartum, and transitional milk will be collected by manual expression at Dol 3 and 5 at one-time point. The healthcare worker will ask the participant which breast was last used for breastfeeding and prepare the opposite breast for collection. For colostrum, a total of 10 mL samples will be collected by manual expression, and then the samples will be transferred into four 2 mL cryotubes. For transitional and mature milk, samples will be collected at Dol7, 14, 30, and 60 using an electric breast pump (Medela, Baar, Switzerland). Samples from the opposite breast last used for feeding will be fully expressed into a 150 mL container, then transferred into four 2 mL cryotubes. If less than 25 mL is collected, the second breast will be expressed. All milk samples will be snap-frozen within 4 h in liquid nitrogen for transfer to the local storage facility, where they will be kept at −80 °C until they are transferred to partner laboratories for analysis.

#### 2.3.7. Saliva Samples

Saliva samples will be collected from mothers and infants at Dol1, 2, 3, 4, 5, 14, and 15. The deuterium oxide (D_2_O) method is used to assess breastmilk intake in infants and to assess body composition in mothers. An oral administration of a small dose of deuterium oxide will be given to the mothers after collecting pre-dose saliva samples at Dol1, and post-dose samples will be collected on Dol2, 3, 4, 5, 14, and 15. To collect saliva samples, mothers will be asked to chew a cotton ball for 2 min, and for infants, trained study staff will use a cotton ball around a straw to collect saliva from inside of their cheeks. After that, saliva will be extracted from cryotubes (20 mL for mothers and 10 mL for infants) using syringes. Samples will be stored in the −80 °C freezer before they are transferred to the laboratory.

#### 2.3.8. Stool Samples

Maternal stool samples will be collected at 24–30 weeks, 33–34 weeks of gestation, on Dol7, 30, 60, and 180 using a stool collection container, and infant stool samples will be collected daily after birth until Dol7, and on Dol 15, 30, 60 and 180 using a sterile protection sheet, wrapped around the infant, functioning like a diaper. After collection, samples will be aliquoted into four 2 mL sterile cryotubes and stored in the −80 °C freezer before they are transferred to the laboratory. Stool samples will be used for shotgun metagenomics, untargeted proteomics and metabolomics analyses, and enteropathogen analysis.

For all detailed instructions and standard operating procedures related to sample collection, refer to the Supplementary Materials.

### 2.4. Laboratory Analysis

#### 2.4.1. Cytokine and Chemokine Analyses

Cytokine and chemokine analyses will be performed on plasma and vaginal samples. Cytokine, chemokine, and immunological biomarkers from plasma samples will be characterized using electrochemiluminescence and the NULISAseq 250-plex inflammation panel [33], including key analytes critical to the inflammation response, immune system regulation, and various related biological processes. 

Cytokines in vaginal or cervicovaginal secretions previously associated with preterm birth will be assessed, including IL-1β, IL-6, IL-17A, and IL-8 [34,35,36,37,38]. In addition, IP-10, a chemokine actively downregulated by dysbiotic microbiota associated with bacterial vaginosis (BV), will be assayed. Concentrations will be measured from lateral vaginal wall swabs using a customized R&D system 6-plex assay, which includes inflammatory (IL-1α, IL-1β, IL-6), adaptive (IL-17A) cytokines, and chemokines (IL-8, IP-10).

#### 2.4.2. Immunophenotyping

Immunophenotyping from blood samples will be conducted using flow cytometry, analyzing T cells, B cells, Monocytes, NK cells, DCs, and granulocytes. This will include the following analysis:

- Analysis of B cell subsets (transitional, naive, memory, plasma blasts);
- Analysis of T cell subsets (naive, memory, effector);
- Analysis of T cell activation state (activated, exhausted);
- Analysis of T helper cell differentiation (Tregs, Th1 and Th17);
- Analysis of monocyte subsets (classical, inflammatory, and patrolling);
- Analysis of DC subsets (plasmacytoid DCs, cDC2, and cDC3);
- Analysis of granulocytes (neutrophils, basophils, eosinophils);
- Analysis of granulocyte maturation;
- Analysis of activation markers (CD64, HLA-DR, CD25, CD38); and,
- Analysis of NK cell subsets (CD56 and CD16 populations).

#### 2.4.3. Microbiome Composition and Functionality Analyses

Microbiome composition and functionality will be characterized in vaginal, breastmilk, and stool samples.

Microbial communities from these body sites will be characterized using shotgun metagenomic sequencing [39], a high-throughput sequencing approach that enables comprehensive profiling by sequencing all DNA present in a sample. This method allows for the identification of microbial species and strains, as well as functional profiling through the detection of genes associated with specific pathways. DNA extraction will be carried out following standardized protocols to ensure high-quality input for library preparation. Sequencing will be performed using the Illumina platform, which generates paired-end reads with high accuracy and depth. Bioinformatic analysis will include quality control, assembly, taxonomic classification, and functional annotation.

#### 2.4.4. TaqMan Array Card for Enteropathogen Detection

Enteropathogen detection will be conducted using the TaqMan Array Card (TAC) system in a 384-well real-time PCR format to detect 62 infection targets, including viruses, bacteria, protozoa, and helminths, specifically: *A. duodenale* and *lumbricoides*; *B. fragilis* and *hominis*; *C. belli*, *cayetanensis*, *coli*, *concisus*, *difficile*, *hominis*, *jejuni*, *parvum*, *troglodytis*, and *upsaliensis*; *E. bieneusi* and *histolytica*; *G. lamblia*; *H. pylori*; *M. tuberculosis*; *N. americanus*; *P. shigelloides*; *S. enterica*, *flexneri*, *mansoni*, *sonnei*, and *stercoralis*; *T. solium* and *trichiura*; *V. cholerae*; *enteroaggregative E. coli* [*EAEC* (*aaiC*, *aatA*, and *aagR*)], *enteroinvasive E. coli* [EIEC (*Shingella* spp.)], *enteropathogenic E. coli* [EPEC (bfp1 and eae)], *enterotoxigenic E. coli* [ETEC (LT, STh, and STp)], and *Shiga toxin* (stx1 and stx2) producing *E. coli*; *Campylobacter* pan. and *Entamoeba* pan.; *Aeromonas* spp., *Cryptosporidium* spp., *Encephalitozoon* spp., and *Schistosoma* spp.; *adenovirus* (serotypes 40 and 41), *astrovirus*, *Epstein-Barr virus* (EBV), *norovirus* (GI/GII and GI.1/GII.4), *rotavirus*, and *sapovirus*; antibiotic resistance genes, including: β-lactam (CTX-M, TEM, SHV), carbapenemases/carbapenems (KPC, NDM, MCR-1, OXA), macrolide (ermB, mphA), and quinolone (QnrA, QnrB1, QnrB4, QnrS).

#### 2.4.5. Proteomic Analyses

Proteomic analyses will be conducted on blood, breastmilk, and stool samples to comprehensively characterize protein profiles.

Untargeted proteomics analyses for blood and milk samples will be conducted using liquid chromatography-mass spectrometry (LC-MS) on a harmonized Orbitrap ExplorisTM instrument. This system features an EasySpray ion source and is integrated with either an UltiMateTM 3000 Nano LC (Thermo Fisher Scientific, Waltham, MA, USA) or an Evosep system, ensuring high-resolution and reproducible protein identification and quantification. This approach allows for the in-depth exploration of proteomic landscapes in blood and milk samples.

For a subset of stool samples, proteomic analysis will be conducted using mass spectrometry-based methods to identify and quantify proteins in the stool samples, providing insights into microbial and host protein expression [40,41].

#### 2.4.6. Metabolomic Analyses

Metabolomic analyses will be performed on blood, breastmilk, and stool samples to comprehensively assess metabolic profiles.

For blood and breastmilk samples, untargeted metabolomic analyses will be conducted by next-generation rapid liquid chromatography-mass spectrometry (rLC-MS) with a modified Agilent RapidFire 360 sample injector coupled to a high-resolution Agilent 6545B QToF mass spectrometer (Agilent Technologies, Santa Clara, CA, USA). This advanced system integrates the high-throughput separation capabilities of rapid liquid chromatography with the precision and sensitivity of mass spectrometry, enabling the unbiased identification and quantification of a wide array of metabolites.

Metabolomic profiling of a subset of stool samples will also be conducted using LC-MS. This method enables the detection and quantification of metabolites that play critical roles in microbial and host metabolic pathways [40,41].

#### 2.4.7. Breastmilk Analyses

Except for the microbiome, proteomic, and metabolomic analyses described earlier, breastmilk samples will be analyzed for their comprehensive composition using the established International Milk Composition (IMiC) Consortium pipeline. Key components of breastmilk will be quantified as follows:

Macronutrients (lipids, proteins, and carbohydrates) will be measured using near-infrared (NIR) spectroscopy [42], enabling precise quantification of energy-providing nutrients.

Vitamins and minerals will be analyzed using LC-MS and inductively coupled plasma mass spectrometry (ICP-MS), respectively [43].

HMOs and bioactive proteins will be analyzed using LC-MS [43] and electrochemiluminescence (ECL) [44], respectively.

#### 2.4.8. Saliva Analyses

Deuterium oxide concentrations in saliva samples will be measured to assess water turnover and hydration status. This analysis will be conducted by Fourier-transformed infrared (FTIR) spectrophotometry using an Agilent 4500 Series device (Agilent Technologies, Santa Clara, CA, USA). The FTIR spectrophotometer will measure the absorbance of infrared light by deuterium oxide at specific wavelengths. Calibration curves will be constructed using deuterium standards to convert absorbance values into precise concentrations.

#### 2.4.9. Multiple Mycotoxin Profiling

VAMS samples will be used for multiple mycotoxin profiling. Analysis was conducted using a Waters UPLC^®^ system coupled to a Quattro XEVO TQ-XS mass spectrometer (Waters, Manchester, UK). The samples will be detected by UHPLC-MS/MS, which is able to provide the necessary sensitivity at ppb-level [45]. This combination of Ultra-High-Performance Liquid Chromatography (UHPLC) and mass spectrometry ensures robust separation and precise quantification of mycotoxins in blood samples, providing highly accurate and reliable results.

#### 2.4.10. Black Carbon Exposure

Analysis of black carbon will be conducted on umbilical cord blood samples. Two samples of 2 mL arterial umbilical cord blood will be aliquoted, snap-frozen, and stored at −80 °C, then transferred to the University of Hasselt. The samples will be prepared using particle-free instruments and sample holders in a clean room with filtered air to avoid particulate contamination. Black carbon will be detected through non-incandescence-related white-light generation under femtosecond pulsed illumination, as described in previous literature [46].

#### 2.4.11. Telomere Length and Mitochondrial DNA

Telomere length and mitochondrial DNA will be measured via a real-time PCR method using umbilical cord blood [32,47].

Placental and cord blood leukocyte DNA will be extracted using the QIAamp DNA Mini Kit (Qiagen, Inc., Venlo, The Netherlands). DNA quantity and purity will be assessed by a Nanodrop 1000 spectrophotometer (Isogen, Life Science, Belgium). To ensure a uniform DNA input of 5 ng for each qPCR reaction, samples will be diluted and checked using the Quant-iT™ PicoGreen^®^ dsDNA Assay Kit (Invitrogen, Thermo Fisher Scientific, Waltham, MA, USA). Measurements will be performed in triplicate on a 7900HT Fast Real-Time PCR System (Applied Biosystems, Thermo Fisher Scientific, Waltham, MA, USA) in a 384-well format.

#### 2.4.12. Placental DNA Adductomes

DNA adductome analysis will be conducted on placenta tissue, which provides insights into exposures affecting both mother and child [48,49]. In brief, 250 mg of placenta will be collected, snap-frozen, and stored at −80 °C. DNA will be extracted using commercially available kits (e.g., Qiagen), followed by thermal acidic DNA hydrolysis and solid-phase extraction DNA adduct purification, in line with previous studies [50,51]. Samples will be analyzed by means of the Exploris^TM^ (Thermo Fisher Scientific, Waltham, MA, USA), using an untargeted full scan and targeted analysis approach.

### 2.5. Data Quality Control

Field data will be collected using Survey Solutions v.12.5 on tablets and securely transferred to a dedicated server at Ghent University on a daily basis. Generic validation codes will be implemented to minimize the entry of implausible values and enhance data quality. A medical epidemiologist will perform automatic weekly data checks to identify any inconsistencies or incomplete entries, issuing real-time queries as needed. Any missing or inconsistent data will be sent back to the field for revision. Additionally, to ensure the accuracy of ultrasound images and GA estimations, an external gynecologist will regularly evaluate the examinations using a structured quality checklist and scoring sheet. The integration of field data, biological data, and sequencing data will be handled by an external team of specialized data scientists.

### 2.6. Ethical Considerations

The protocol of this study has been approved by the ethics committee of Ghent University Hospital in Belgium (approval number ONZ-2022-0500 issued on 29 November 2022) and the ethics committee of the Institut de Recherche en Sciences de la Santé in Burkina Faso (approval number 050-2022/CEIRES issued on 16 September 2022). Before any inclusion, participants will receive all the information on the objectives, methods, risks, and benefits of the study, as well as on their rights. An information sheet will be given to each participant. An informed consent form signed by the participant will certify receipt of this information and consent to participation in the study.

## 3. Strengths and Limitations

The DenBalo project stands out as the first study to generate a comprehensive dataset that covers multiple biological domains, including the gut microbiome, vaginal microbiome, breastmilk composition, and immunophenotyping. This multidimensional approach offers a unique opportunity to examine the intricate interplay between maternal and neonatal factors that influence early-life immune system development. Additionally, the study’s high-resolution sample collection—daily during the first week and at key time points during the first six months of life—allows for an unprecedented temporal analysis of the neonatal immune system in small, vulnerable newborns versus healthy controls, providing insights into how early life exposures affect long-term health outcomes.

Despite its strengths, the DenBalo study has several limitations. The relatively short investigation period limits the ability to thoroughly assess immune system development across the entire infancy period. Additionally, the study is monocentric and focuses on a largely homogenous population (urban and African), which may reduce the generalizability of the findings to other populations or contexts. Future research involving a longer follow-up period and more diverse populations is necessary to build on the study’s insights and broaden its applicability.

## Figures and Tables

**Table 1:** Denbalo inclusion and exclusion criteria.

Step	Inclusion Criteria	Exclusion Criteria
Screening	Women between 15 and 40 years old at study inclusion. Fundal height between 24 cm and 27 cm. Women living in the health zones of Accart-Ville, Colma 1, or Farakan Women not planning to give birth or move outside the study area in the first 6 months of the infant’s life Oral consent	Fundal height <24 cm or >27 cm Women living outside the sanitary zone of the Accart-Ville, Colma 1, or Farakan Women planning to give birth outside the study area or to move from it within the first 6 months of the infant’s life
Pre-inclusion	GA between 24 weeks 1 completed day and 29 weeks 6 days (ultrasound) Monofetal pregnancy without visible malformation Women agreeing to give their informed consent to participate in the study	GA <24 weeks or ≥30 weeks (ultrasound) Multi-fetal pregnancy Malformation visible on ultrasound
Inclusion	Women seen in labor before the rupture of membranes, thus allowing vaginal sampling Delivery of a live birth Vaginal birth Absence of severe infectious pathology, severe pneumopathy, or respiratory distress in the neonate Neonates who did not receive corticosteroids or antibiotics at birth *For SVN group:* Neonates born between the 34th and 37th weeks of pregnancy Or birthweight below the 10th centile of the recommended international, sex-specific birthweight for GA standard Or birth weight ≤2500 g *For healthy control group:* Neonates born after the 37th week of pregnancy Birth weight >2500 g	Women seen in labor after rupture of membranes Cesarean delivery Neonates with severe infectious disease, severe pneumopathy, or respiratory distress Neonates who received corticosteroids or antibiotics just after birth *For SVN group:* Neonates born before the 34th week of pregnancy Birth weight <1500 g

**Table 2:** The quality control criteria for TCD and FL assessment.

Assessments	Quality Control Criteria
TCD	Magnification: Proper zooming, with 30% of the image size occupied. Image plane: The image is frozen in the correct plane; the cerebellum must be clearly visible; the cerebellar hemispheres should appear symmetric, with the upper and lower hemispheres similar in size. Caliper placement: The top caliper should be placed on the outer margin of the upper cerebellar hemisphere; the lower caliper should be placed on the outer margin of the lower cerebellar hemisphere. Each image can achieve a maximum score of 5, based on these criteria.
FL	Magnification: Proper zooming, with 30% of the image size occupied. Image Plane: The image is frozen in the correct plane; the femur should be displayed fully, from side to side on the screen; only a single bone should be visible in this section of the extremity; the upper femur must be measured. Caliper placement: Calipers should be placed at the outer margins of the echogenic bone (outer-to-outer); secondary ossification centers should not be included in the measurement. Each image can receive a maximum score of 7 based on these criteria.

TCD: transcerebellar diameter; FL: femur length.

**Table 3:** Data collection schedule of DenBalo study.

	24–30 Weeks	33–34 Weeks	36 Weeks	Birth (Dol0)	Dol1	Dol2	Dol3	Dol4	Dol5	Dol6	Dol7	Dol14	Dol15	Dol30	Dol60	Dol180
**Metadata**																
Anthropometry	M		M	D	D	D	D	D	D	D	D	D	D	D	D	D
Clinical Data	M		M	D	B	B	B	B	B	B	B	B	B	B	B	B
Integrated Management of Newborn and Childhood Illness (IMNCI)				B												
Dubowitz score, APGAR score				B												
Household Food Insecurity Access Scale (HFIAS)		M														
Diet Quality Questionnaire (DQQ)		M														
Socioeconomic status		M														
Water Sanitation and Hygiene (WaSH)		M														
**Blood**																
Plasma (cytokines and chemokines)				D ^1^	B		B		B		B			B	B	
Plasma (immunophenotyping)				D ^1^	B		B		B		B			B	B	
Plasma (proteomics)				D ^1^	B		B		B		B			B	B	
Whole blood (black carbon exposure)				B ^1^												
Whole blood (mitochondrial DNA, telomere length)				B ^1^												
Whole blood on VAMS (mycotoxins)				M							B		B			
Whole blood on VAMS (untargeted metabolomics)				D ^1^	B		B		B		B					
**Placental Biopsies**																
DNA adductomics				M												
**Vaginal Swabs**																
Cytokines/chemokines	M	M														
Shotgun metagenomics	M	M		M												
**Breastmilk**																
Macronutrients				M	M		M		M		M		M	M	M	
Vitamins				M	M		M		M		M		M	M	M	
Minerals				M	M		M		M		M		M	M	M	
Metagenomics, proteomics, and metabolomics				M	M		M		M		M		M	M	M	
HMOs and bioactive proteins				M	M		M		M		M		M	M	M	
**Saliva**					D	D	D	D	D			D	D			
**Stool**																
Shotgun metagenomics	M	M		B	B	B	B	B	B	B	D		B	D	D	D
Untargeted proteomics	M	M		B	B	B	B	B	B	B	D		B	D	D	D
Untargeted metabolomics	M	M		B	B	B	B	B	B	B	D		B	D	D	D
TaqMan Array Card (enteropathogens)	M	M												D		D

^1^ Umbilical cord artery. M: mother; B: baby; D: mother and baby dyads.

## Data Availability

Given the personal nature of the data, data will be made available. through a data-sharing agreement. Please contact trenton@dailey-chwalibog.com for any queries.

## Supplementary Materials

The following supporting information can be downloaded at: https://www.mdpi.com/article/10.3390/nu16234242/s1.

## Abbreviations

AMANHI	alliance for maternal newborn health improvement, 6
CD	cluster of differentiation, 12
cDC	conventional dendritic cell, 12
CMA	centre médical avec antenne chirurgicale, 5
CSPS	centre de santé de promotion sociale, 5, 6
DC	dendritic cell, 12
DCs	dendritic cells, 12
DNA	deoxyribonucleic acid, 14
EBV	Epstein-Barr virus, 13
EDTA	ethylene diamine tetra acetic acid, 9
FL	femur length, 6, 8
GA	gestational age, 6, 7, 15
GM-CSF	granulocyte-macrophage colony stimulating factor, 12
HLA-DR	human leukocyte antigen—DR isotype, 12
HMOs	human milk oligosaccharides, 3
IFN-γ	interferon gamma, 12
IL	interleukin, 12
KPC	Klebsiella pneumoniae carbapenemase, 13
LBW	low birth weight, 2, 3
LC-MS	liquid chromatography-mass spectrometry, 13
MCP-1	monocyte chemoattractant protein-1, 12
MCP-4	monocyte chemoattractant protein 4, 12
MCR	mobilized colistin resistance gene, 13
MDC	macrophage-derived chemokine, 12
MIP	macrophage inflammatory protein, 12
MUAC	mid-upper arm circumference, 8
NDM	New Delhi metallo-β-lactamase, 13
NK	natural killer, 12
PCR	polymerase chain reaction, 12, 14
ppb	parts per billion, 14
Qnr	quinolone resistance gene, 13
qPCR	quantitative polymerase chain reaction, 14
rLC-MS	rapid liquid chromatography-mass spectrometry, 13
SGA	small-for-gestational-age, 2, 3
SVN	small vulnerable newborn, 6, 7
SVNs	small vulnerable newborns, 2, 3, 4, 5
TAC	TaqMan array card, 12
TARC	thymus and activation regulated chemokine, 12
TCD	transcerebellar diameter, 6, 8
Th1	type 1 T helper cell, 12
Th17	type 17 T helper cell, 12
TNF	tumor necrosis factor, 12
Tregs	Regulatory T cells, 12
TSLP	thymic stromal lymphopoietin, 12
VAMS	volumetric absorptive microsampling, 11

## References

[1] Ashorn P., Ashorn U., Muthiani Y., Aboubaker S., Askari S., Bahl R., Black R.E., Dalmiya N., Duggan C.P., Hofmeyr G.J. (2023). Small vulnerable newborns-big potential for impact. Lancet.

[2] World Health Organization Neonatal Mortality Rate (per 1000 Live Births) [Internet]. Datadot. https://data.who.int/indicators/i/E3CAF2B/A4C49D3.

[3] Barro M. (2020). Morbidité et Mortalité néonatales au Centre Hospitalier Universitaire Sourô Sanou Bobo-Dioulasso (Burkina Faso). Rev. Afr. Malgache Rech. Sci. Santé.

[4] Bountogo M., Sié A., Zakané A., Compaoré G., Ouédraogo T., Lebas E., Brogdon J., Nyatigo F., Arnold B.F., Lietman T.M. (2021). Antenatal care attendance and risk of low birthweight in Burkina Faso: A cross-sectional study. BMC Pregnancy Childbirth.

[5] Lingani M., Zango S.H., Valéa I., Somé G., Sanou M., Samadoulougou S.O., Ouoba S., Rouamba E., Robert A., Dramaix M. (2022). Low birth weight and its associated risk factors in a rural health district of Burkina Faso: A cross sectional study. BMC Pregnancy Childbirth.

[6] Millogo Traoré T., Sawadogo O., Zongo Kondé S. (2022). Evaluation of the Fetal Neuroprotection Protocol with Magnesium Sulphate in a University Hospital in Burkina Faso. Open J. Obstet. Gynecol..

[7] De Kok B., Toe L.C., Hanley-Cook G., Argaw A., Ouédraogo M., Compaoré A., Vanslambrouck K., Dailey-Chwalibóg T., Ganaba R., Kolsteren P. (2022). Prenatal fortified balanced energy-protein supplementation and birth outcomes in rural Burkina Faso: A randomized controlled efficacy trial. PLoS Med..

[8] Lee A.H., Shannon C.P., Amenyogbe N., Bennike T.B., Diray-Arce J., Idoko O.T., Gill E.E., Ben-Othman R., Pomat W.S., van Haren S.D. (2019). Dynamic molecular changes during the first week of human life follow a robust developmental trajectory. Nat. Commun..

[9] Mueller N.T., Shin H., Pizoni A., Werlang I.C., Matte U., Goldani M.Z., Goldani H.A., Dominguez-Bello M.G. (2017). Delivery Mode and the Transition of Pioneering Gut-Microbiota Structure, Composition and Predicted Metabolic Function. Genes.

[10] Makino H., Kushiro A., Ishikawa E., Muylaert D., Kubota H., Sakai T., Oishi K., Martin R., Ben Amor K., Oozeer R. (2011). Transmission of intestinal *Bifidobacterium longum* subsp. longum strains from mother to infant, determined by multilocus sequencing typing and amplified fragment length polymorphism. Appl. Environ. Microbiol..

[11] Makino H., Kushiro A., Ishikawa E., Kubota H., Gawad A., Sakai T., Oishi K., Martin R., Ben-Amor K., Knol J. (2013). Mother-to-infant transmission of intestinal bifidobacterial strains has an impact on the early development of vaginally delivered infant’s microbiota. PLoS ONE.

[12] Jost T., Lacroix C., Braegger C.P., Rochat F., Chassard C. (2014). Vertical mother-neonate transfer of maternal gut bacteria via breastfeeding. Environ. Microbiol..

[13] Funkhouser L.J., Bordenstein S.R. (2013). Mom knows best: The universality of maternal microbial transmission. PLoS Biol..

[14] Bittinger K., Zhao C., Li Y., Ford E., Friedman E.S., Ni J., Kulkarni C.V., Cai J., Tian Y., Liu Q. (2020). Bacterial colonization reprograms the neonatal gut metabolome. Nat. Microbiol..

[15] Ma J., Li Z., Zhang W., Zhang C., Zhang Y., Mei H., Zhuo N., Wang H., Wang L., Wu D. (2020). Comparison of gut microbiota in exclusively breast-fed and formula-fed babies: A study of 91 term infants. Sci. Rep..

[16] Granger C.L., Embleton N.D., Palmer J.M., Lamb C.A., Berrington J.E., Stewart C.J. (2021). Maternal breastmilk, infant gut microbiome and the impact on preterm infant health. Acta Paediatr..

[17] Groer M.W., Luciano A.A., Dishaw L.J., Ashmeade T.L., Miller E., Gilbert J.A. (2014). Development of the preterm infant gut microbiome: A research priority. Microbiome.

[18] Melville J.M., Moss T.J.M. (2013). The immune consequences of preterm birth. Front. Neurosci..

[19] Phattraprayoon N., Kraisonsin N., Kanjanapattanakul W. (2018). Comparison of Breast Milk Compositions Among Mothers Delivering Small-for-Gestational Age, Appropriate-for-Gestational Age, and Large-for-Gestational Age Infants. Breastfeed Med..

[20] Imdad A., Bhutta Z.A. (2013). Nutritional Management of the Low Birth Weight/Preterm Infant in Community Settings: A Perspective from the Developing World. J. Pediatr..

[21] Ma U. (2013). Human milk for the premature infant. Pediatr. Clin. N. Am..

[22] Bode L. (2012). Human milk oligosaccharides: Every baby needs a sugar mama. Glycobiology.

[23] Za B., Re B. (2013). Global maternal, newborn, and child health—So near and yet so far. N. Engl. J. Med..

[24] Chu H., Mazmanian S.K. (2013). Innate immune recognition of the microbiota promotes host-microbial symbiosis. Nat. Immunol..

[25] Kollmann T.R., Kampmann B., Mazmanian S.K., Marchant A., Levy O. (2017). Protecting the Newborn and Young Infant from Infectious Diseases: Lessons from Immune Ontogeny. Immunity.

[26] Bennike T.B., Fatou B., Angelidou A., Diray-Arce J., Falsafi R., Ford R., Gill E.E., van Haren S.D., Idoko O.T., Lee A.H. (2020). Preparing for Life: Plasma Proteome Changes and Immune System Development During the First Week of Human Life. Front. Immunol..

[27] Nayak S., Welling J., Burd I. (2017). Maternal Immunomodulation Therapy for Prevention of Preterm Birth and Prematurity-Related Morbidity: The New Era of Immuno-Perinatology. Curr. Pharm. Des..

[28] Solyman A., Shaban D., Abdullah M., Hosni N., Mahmoud S. (2022). Ultrasound-determined fetal transcerebellar diameter in relation to gestational age during third trimester of pregnancy. Menoufia Med. J..

[29] World Health Organization (2020). Performance of late pregnancy biometry for gestational age dating in low-income and middle-income countries: A prospective, multicountry, population-based cohort study from the WHO Alliance for Maternal and Newborn Health Improvement (AMANHI) Study Group. Lancet Glob. Health.

[30] Simon L.V., Shah M., Bragg B.N. (2024). APGAR Score. StatPearls [Internet].

[31] Dubowitz L.M.S., Dubowitz V., Goldberg C. (1970). Clinical assessment of gestational age in the newborn infant. J. Pediatr..

[32] Hanley-Cook G.T., Bastos-Moreira Y., Martens D.S., Dailey-Chwalibóg T., Toe L.C., de Kok B., Ouédraogo L., Argaw A., Tesfamariam K., Kolsteren P. (2023). Prenatal multiple micronutrient-fortified balanced energy-protein supplementation and newborn telomere length and mitochondrial DNA content: A randomized controlled efficacy trial in rural Burkina Faso. medRxiv.

[33] NULISAseq|Comprehensive Inflammation Panel [Internet]. Alamar Biosciences. https://alamarbio.com/products-and-services/nulisa-inflammation-panel/.

[34] Nold C., Barros A., Rogi C., Sulzer C., Quental A., Reid S., Serdah M., Vella A.T. (2022). Concentration of vaginal and systemic cytokines obtained early in pregnancy and their impact on preterm birth. J. Matern. Fetal Neonatal Med..

[35] Park S., You Y.-A., Yun H., Choi S.-J., Hwang H.-S., Choi S.-K., Lee S.M., Kim Y.J. (2020). Cervicovaginal fluid cytokines as predictive markers of preterm birth in symptomatic women. Obstet. Gynecol. Sci..

[36] Manning R., James C.P., Smith M.C., Innes B.A., Stamp E., Peebles D., Bajaj-Elliott M., Klein N., Bulmer J.N., Robson S.C. (2019). Predictive value of cervical cytokine, antimicrobial and microflora levels for pre-term birth in high-risk women. Sci. Rep..

[37] Amabebe E., Chapman D.R., Stern V.L., Stafford G., Anumba D.O.C. (2018). Mid-gestational changes in cervicovaginal fluid cytokine levels in asymptomatic pregnant women are predictive markers of inflammation-associated spontaneous preterm birth. J. Reprod. Immunol..

[38] Discacciati M.G., Simoes J.A., Silva M.G., Marconi C., Brolazo E., Costa M.L., Cecatti J.G. (2011). Microbiological characteristics and inflammatory cytokines associated with preterm labor. Arch. Gynecol. Obstet..

[39] Olm M.R., Dahan D., Carter M.M., Merrill B.D., Yu F.B., Jain S., Meng X.D., Tripathi S., Wastyk H., Neff N. (2022). Robust Variation in Infant Gut Microbiome Assembly Across a Spectrum of Lifestyles. Science.

[40] Han S., Van Treuren W., Fischer C.R., Merrill B.D., DeFelice B.C., Sanchez J.M., Higginbottom S.K., Guthrie L., Fall L.A., Dodd D. (2021). A metabolomics pipeline for the mechanistic interrogation of the gut microbiome. Nature.

[41] Gonzalez C.G., Wastyk H.C., Topf M., Gardner C.D., Sonnenburg J.L., Elias J.E. (2020). High-Throughput Stool Metaproteomics: Method and Application to Human Specimens. MSystems.

[42] Smilowitz J.T., Gho D.S., Mirmiran M., German J.B., Underwood M.A. (2014). Rapid measurement of human milk macronutrients in the neonatal intensive care unit: Accuracy and precision of fourier transform mid-infrared spectroscopy. J. Hum. Lact..

[43] Hampel D., Dror D.K., Allen L.H. (2018). Micronutrients in Human Milk: Analytical Methods. Adv. Nutr..

[44] Ju H., Lai G., Yan F. (2017). Immunosensing for Detection of Protein Biomarkers.

[45] Bastos-Moreira Y., Argaw A., Dailey-Chwalibóg T., El-Hafi J., Olivier Ouédraogo L., Celine Toe L., De Saeger S., Lachat C., De Boevre M. (2024). Prenatal ochratoxin A exposure, birth outcomes and infant growth in rural Burkina Faso: A human biomonitoring sub-study from the MISAME-III trial. Emerg. Contam..

[46] Bové H., Bongaerts E., Slenders E., Bijnens E.M., Saenen N.D., Gyselaers W., Van Eyken P., Plusquin M., Roeffaers M.B.J., Ameloot M. (2019). Ambient black carbon particles reach the fetal side of human placenta. Nat. Commun..

[47] Martens D.S., Janssen B.G., Bijnens E.M., Clemente D.B.P., Vineis P., Plusquin M., Nawrot T.S. (2020). Association of Parental Socioeconomic Status and Newborn Telomere Length. JAMA Netw. Open.

[48] Jeong Y., Lee S., Kim S., Park J., Kim H.-J., Choi G., Choi S., Kim S., Kim S.Y., Kim S. (2018). Placental transfer of persistent organic pollutants and feasibility using the placenta as a non-invasive biomonitoring matrix. Sci. Total Environ..

[49] Myllynen P., Pasanen M., Pelkonen O. (2005). Human placenta: A human organ for developmental toxicology research and biomonitoring. Placenta.

[50] Hemeryck L.Y., Van Hecke T., Vossen E., De Smet S., Vanhaecke L. (2017). DNA adductomics to study the genotoxic effects of red meat consumption with and without added animal fat in rats. Food Chem..

[51] Bussche J.V., Hemeryck L.Y., Van Hecke T., Kuhnle G.G.C., Pasmans F., Moore S.A., Van de Wiele T., De Smet S., Vanhaecke L. (2014). O6-carboxymethylguanine DNA adduct formation and lipid peroxidation upon in vitro gastrointestinal digestion of haem-rich meat. Mol. Nutr. Food Res..

