# Supplementary material for: Describing Biological Vulnerability in Small, Vulnerable Newborns in Urban Burkina Faso (DenBalo): Gut Microbiota, Immune System, and Breastmilk Assembly
Source: Nutrients. 2024 Dec 9;16(23):4242. doi: 10.3390/nu16234242 (PMC11644820; doi:10.3390/nu16234242)
Supplement: Supplementary file 1 [file nutrients-16-04242-s001.zip › nutrients-3318587-Supplementary.pdf]

SUPPLEMENTARY MATERIAL

Table of Contents

COLLECTION OF BLOOD SAMPLES IN MOTHERS: STANDARD OPERATING PROCEDURES (SOP) FOR THE DENBALO STUDY 4

ABSTRACT 4
NOTES 5
MATERIALS 5
PROCEDURE 6
1. *General method for capillary sampling 6*
2. *Plasma sample collection 7*
3. *Volumetric absorptive microsampling (VAMS) collection 9*
4. *Samples transport and storage 11*
5. *Troubleshooting & error and interferences 11*

COLLECTION OF BLOOD SAMPLES IN NEONATES: STANDARD OPERATING PROCEDURES (SOP) FOR THE DENBALO STUDY 12

ABSTRACT 12
NOTES 13
MATERIALS 14
PROCEDURE 14
1. *General method for capillary sampling 14*
2. *Plasma sample collection 16*
3. *Volumetric absorptive microsampling (VAMS) collection 18*
4. *Samples transport and storage 20*
5. *Troubleshooting & error and interferences 20*

COLLECTION OF STOOL SAMPLES IN MOTHERS: STANDARD OPERATING PROCEDURES (SOP) FOR THE DENBALO STUDY 21

ABSTRACT 21
NOTES 22
MATERIALS 23
PROCEDURE 23
1. *Stool sample 23*
2. *Samples transport and storage 24*
3. *Troubleshooting & error and interferences 25*

COLLECTION OF STOOL SAMPLES IN NEONATES: STANDARD OPERATING PROCEDURES (SOP) FOR THE DENBALO STUDY 26

ABSTRACT 26
NOTES 27
MATERIALS 28
PROCEDURE 28
1. *Stool samples collection 28*
2. *Samples transport and storage 30*
3. *Troubleshooting, errors, and interferences 30*

COLLECTION OF BREASTMILK SAMPLES: STANDARD OPERATING PROCEDURES (SOP) FOR THE DENBALO STUDY 32

ABSTRACT 32
NOTES 33
MATERIALS 33
PROCEDURE 34
1. *Sterilizer components* *34*
2. *Sterilization of Symphony® Breast Pump Kit element* *40*
3. *Breastmilk samples collection procedure* *41*
4. *Storage* *43*
5. *Troubleshooting & error and interferences* *43*

COLLECTION OF COLOSTRUM SAMPLES: STANDARD OPERATING PROCEDURES (SOP) FOR THE DENBALO STUDY 44

ABSTRACT 44
NOTES 45
MATERIALS 45
PROCEDURE 46
1. *Breast massage* *46*
2. *Manual breast expression* *46*
3. *Aliquots preparation* *50*
4. *Storage* *50*
BIBLIOGRAPHY 51

IMMUNOSTAINING: STANDARD OPERATING PROCEDURES (SOP) FOR THE DENBALO STUDY 52

ABSTRACT 52
NOTES 53
MATERIALS 53
PROCEDURE 54
1. *Preparation of buffers* *54*
2. *Staining mixture* *54*
3. *Staining protocol* *55*

COLLECTION OF VAGINAL SAMPLES IN MOTHERS: STANDARD OPERATING PROCEDURE (SOP) FOR THE DENBALO STUDY 59

ABSTRACT 59
NOTES 60
MATERIALS 60
PROCEDURE 60
1. *Vaginal sampling with the OMR-130 kit* *61*
2. *Vaginal sampling with sterile swabs* *68*

COLLECTION OF UMBILICAL CORD BLOOD SAMPLES: STANDARD OPERATING PROCEDURE (SOP) FOR THE DENBALO STUDY 70

ABSTRACT 70
NOTES 71
MATERIALS 71
PROCEDURE 72
1. *Collection Procedure* *72*
2. *Storage* *74*

LATE-TERM PREGNANCY ULTRASOUND FOR ASSESSMENT OF GESTATIONAL AGE: STANDARD OPERATING PROCEDURES (SOP) FOR THE DENBALO STUDY 76

ABSTRACT 76
NOTES 77

MATERIALS 77
PROCEDURE 78
1. *Biparietal Diameter (BPD)* *78*
2. *Head circumference (HC)* *80*
3. *Transcerebellular diameter (TCD)* *82*
4. *Abdominal circumference (AC)* *83*
5. *Femur length (FL)* *85*

DEUTERIUM OXIDE (D2O) WEIGHING AND LABELING : STANDARD OPERATING PROCEDURES (SOP) FOR THE DENBALO STUDY 87

ABSTRACT 87
NOTES 88
MATERIALS 88
PROCEDURE 88

COLLECTION OF SALIVA SAMPLES: STANDARD OPERATING PROCEDURES (SOP) FOR 90

DENBALO STUDY 90

ABSTRACT 90
NOTES 91
MATERIALS 91
PROCEDURE 92
1. *Perform anthropometric measurements in mothers and children* *92*
2. *Conditions for deuterium oxide administration* *92*
3. *Collecting pre-dose saliva* *92*
4. *Administering the deuterium dose to the mother* *94*
5. *Collection of the first post-dose saliva sample ("saliva J001, J003, J004, J013, J014")* *94*
6. *Preserve samples for transport and analysis* *95*

COLLECTION OF PLACENTA SAMPLES: STANDARD OPERATING PROCEDURE (SOP) FOR THE DENBALO STUDY 96

ABSTRACT 96
NOTES 97
MATERIALS 97
PROCEDURE 98
1. *Collection of placenta samples* *98*
2. *Sample transport and storage* *100*

Collection of blood samples in mothers: Standard Operating Procedures (SOP) for the DenBalo Study

Lionel Olivier Ouédraogo,[1,2] Trenton Dailey-Chwalibóg,[1] Kim Lagerborg,[3] Lori Glenwinkel,[4] Annie Moradian,[5] Shruti Rao,[5] Moctar Ouédraogo,[6] Cheick Ahmed Ouattara,[6,7] Anderson Compaoré,[6] Laéticia Céline Toé[1 8], Carl Lachat,[1]

Abstract

Standard operating procedures are crucial to guarantee sample collection consistency and quality during a study. The importance of high-quality biological samples is amplified by researchers' pursuit of a multi-omics approach. Many studies have been carried out in developed countries explaining the procedures used for biological samples collection for multi-omics analysis. However, a little or none of those studies were carried out in Africa where working conditions are totally different and sometimes rudimentary.

This standard operating procedure (SOP) provides a guideline for blood samples collection in mothers enrolled in the study "Description and Comparison of Biological Vulnerability in Small, vulnerable newborns versus Healthy community controls in Urban Burkina Faso (DenBalo) Study: Gut Microbiota, Immune System, and Breastmilk Assembly and Development in the First Days and Weeks of Life" (ONZ-2022-0500, 050-2022/CEIRES du 16 September 2022). Mothers' blood samples are dedicated to immunological analysis (cytokines, chemokines and immunostaining analysis), to mycotoxin profiling and to omics study namely metabolomics and proteomics.

The aim of this SOP is to establish a standardized process for blood samples collection in mothers ensuring the process consistency and the quality control of the samples collected. To that purpose this SOP details steps to follow to guarantee a good sample collection including material and tools preparation, specimen collection, labeling, handling and storage, and the list of required tracking documents to fill out during the process. By following this SOP, healthcare professionals can optimize the quality and reliability of blood samples. Our SOP could be a reference guideline on blood samples collection in Africa specifically tailored for proteomics and metabolomics analysis.

Keywords: Standard operating procedure, blood samples, mothers, proteomics, metabolomics, mycotoxin profiling, immunostaining.

[1] Department of Food Technology, Safety and Health, Faculty of Bioscience Engineering, Ghent University, Ghent, Belgium
[2] Centre Muraz, Bobo-Dioulasso, Burkina Faso
[3] Broad Institute of Harvard and MIT, Cambridge, MA 02142, USA
[4] Department of Biological Sciences, Columbia University, Howard Hughes Medical Institute, New York, United States.
[5] Precision Biomarker Laboratories, Beverly Hills, CA 90211, USA
[6] Agence de Formation de Recherche et d'Expertise en Santé pour l'Afrique (AFRICSanté)
[7] École Doctorale de Santé Publique, Université Nazi Boni, Bobo-Dioulasso, Burkina Faso
[8] Unité Nutrition et Maladies Métaboliques, Institut de Recherche en Sciences de la Santé (IRSS)

Notes

- Do not exceed two attempts to collect blood.
- VAMS collection follows plasma collection if the blood is not clotted and continues to flow in sufficient quantity after plasma collection. If not, apply the plaster to the previously pricked finger and select the third or fourth finger of the opposite hand and repeat all steps of the general capillary collection method.
- Always begin collection of VAMS with 20µL VAMS and end with 10µL VAMS.

MATERIALS

MATERIALS	QUANTITIES
Pen	01
Soap	01
Romed® Alcohol Pad	At least 06 pads
Xylocaïne® Anesthesia gel 2%	01 tube
Cotton ball	As many as needed
Blue contact-activated bd microtainer® lancet	02
500-µl violet BD microtainer EDTA tube	01
Delta-T® cooler bag (10L)	01
Neoteryx® microsampler (VAMS) 10µl	02
Neoteryx® microsampler (VAMS) 20µl	02
Leukoplast soft plaster (19mm x 72mm)	02
Biohazard needle container	01
Cryolabels	01 sheet
Gloves	02 pairs
Samsung Tablet	01

PROCEDURE

1. General method for capillary sampling

- The participant and the health worker should wash their hands thoroughly with soap and water.
- Apply a thin layer of xylocaine® 2% anesthetic gel on the surface of the finger selected for sampling (preferably the third finger) at least 15 minutes before sampling. Choose the third finger of the hand less used by the participant for sampling.
- NB: In anticipation, apply a thin layer of xylocaine ® 2% anaesthetic gel on the surface of the finger selected on the opposite hand (the third finger of the opposite hand to the first). This finger will be used for blood collection if the first blood collection attempt fails.
- Clean the site with an alcohol swab and let it dry completely.
- Prick the bulging area on the fingertip of the third finger (first choice) or fourth finger of the lesser used hand. Place the blue contact-activated bd microtainer® lancet firmly on the puncture site. Do not remove the lancet until you hear an audible click (Figure 1)

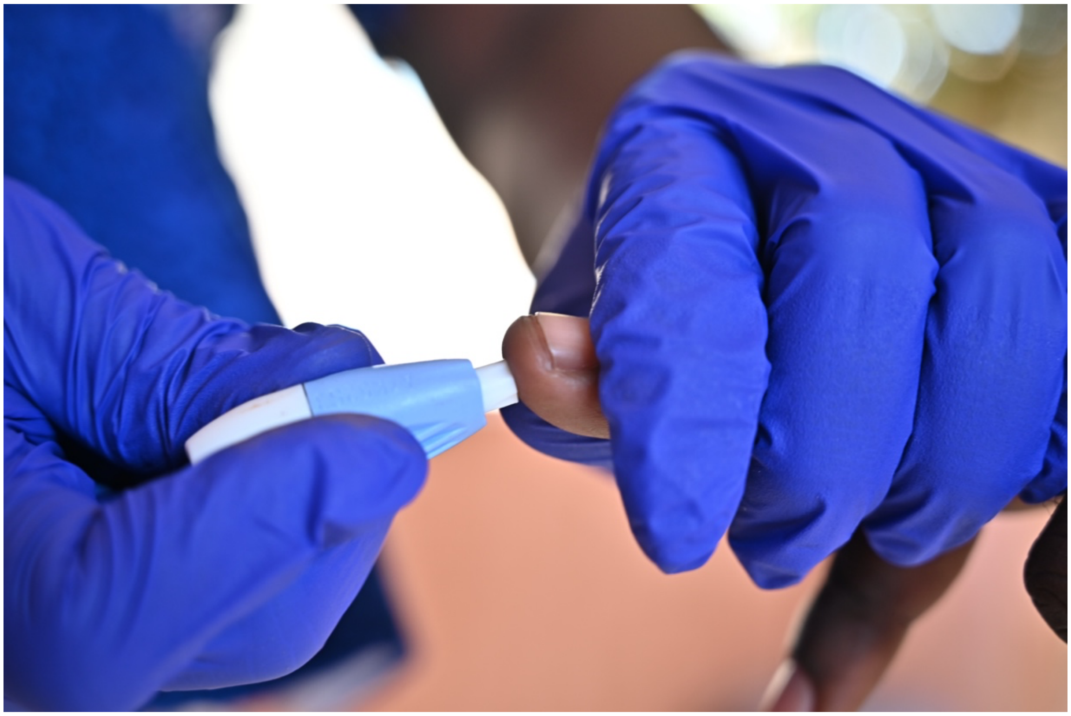

Figure 1: Use of the blue contact-activated lancet BD microtainer

- Wipe off the first drop of blood with a cotton ball.
- To facilitate the process, gently massage the finger.

2. Plasma sample collection

- Position the participant's hand the palm side facing the ground.
- Hold the 500-µl violet BD microtainer EDTA tube at a 30° to 45° angle to the surface of the puncture site (Figure 2). Touch the collection end of the tube to drop the blood. Avoid scratching the skin surface to collect the blood sample. After collecting 2 or 3 drops, the blood will flow freely down the wall of the tube to the bottom.

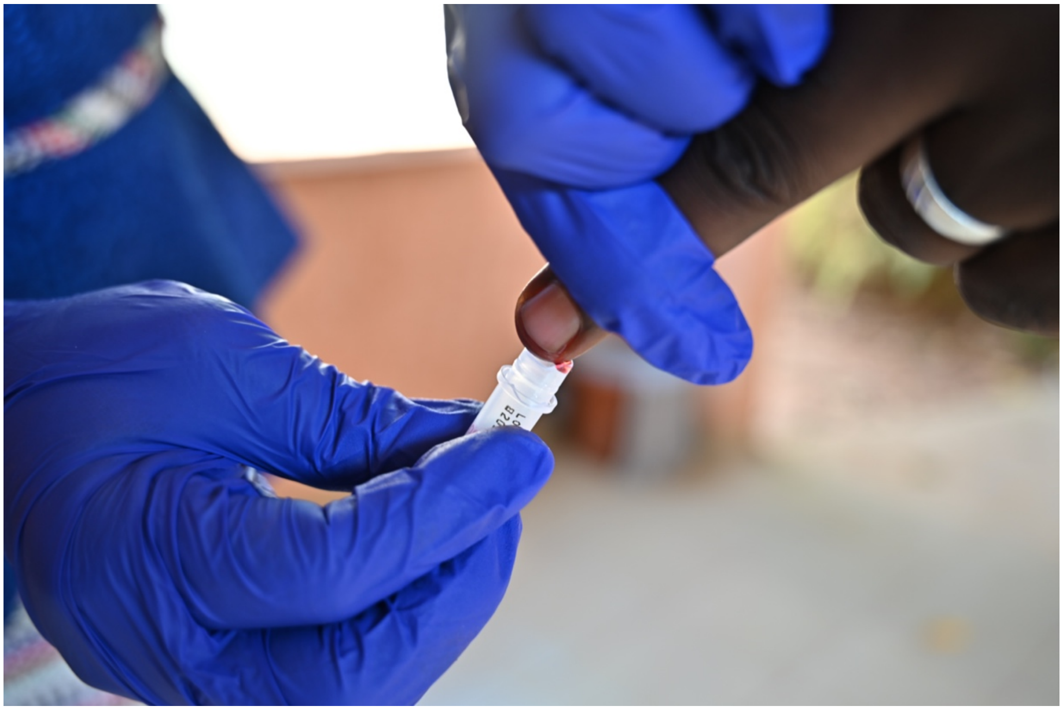

Figure 2: How to position the EDTA violet bd microtainer tube at an angle of 30° to 45° to the finger for blood collection

- Fill the tubes between the fill marks (500µL). An overfilled or underfilled tube may cause clotting and/or cause erroneous test results.
- Replace the cap by turning it and pushing it in.
- Thoroughly homogenize the plasma tube by inverting it 8 to 10 times before continuing with any further collection.
- Once blood collection is complete, apply pressure with a dry cotton ball at the collection site. Then remove the cotton ball and apply a plaster.
- Apply the temporary label "acco_saed_db###m" to the 500µL purple EDTA microtainer tube and place it in the Delta-T® cooler bag (10L).
- Record the date and time of collection on the CAPI (data collection sheet) that corresponds to the visit (delivery).

– Transfer the 500µL purple EDTA microtainer tube contained in the Delta-T® cooler bag (10L) to the laboratory for immunology.

3. Volumetric absorptive microsampling (VAMS) collection

– Turn the participant's hand, the palm side facing up and gently massage the finger so that the blood forms a large drop on the surface of the fingertips.

– Use the neoteryx® microsampler (VAMS) to collect blood by bringing the white tip into contact with the blood drop (Figure 3)

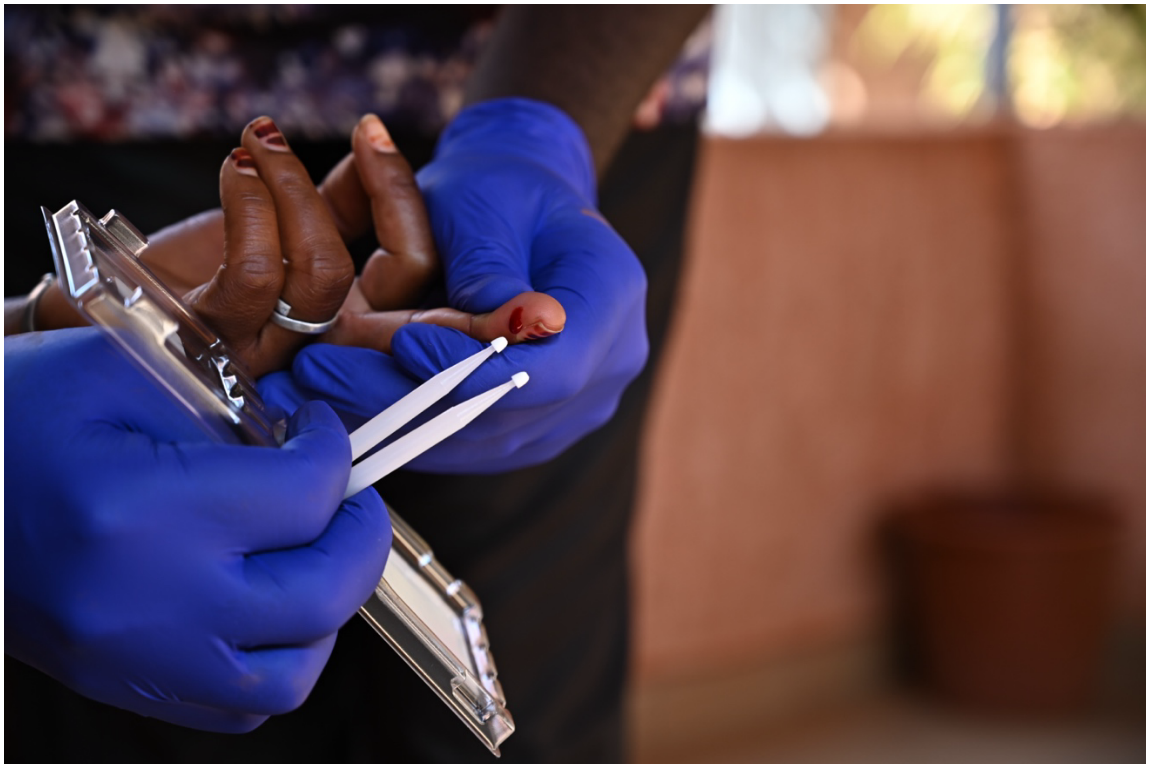

Figure 3: Use of the VAMS microsampler

– Wait patiently for the white tip to turn red and count two seconds before removing it (Figure 4).

– Slowly and gently remove the tip of the neoteryx® microsampler (VAMS).

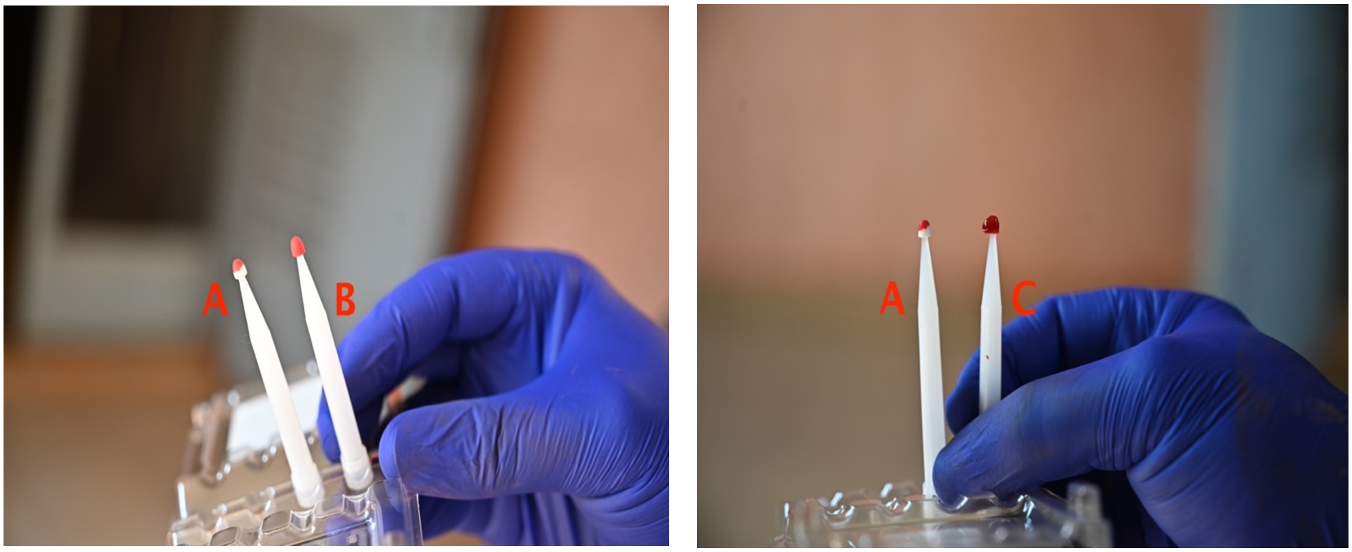

Figure 4 : Importance of the 2 seconds.

A: Less than 2 secondes

B: Correct time

C: More than 2 secondes

- Once blood collection is complete, apply pressure with a dry cotton ball at the collection site. Then remove the cotton ball and apply a rectangular Leukoplast soft plaster (19mm x 72mm).
- Close the VAMS and label.
- Fill in:
    - The CAPI (data collection sheet) that corresponds to the visit (delivery)
    - The VAMS tracking form
- Dispose of all used lancets in the biohazard needle container
- Store the VAMS in their zip bag and transfer them to the laboratory.

4. Samples transport and storage

- All blood samples (i.e., 500-μL microtainer violet EDTA tube, 10-μL VAMS, and 20-μL VAMS) must be transferred to the laboratory within 4 hours after collection.
- The blood samples in the EDTA tubes are dedicated to immunophenotyping.
- The 10μL VAMS and 20μL VAMS samples will be put away in autoracks and immediately stored in the -80°c freezer.

5. Troubleshooting & error and interferences

- Properly affixing and waiting a few seconds for the labels to adhere is crucial to prevent them from coming off the tubes once they are stored in the liquid nitrogen cylinder. Careless application of the labels by team members can cause this issue. To avoid this problem, it is essential to take the time to apply the labels to the entire surface of the tubes before storing them.

Collection of blood samples in neonates: Standard Operating Procedures (SOP) for the DenBalo Study

Lionel Olivier Ouédraogo,[1,2] Trenton Dailey-Chwalibóg,[1] Kim Lagerborg,[3] Lori Glenwinkel,[4] Annie Moradian,[5] Shruti Rao,[5] Moctar Ouédraogo,[6] Anderson Compaoré,[6] Cheick Ahmed Ouattara,[6,7] Carl Lachat,[1] Laéticia Céline Toé,[1,8]

Abstract

Standard operating procedures are crucial to guarantee sample collection consistency and quality during a study. The importance of high-quality biological samples is amplified by researchers' pursuit of a multi-omics approach. Many studies have been carried out in developed countries explaining the procedures used for biological samples collection for multi-omics analysis. However, a little or none of those studies were carried out in Africa where working conditions are totally different and sometimes rudimentary.

This standard operating procedure (SOP) provides a guideline for blood samples collection in neonate enrolled in the study "Description and Comparison of Biological Vulnerability in Small, vulnerable newborns versus Healthy community controls in Urban Burkina Faso (DenBalo) Study: Gut Microbiota, Immune System, and Breastmilk Assembly and Development in the First Days and Weeks of Life" (ONZ-2022-0500, 050-2022/CEIRES du 16 September 2022). Neonates' blood samples are dedicated to immunological analysis (cytokines, chemokines and immunostaining analysis), to mycotoxin profiling and to omics study namely metabolomics and proteomics.

The aim of this SOP is to establish a standardized process for blood samples collection in neonates ensuring the process consistency and the quality control of the samples collected. To that purpose this SOP details steps to follow to guarantee a good sample collection including material and tools preparation, specimen collection, labeling, handling and storage, and the list of required tracking documents to fill out during the process. By following this SOP, healthcare professionals can optimize the quality and reliability of blood samples. Our SOP could be a reference guideline on blood samples collection in Africa specifically tailored for proteomics and metabolomics analysis.

Keywords: Standard operating procedure, blood samples, neonates, proteomics, metabolomics, mycotoxin profiling, immunostaining.

[1] Department of Food Technology, Safety and Health, Faculty of Bioscience Engineering, Ghent University, Ghent, Belgium
[2] Centre Muraz, Bobo-Dioulasso, Burkina Faso
[3] Broad Institute of Harvard and MIT, Cambridge, MA 02142, USA
[4] Department of Biological Sciences, Columbia University, Howard Hughes Medical Institute, New York, United States.
[5] Precision Biomarker Laboratories, Beverly Hills, CA 90211, USA
[6] Agence de Formation de Recherche et d'Expertise en Santé pour l'Afrique (AFRICSanté)
[7] École Doctorale de Santé Publique, Université Nazi Boni, Bobo-Dioulasso, Burkina Faso
[8] Unité Nutrition et Maladies Métaboliques, Institut de Recherche en Sciences de la Santé (IRSS)

NOTES

- The blood sample is collected at the heel.
- The lancets to use depend on the weight of the baby.
- For babies weighing less than 2kg use the pink Quikheel Preemie Lancet
- For babies weighing more than 2kg use the green Quikheel Lancet
- If there is not sufficient volume of blood at the same site to collect both VAMS and the EDTA tube, perform a second attempt at the opposite foot. Do not exceed two attempts.
- The choice of the puncture site is crucial:
- It is very important, when choosing the puncture site, to eliminate any risk of touching the heel bone.
- DO NOT PRICK the posterior curve of the heel.
- DO NOT PRICK the central part of the heel because of the risk of nerve, tendon and cartilage damage.
- DO NOT PRICK any part of the foot other than the recommended heel area.
- DO NOT PRICK a previously used puncture site. It may be infected.
- VAMS collection follows plasma collection if the blood is not clotted and continues to flow in sufficient quantity after plasma collection. If not, apply the plaster at the puncture site and locate another puncture site on the opposite heel and repeat all steps of the general capillary collection method.
- Always begin collection of VAMS with 20µL VAMS and end with 10µL VAMS

MATERIALS

MATERIALS	QUANTITIES
Pen	01
Soap	01
Romed® Alcohol Pad	At least 06 pads
Emla cream 5% ® Anesthesia cream	01 tube
Cotton ball	As many as needed
500-µl violet BD microtainer EDTA tube	01
Pink Quikheel Preemie Lancet or Green Quikheel Lancet	02
Delta-T® cooler bag (10L)	01
Rack	01
Neoteryx® microsampler (VAMS) 10µl	02
Neoteryx® microsampler (VAMS) 20µl	02
Leukoplast soft plaster (22mm x 22mm).	02
Biohazard needle container	01
Cryolabels	01 sheet
Gloves	02 pairs
Samsung Tablet	01
Biohazard Bag	01

PROCEDURE

1. General method for capillary sampling

- The health worker should wash their hands thoroughly with soap and water.
- Select a non-calloused area at the heel (Figure 1)

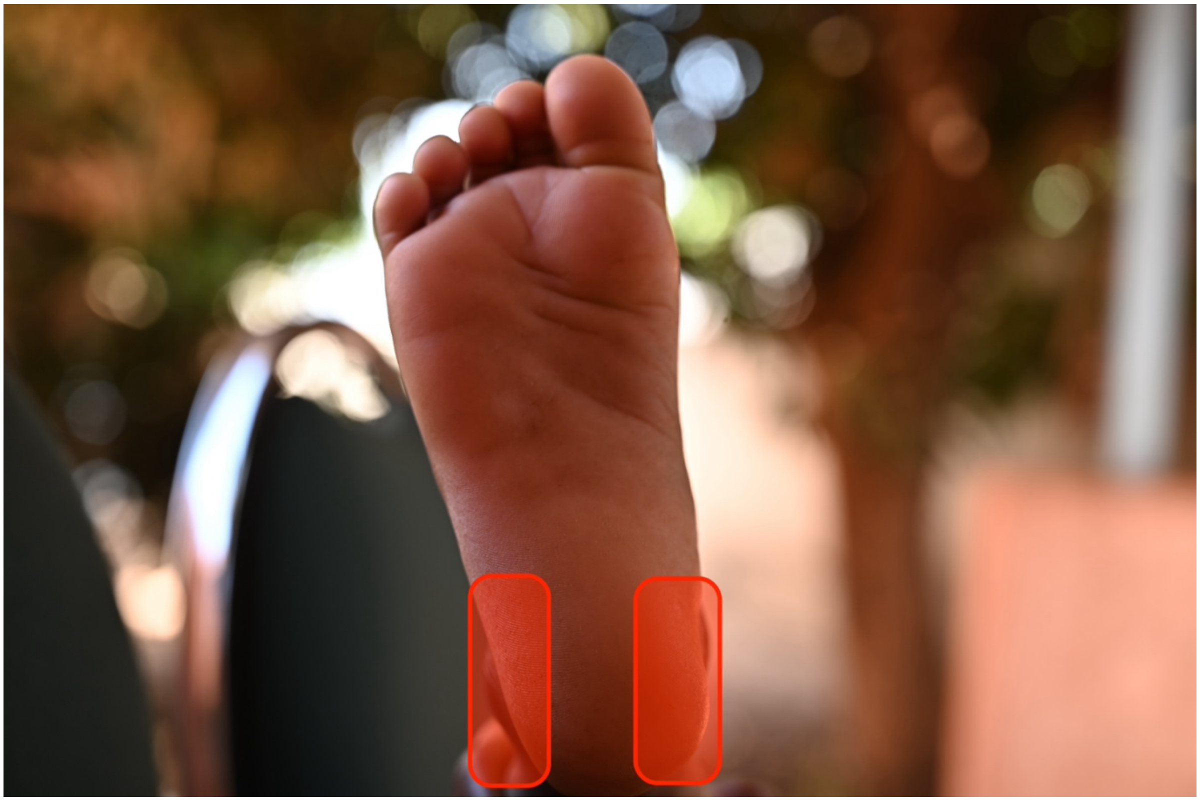

Figure 1 : Possible sampling areas on the heel

- Clean the site with a ROMED® ALCOHOL PAD and let it dry completely.
- Apply a thin layer of EMLA CREAM 5% ® ANESTHESIA CREAM at least 15 minutes prior to sampling on the area selected for collection.
- Clean the site with a ROMED® ALCOHOL PAD and let it dry completely.
- Place the lancet firmly on the puncture site and prick (Figure 1 and Figure 2). To facilitate the process, gently massage the sole of the foot. Do not remove the lancet until you hear an audible click.

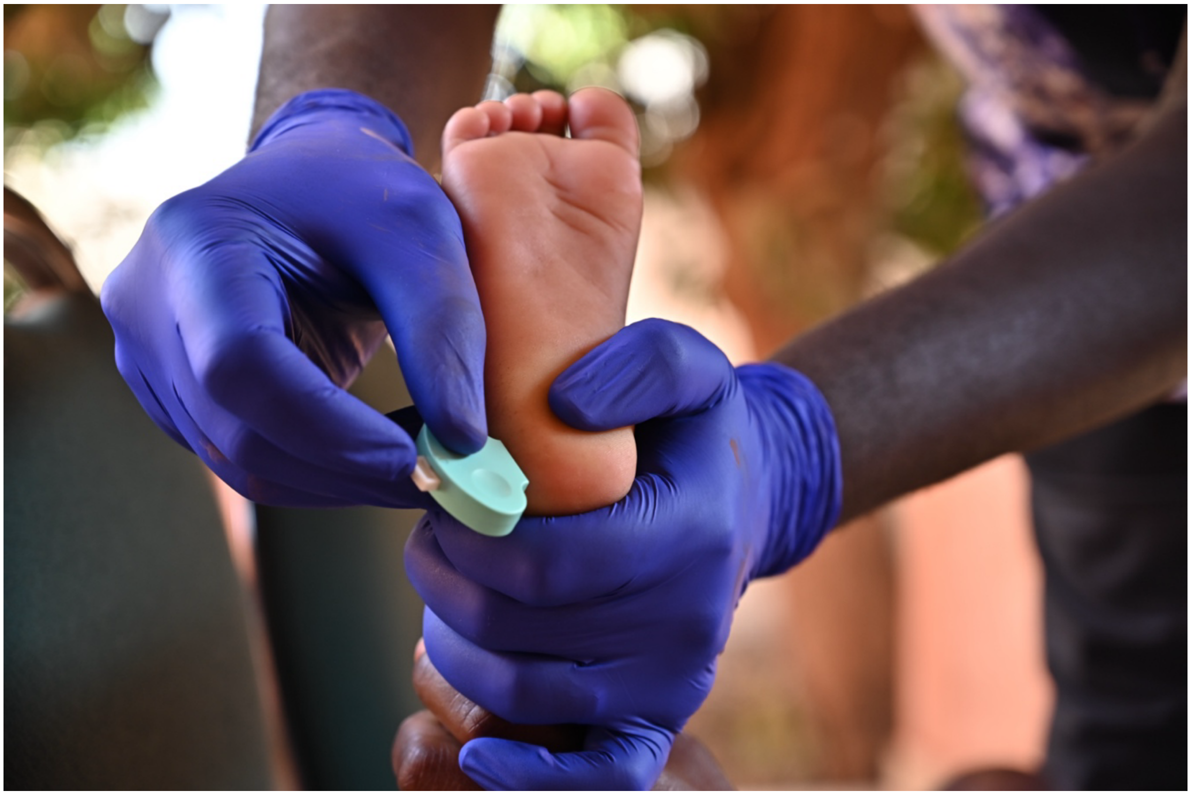

Figure 2 : Prick process

- Wipe off the first drop of blood with a cotton ball.

2. Plasma sample collection

- Position the newborn's heel the sole of the foot facing the ground.
- Hold the 500-µl violet BD microtainer EDTA tube at a 30° to 45° angle to the surface of the puncture site (Figure 3). Touch the collection end of the tube to drop the blood. Avoid scratching the skin surface to collect the blood sample. After collecting 2 or 3 drops, the blood will flow freely down the wall of the tube to the bottom.

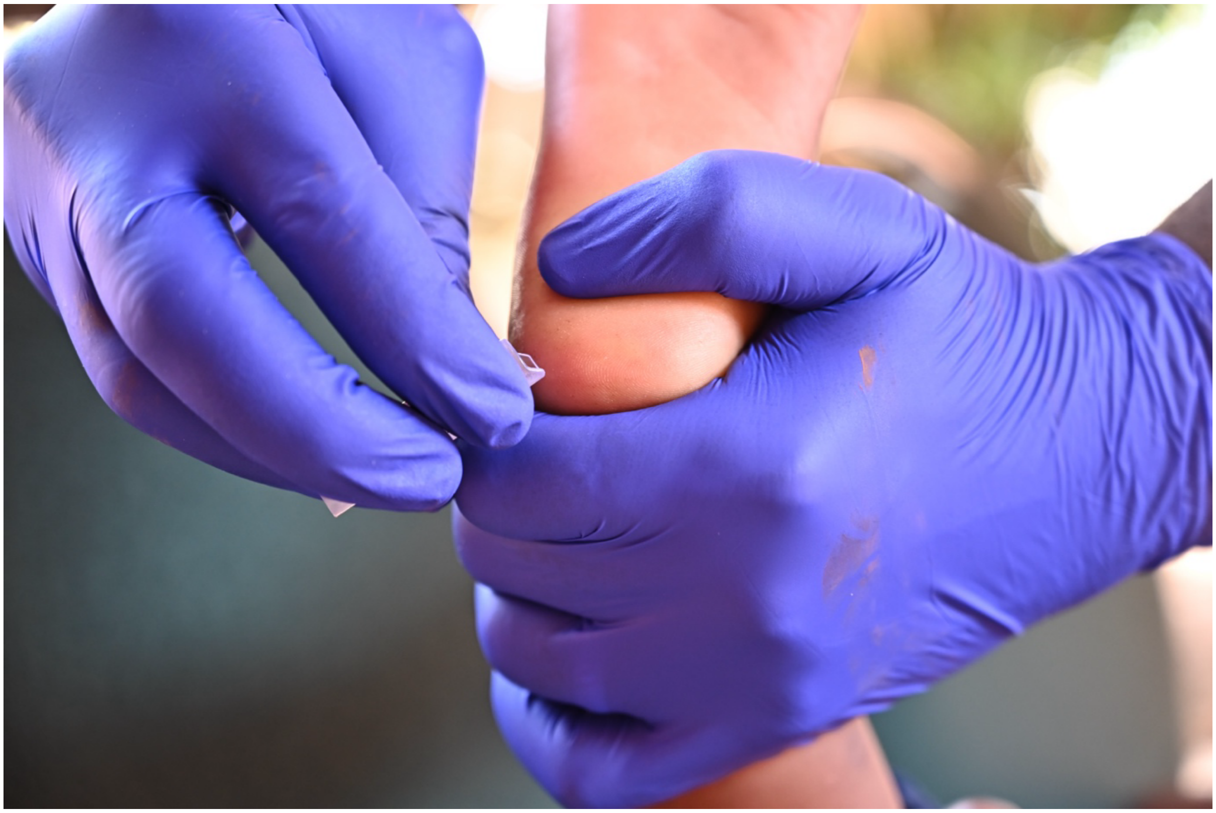

Figure 3: How to position the EDTA violet bd microtainer tube at an angle of 30° to 45° to the heel for blood collection

- Fill the tubes between the fill marks (500µL). An overfilled or underfilled tube may cause clotting and/or cause erroneous test results.
- Replace the cap by turning it and pushing it in.
- Thoroughly homogenize the plasma tube by inverting it 8 to 10 times before continuing with any further collection.
- Once blood collection is complete, apply pressure with a dry cotton ball at the collection site. Then remove the cotton ball and apply a plaster.
- Apply the temporary label "acco_saed_db###e" to the 500µL microtainer purple EDTA microtainer tube and place it in the Delta-T® cooler bag (10L) on a rack.

- Record the date and time of collection on the CAPI (data collection sheet) that corresponds to the visit (Day 1, Day 3, Day 5, Day 7, Day 28, Day 60).
- Transfer the 500µL purple EDTA microtainer tube contained in the Delta-T® cooler bag (10L) to the laboratory for immunology.

3. Volumetric absorptive microsampling (VAMS) collection

- Use the neoteryx® microsampler (VAMS) to collect blood by bringing the white tip into contact with the blood drop (Figure 4)

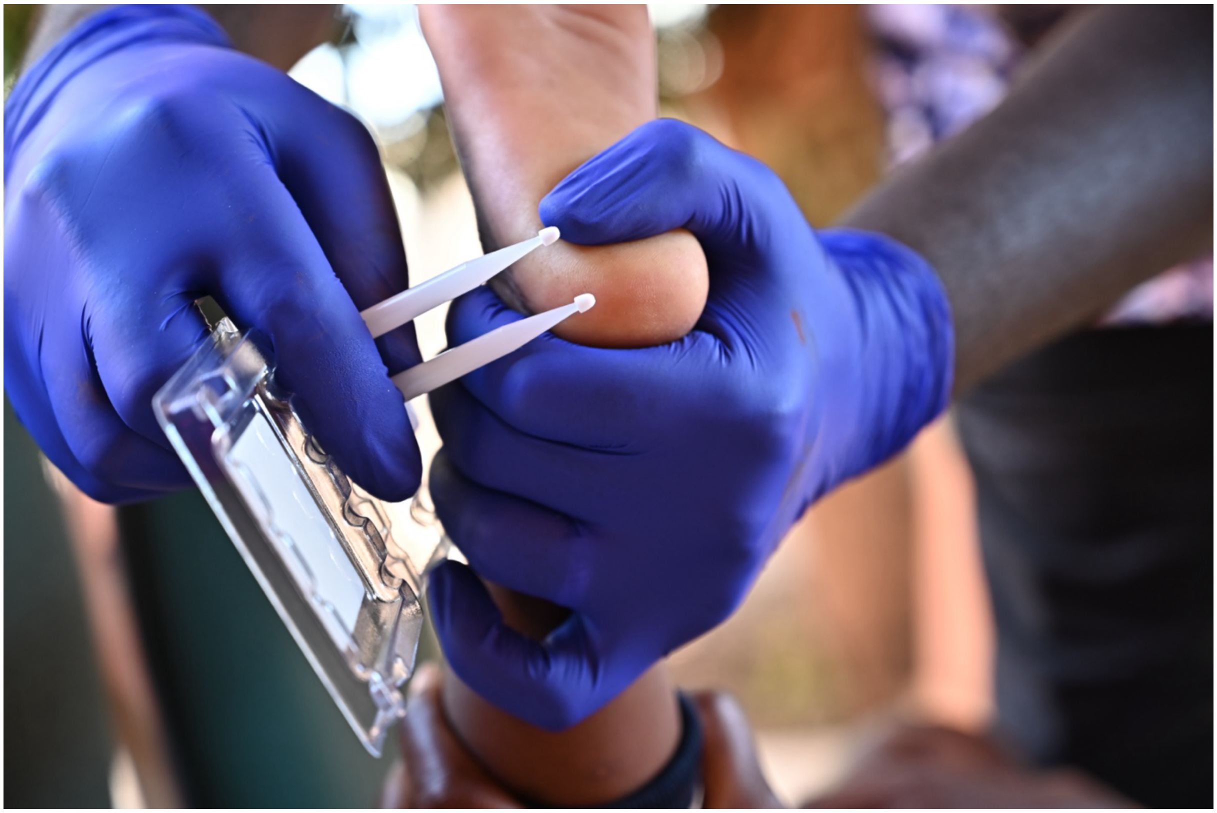

Figure 4: Use of the VAMS microsampler

- Wait patiently for the white tip to turn red and count two seconds before removing it (Figure 5).
- Slowly and gently remove the tip of the neoteryx® microsampler (VAMS).

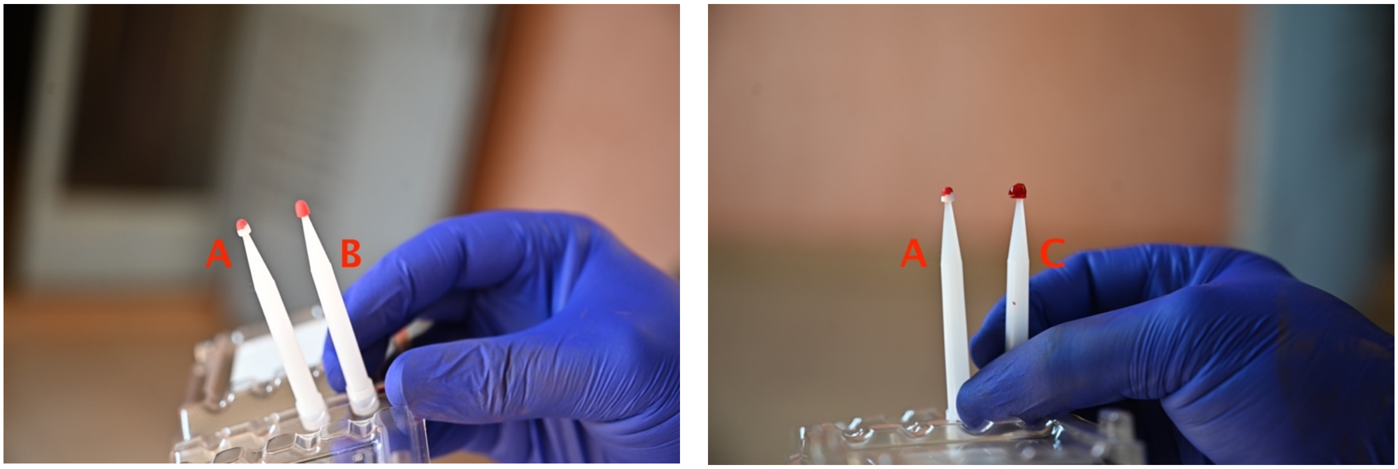

Figure 4 : Importance of the 2 seconds.

A: Less than 2 secondes

B: Correct time

C: More than 2 secondes

- Once blood collection is complete, apply pressure with a dry cotton ball at the collection site. Then remove the cotton ball and apply a rectangular Leukoplast soft plaster (22mm x 22mm).
- Close the VAMS and label.
- Fill in:
    - The CAPI (data collection sheet) that corresponds to the visit
    - The VAMS tracking form
- Dispose of all used lancets in the biohazard needle container
- Store the VAMS in their zip bag and transfer them to the laboratory

4. Samples transport and storage

- All blood samples (i.e., 500-µL microtainer violet EDTA tube, 10-µL VAMS, and 20-µL VAMS) must be transferred to the laboratory within 4 hours after collection.
- The blood samples in the EDTA tubes are dedicated to immunophenotyping.
- The 10µL VAMS and 20µL VAMS samples will be put away in autoracks and immediately stored in the -80°c freezer.

5. Troubleshooting & error and interferences

- Some participants may experience quick blood clotting before the required samples can be collected. If this happens, it is important not to prick the same collection site again. Instead, choose a different collection site on the opposite heel from the first one, or reschedule another blood collection appointment. This approach can help to ensure that a sufficient sample is collected without causing discomfort or harm to the participant.
- Properly affixing and waiting a few seconds for the labels to adhere is crucial to prevent them from coming off the tubes once they are stored in the liquid nitrogen cylinder. Careless application of the labels by team members can cause this issue. To avoid this problem, it is essential to take the time to apply the labels to the entire surface of the tubes before storing them.

Collection of stool samples in mothers: Standard Operating Procedures (SOP) for the DenBalo Study

Lionel Olivier Ouédraogo,[1,2] Trenton Dailey-Chwalibóg,[1] Thy Nguyen,[3] Justin Sonnenburg,[3] Erica Sonnenburg,[3] Moctar Ouédraogo,[4] Anderson Compaoré,[4] Cheick Ahmed Ouattara,[4,5] Carl Lachat,[1] Laéticia Céline Toé,[1,6]

Abstract

Standard operating procedures are crucial to guarantee sample collection consistency and quality during a study. The quality of biological samples is all the more important when considering the high degree of sensitivity of multi-omics approaches. Many studies have been carried out in developed countries explaining the procedures used for biological samples collection for multi-omics analysis. However, limited data exists for studies carried out in Africa where working conditions differ substantially in access to reagents, supplies, and equipment that are standard in high-income countries.

This standard operating procedure (SOP) provides a guideline for stool sample collection in mothers enrolled in the study "Description and Comparison of Biological Vulnerability in Small, vulnerable newborns versus Healthy community controls in Urban Burkina Faso (DenBalo) Study: Gut Microbiota, Immune System, and Breastmilk Assembly and Development in the First Days and Weeks of Life" (ONZ-2022-0500, 050-2022/CEIRES du 16 September 2022). Mothers' stool samples are dedicated for enteropathogen detection and multi-omic microbiome characterization that includes metagenomics, metabolomics, and meta-proteomics. Enteropathogen detection will be carried out by using the TaqMan Array Card system, a 384-well singleplex real-time PCR format used to detect 62 infection targets including viruses, bacteria, protozoa and helminths. Microbiome multi-omics will characterize gut microbial diversity, composition, and functions, small molecules, and human gut protein responses using statistical and machine learning approaches.

The aim of this SOP is to establish a standardized process for stool samples collection in mothers ensuring process consistency and quality of the samples collected. To that purpose, this SOP details material and tools preparation, specimen collection, labeling, handling and storage, and the list of required tracking documents to fill out during the process. By following this SOP, healthcare professionals can optimize the quality and reliability of stool samples collected. Our SOP could be a reference guideline on stool samples collection in Africa specifically tailored for proteomics and metabolomics analysis.

Keywords: Standard operating procedure, stool samples, mothers, metagenomics, metabolomics

[1] Department of Food Technology, Safety and Health, Faculty of Bioscience Engineering, Ghent University, Ghent, Belgium
[2] Centre Muraz, Bobo-Dioulasso, Burkina Faso
[3] Department of Microbiology and Immunology Stanford University School of Medicine
[4] Agence de Formation de Recherche et d'Expertise en Santé pour l'Afrique (AFRICSanté)
[5] École Doctorale de Santé Publique, Université Nazi Boni, Bobo-Dioulasso, Burkina Faso
[6] Unité Nutrition et Maladies Métaboliques, Institut de Recherche en Sciences de la Santé (IRSS)

Notes

- Please collect only stool samples, ensure there is no mixture with urine. Kindly instruct the participant to empty her bladder before having a bowel movement.
- Stool samples must be collected, aliquoted and stored in the Delta -T® cooler bag (10L) within 45 minutes after defecation.
- It is preferable to collect all stool samples from the same stool sample. However, if the provided stool is insufficient for collecting all the planned aliquots, please reschedule another visit for collection within a maximum of 7 days.
- The following aliquots should be made:
    - Stool for genomics
    - Stool for proteomics
    - Stool for metabolomics
    - Stool for TAC
- The number of 2 ml bar-coded cryotubes required for stool sample collection varies from one visit to another. The following quantities are required:
    - 4 x 2 ml bar-coded cryotubes for the sonogram visit (24 weeks and 29 weeks and 6 days)
    - 4 x 2 ml bar-coded cryotubes for the home visit (33-34 weeks)
    - 4 x 2 ml bar-coded cryotubes for the day 30 visit
    - 4 x 2 ml bar-coded cryotubes for the day 180 visit
    - 3 x 2 ml bar-coded cryotubes for the day 7 and day 60 visits.
- The same spoon attached to the lid of the sterile stool container can be used to perform the different aliquots if there is no suspicion of contamination.

MATERIALS

MATERIALS	QUANTITIES
Soap	01
Pen	01
Biohazard needle container	01
Cryolabels	04
Gloves*	01 pair
Samsung Tablet	01
Delta-T® cooler bag (10L) containing icepacks	01
Rack	01
Bristol scale	01
2ml Bar-coded cryotube *	04
Sterile Stool Container Faeces Collection with Spoon *	01
Biohazard Bag	01

Note: As a precautionary measure against potential contamination, please plan an additional quantity for items marked with an asterisk (*)

PROCEDURE

1. Stool sample

- The day before the stool sample collection, the health worker should inform the participant about her scheduled appointment for sample collection.
- On the morning of the scheduled visit provide the participant with a sterile stool container faeces collection with spoon. Explain to her to collect her stool inside the provided container and then return it to the health worker.
- The health worker must wear gloves after properly washing his hands with soap and water.
- After retrieving the stool sample the health worker must follow these steps:
- Thoroughly examine the collected sample for the presence of blood and/or mucus.

- Assess the collected specimen and assign it a grade based on the visual bristol scale.
- Use the spoon attached to the lid of the sterile stool container, to carefully homogenize the stool sample ensuring that the solid and liquid components are well mixed.
- With the same spoon attached to the lid of the sterile stool container, transfer the stool into 2 ml bar-coded graduated cryotube. The 2 ml bar-coded cryotube should contain no more than 1.8ml of stool.
    - Note 1: Remember to label the 2 ml bar-coded cryotubes before aliquoting the stool samples.
    - Note 2: The number of 2 ml bar-coded cryotubes required for stool sample collection varies from one visit to another.
- Close the 2mL bar-coded cryotube tightly and ensure that there are no leaks.
- Put the 2 mL bar-coded cryotubes on a rack and place in the Delta-T® Cooler Bag (10L).
- Dispose of all biomedical waste in the Biohazard Bag.
- Complete the documentation which includes:
    - filling out the appropriate CAPI (data collection sheet) corresponding to the visit type (i.e., sonogram, home visit, day 7, day 30, day 60 or day 180)
    - completing the cryotube tracking form and the transfer to portable storage liquid nitrogen tank form

2. Samples transport and storage

- Transfer the collected cryotubes (contained in the Delta-T® cooler bag (10L)) to the portable storage liquid nitrogen tank located at the health center. Cryotubes should be stored in the liquid nitrogen tank within 2 hours of defecation.

- Once the liquid nitrogen tank is filled with stool cryotubes, transfer the liquid nitrogen tank to the laboratory for further storage of samples in the -80°c freezer.

3. Troubleshooting & error and interferences

- If a participant does not have a bowel movement during the scheduled collection time, it may be because they had a bowel movement shortly before. In such cases, the team staff must reschedule another visit to collect the samples. This approach can help ensure the collection of a sufficient and accurate sample.
- Properly affixing and waiting a few seconds for the labels to adhere is crucial to prevent them from coming off the tubes once they are stored in the liquid nitrogen cylinder. Careless application of the labels by team members can cause this issue. To avoid this problem, it is essential to take the time to apply the labels to the entire surface of the tubes before storing them.
- If the stool is contaminated with urine, kindly request the participant to recollect the stool. If it is not possible for her to have a bowel movement on the same day, please promptly reschedule another appointment to ensure proper collection of the sample.
- In case of insufficient quantity of stool sample please promptly reschedule another appointment with the participant to ensure an adequate sample collection.
- If there is any issue with the storage conditions, please promptly report it in the CAPI (data collection sheet).
- To secure the labels on the cryotubes and prevent them from detaching, we recommend applying two complete turns of tape around the tube. This ensures the labels remain securely attached during storage and handling.

Collection of stool samples in neonates: Standard Operating Procedures (SOP) for the DenBalo Study

Lionel Olivier Ouédraogo,[1,2] Trenton Dailey-Chwalibóg,[1] Thy Nguyen,[3] Justin Sonnenburg,3 Erica Sonnenburg,[3] Moctar Ouédraogo,[4] Anderson Compaoré,[4] Cheick Ahmed Ouattara,[4] Carl Lachat,[1] Laéticia Céline Toé,[1,5]

Abstract

Standard operating procedures are crucial to guarantee sample collection consistency and quality during a study. The quality of biological samples is all the more important when considering the high degree of sensitivity of multi-omics approaches. Many studies have been carried out in developed countries explaining the procedures used for biological samples collection for multi-omics analysis. However, limited data exists for studies carried out in Africa where working conditions differ substantially in access to reagents, supplies, and equipment that are standard in high-income countries.

This standard operating procedure (SOP) provides a guideline for stool sample collection in neonates enrolled in the study "Description and Comparison of Biological Vulnerability in Small, vulnerable newborns versus Healthy community controls in Urban Burkina Faso (DenBalo) Study: Gut Microbiota, Immune System, and Breastmilk Assembly and Development in the First Days and Weeks of Life" (ONZ-2022-0500, 050-2022/CEIRES du 16 September 2022). Neonates' stool samples are dedicated for enteropathogen detection and multi-omic microbiome characterization that includes metagenomics, metabolomics, and meta-proteomics. Enteropathogen detection will be carried out by using the TaqMan Array Card system, a 384-well singleplex real-time PCR format used to detect 62 infection targets including viruses, bacteria, protozoa and helminths. Microbiome multi-omics will characterize gut microbial diversity, composition, and functions, small molecules, and human gut protein responses using statistical and machine learning approaches.

The aim of this SOP is to establish a standardized process for stool samples collection in neonates ensuring process consistency and quality of the samples collected. To that purpose, this SOP details material and tools preparation, specimen collection, labeling, handling and storage, and the list of required tracking documents to fill out during the process. By following this SOP, healthcare professionals can optimize the quality and reliability of stool samples collected. Our SOP could be a reference guideline on stool samples collection in Africa specifically tailored for proteomics and metabolomics analysis.

Keywords: Standard operating procedure, stool samples, neonates, metagenomics, metabolomics

[1] Department of Food Technology, Safety and Health, Faculty of Bioscience Engineering, Ghent University, Ghent, Belgium
[2] Centre Muraz, Bobo-Dioulasso, Burkina Faso
[3] Department of Microbiology and Immunology Stanford University School of Medicine
[4] Agence de Formation de Recherche et d'Expertise en Santé pour l'Afrique (AFRICSanté)
[5] Unité Nutrition et Maladies Métaboliques, Institut de Recherche en Sciences de la Santé (IRSS)

Notes

- Please collect only stool samples, ensure there is no mixture with urine. To ensure that only stool is collected and to prevent any contamination from the child's urine, please utilize a boy/girl Urinocol® adhesive bag. This will help maintain the integrity of the stool sample.
- Stool samples should be collected, stored in the Delta -T® cooler bag (10L) within 45 minutes after defecation.
- It is preferable that all stool sample aliquots from one individual at a specific timepoint are collected from the same defecation/stool sample. However, if the provided stool is insufficient for collecting all the planned aliquots, please reschedule another visit for collection as soon as possible and in accordance with the study design and practical constraints. .
- The following aliquots should be made:
  - Stool for genomics
  - Stool for proteomics
  - Stool for metabolomics
  - Stool for TAC
- The number of 2 ml bar-coded cryotubes required for stool sample collection varies from one visit to another. The following quantities are required:
  - 3 x 2 ml bar-coded cryotubes on the delivery day and for the day 1, day 2, day 3, day 4, day 5, day 6, day 7, day 14 and day 60 visits
  - 4 x 2 ml bar-coded cryotubes for the day 30 and day 180 visits
- The same spoon attached to the lid of the sterile stool container can be used to perform the different aliquots if there is no suspicion of contamination.

MATERIALS

MATERIALS	QUANTITIES
Soap	01
Changing pad	01
Romed® Alcohol Pad*	At least 06
Sterile protection sheet*	01
Pen	01
Biohazard needle container	01
Cryolabels	04
Gloves*	01 pair
Samsung Tablet	01
Delta-T® cooler bag (10L) containing icepacks	01
Rack	01
Bristol scale	01
Sterile Stool Container Faeces Collection with Spoon*	01
Urinocol boy/girl pouch*	01
2ml Bar-coded cryotube*	04
Biohazard Bag	01

Note: As a precautionary measure against potential contamination, please plan an additional quantity for items marked with an asterisk (*)

PROCEDURE

1. Stool samples collection.

- Before initiating the stool sample collection, the health worker should begin by clearly explaining to the mother the purpose and procedure involved in collecting the stool sample.
- The health worker must wear gloves after properly washing his hands with soap and water.
- Ensure thorough sterilization of the changing pad by using alcohol swabs. Use a minimum of three alcohol wipes to achieve proper sterilization.
- Gently lay the child on the changing pad. Ask the mother to clean up and down the child's pubic and genital area with wet wipes (Pampers Wipes). To prevent contamination by urine, securely

attach an adhesive Urinocol® boy pouch (♂)/ adhesive Urinocol® girl pouch (♀) at the child's vulva or penis and scrotum.

- Use a sterile protection sheet and fit it like a diaper around the newborn. The white plastic side of the protection sheet should be in contact with the newborn's skin. Wait patiently for the newborn to have a bowel movement (it could sometimes take 2 or 3 hours).
- After the bowel movement, remove the sterile protection sheet from the newborn and then the urine collection bag.
- Transfer the stool collected into the Sterile Stool Container Faeces Collection with Spoon and perform the following steps:
    - Label the 2ml bar-coded cryotubes before aliquoting the stool samples.
    - Check the collected sample for blood and/or mucus.
    - Assess the collected specimen and grade it based on the visual Bristol scale.
    - Use the spoon attached to the lid of the sterile stool container, to carefully homogenize the stool sample ensuring that the solid and liquid components are well mixed.
    - With the same spoon attached to the lid of the sterile stool container, transfer the stool into 2 ml bar-coded graduated cryotube. The 2 ml bar-coded cryotube should contain no more than 1.8ml of stool.
    - Note 1: Remember to label the 2 ml bar-coded cryotubes before aliquoting the stool samples.
    - Note 2: The number of 2 ml bar-coded cryotubes required for stool sample collection varies from one visit to another.

- Close the 2mL bar-coded cryotube tightly and ensure that there are no leaks. You should have 3 aliquots (stools for genomics, metabolomics and proteomics) on days 1, 2, 3, 4, 5, 6, 7, 14, 60 and 4 aliquots (stools for genomics, metabolomics, proteomics and TAC) on days 30 and 180.
- Put the 2 mL bar-coded cryotubes on a rack and place in the Delta-T® Cooler Bag (10L).
- Dispose of all biomedical waste in the Biohazard Bag.
- Complete the documentation which includes:
    - filling out the appropriate CAPI (data collection sheet) corresponding to the visit type (i.e., delivery day, day 1, day 2, day 3, day 4, day 5, day 6, day 7, day 14, day 30, day 60, day 180).
    - completing the cryotube tracking form and the transfer to portable storage liquid nitrogen tank form.

2. Samples transport and storage

- Transfer the collected cryotubes (contained in the Delta-T® cooler bag (10L)) to the portable storage liquid nitrogen tank located at the health center. Cryotubes should be stored in the liquid nitrogen tank within 2 hours of defecation.
- Once the liquid nitrogen tank is filled with stool cryotubes, transfer the liquid nitrogen tank to the laboratory for further storage of samples in the -80°c freezer.

3. Troubleshooting, errors, and interferences

- If a neonate does not have a bowel movement during the scheduled collection time, it could be due to a recent bowel movement. In such cases, the team staff can ask the mother to breastfeed the baby and wait for another bowel movement before proceeding with the collection. This approach ensures the collection of a sufficient and accurate sample.

- If there is evidence of urine in the stool sample, it may indicate a potential issue with the placement or functionality of the urinocol. To ensure the accuracy of stool sample collection, it is crucial to address this matter before continuing. This can be achieved by either replacing the urinocol or adjusting its placement to prevent any further contamination of the stool samples.
- In case of insufficient quantity of stool sample please promptly reschedule another appointment with the participant’s parents to ensure an adequate sample collection
- If there is any issue with the storage conditions, please promptly report it in the CAPI (data collection sheet).
- To secure the labels on the cryotubes and prevent them from detaching, we recommend applying two complete turns of tape around the tube. This ensures the labels remain securely attached during storage and handling.

Collection of breastmilk samples: Standard Operating Procedures (SOP) for the DenBalo Study

Lionel Olivier Ouédraogo,[1,2] Trenton Dailey-Chwalibóg,[1] Kelsey Fehr,[3] Natalie Rodriguez,[3] Meghan B. Azad,[3] Moctar Ouédraogo,[4] Anderson Compaoré,[4] Cheick Ahmed Ouattara,[4,5] Carl Lachat,[1] Laéticia Céline Toé,[1,6]

Abstract

Standard operating procedures are crucial to guarantee sample collection consistency and quality during a study. The quality of biological samples is all the more important that researchers aim to have a multi-omics approach. Many studies have been carried out in developed countries explaining the procedures used for biological samples collection for multi-omics analysis (Koh and al., 2022; Zreloff and al., 2023; Zubeldia-Varela and al., 2020). However, a little or none of those studies were carried out in Africa where working conditions are totally different and sometimes rudimentary.

This standard operating procedure (SOP) provides a guideline for breastmilk samples collection in mothers enrolled in the study "Description and Comparison of Biological Vulnerability in Small, vulnerable newborns versus Healthy community controls in Urban Burkina Faso (DenBalo) Study: Gut Microbiota, Immune System, and Breastmilk Assembly and Development in the First Days and Weeks of Life" (ONZ-2022-0500, 050-2022/CEIRES du 16 September 2022). Breastmilk samples will be distributed to multiple laboratories for analysis of macronutrients, micronutrients, oligosaccharides, growth factors, immunoglobulins, cytokines, metabolites and microbes.

The aim of this SOP is to establish a standardized process for breastmilk samples collection ensuring the process consistency of the samples collected. To that purpose this SOP details steps to follow to undertake a good sample collection including material and tools preparation, specimen collection, labeling, handling and storage, and the list of required tracking documents to fill out during the process. By following this SOP, healthcare professionals can optimize the quality and reliability of breastmilk samples collected. Our SOP could be a reference guideline on breastmilk samples collection in Africa specifically in nursing mothers during neonates ' early weeks of life.

Keywords: Standard Operating Procedure (SOP), breastmilk sample, macronutrients, micronutrients, oligosaccharides, growth factors, immunoglobulins, cytokines, metabolites, microbes, early weeks of life

---

[1] Department of Food Technology, Safety and Health, Faculty of Bioscience Engineering, Ghent University, Ghent, Belgium
[2] Centre Muraz, Bobo-Dioulasso, Burkina Faso
[3] Manitoba Interdisciplinary Lactation Centre (MILC), Department of Pediatrics and Child Health, University of Manitoba: Winnipeg, Manitoba, CA
[4] Agence de Formation de Recherche et d'Expertise en Santé pour l'Afrique (AFRICSanté)
[5] École Doctorale de Santé Publique, Université Nazi Boni, Bobo-Dioulasso, Burkina Faso
[6] Unité Nutrition et Maladies Métaboliques, Institut de Recherche en Sciences de la Santé (IRSS)

Notes

– Using the appliance

  - Note 1: Only place items in the sterilizer which are suitable for sterilizing. Do not sterilize items that are filled with liquid, e.g. a teether with cooling fluid.
  - Note 2: Before you sterilize baby bottles and other items, clean them first.
  - Note 3: Wash your hands before starting sterilization.

– Kit elements sterilization: no need to sterilize the kit bottles and the membrane.

MATERIALS

MATERIALS	QUANTITIES
Philips Avent Sterilizer SCF 285 or SCF 284	01
Symphony® Breast Pump Kit	01
Symphony® breast pump	01
Benzalkonium Chloride (BDZ) wipes	04
Plastic bottle	02
Baby bottle	01
Aluminum foil	01
2ml bar-coded sterile Cryotubes	06
Cryolabels	01 sheet
Rack	01
Gloves	02 pairs
Pen	01
Finntip™ Flex 1 - 200 µl pipet	01
Delta-T® cooler bag (10L)	01
Biohazard Bag	01

PROCEDURE

1. Sterilizer components

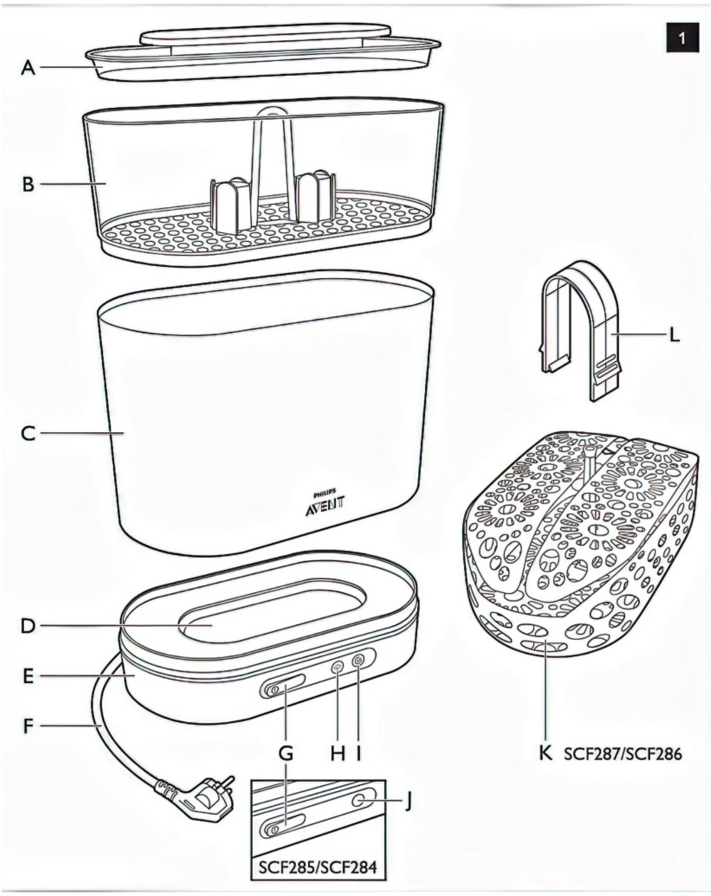

A Lid with grip
B Small basket
C Large basket
D Heating element with water reservoir
E Base
F Mains cord
G Power-on button
H SCF287/SCF286:Sterilising light
I SCF287/SCF286: Ready light
J SCF285/SCF284:Sterilising light
K Dishwasher basket (SCF287/SCF286 only)
L Tongs

Sterilizer component

Source: Philips AVENT SCF284, SCF285, SCF286, SCF287 Manual www.philips.com/welcome

Cautions

- The Electric Steam Sterilizer contains no serviceable parts. Do not attempt to open or repair it
- The appliance becomes extremely hot during sterilization and may cause burns if it is touched.
- Beware of hot steam coming out of the lid or when you remove the lid. It can cause burns.
- The base, baskets and lid become very hot during sterilization; wait a moment after sterilization before touching them. Always use the handle to lift the lid.
- Never move or open the appliance when it is in use or when the water in is still hot.

- Never place items on top of the appliance when it is in use.
- Only use water without any additives.
- Do not put bleach or other chemicals in the appliance.
- Only sterilize baby bottles and other items that are suitable for sterilizers.
- If you want to stop the sterilization process, unplug the appliance or press the power-on button.
- Do not expose the appliance to extreme heat or direct sunlight.
- Always let the appliance cool down before you move or store it.
- Do not sterilize very small items which can fall through the holes in the bottom of the basket.
- Never place items directly on the heating element when the appliance is switched on.
- Always place and use the appliance on a dry, stable, level and horizontal surface.
- Do not place the appliance on a hot surface.
- Always pour any remaining water out of the appliance after use and when the appliance has cooled down.
- Always disconnect the device from supply if it’s left unattended.

Before using for the first time

When you use the appliance for the first time, we advise you to let it complete one sterilization process with empty baskets.

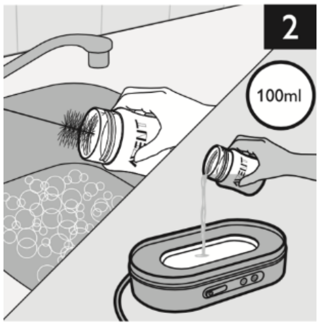

Use for example a clean baby bottle and pour exactly 100ml tap water directly into the water reservoir of the base (Figure 2).

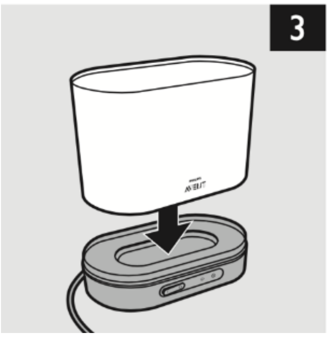

Place the large basket on the base (Figure 3).

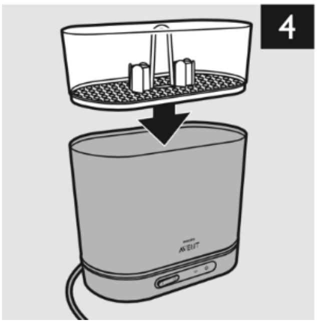

Place the small basket on the large basket (Figure 4)

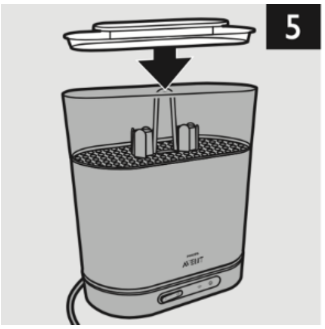

Place the lid on top of the small basket (Figure 5).

Put the plug in the wall socket.

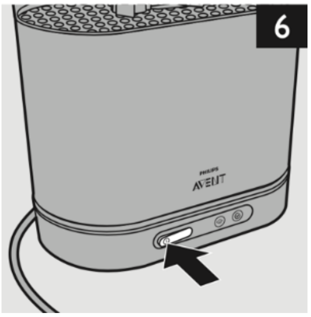

Press the power-on button to switch on the appliance (Figure 6). The sterilising light lights up to indicate that the appliance starts operating. When the sterilization process is finished, the appliance switches off automatically for the SCF285/SCF284 sterilizer type and after 3 minutes for the SCF287/SCF286 type.

After the appliance has cooled down completely, remove the lid, the small basket and the large basket and wipe them dry.

Let the appliance cool down completely for approx. 10 minutes before you use it again.

Note 1: Heating up takes approx. 4 minutes and sterilizing takes approx. 6 minutes.

Note 2: If you plug in the appliance after you have pressed the power-on button, the sterilizer starts to heat up immediately.

Source: Philips AVENT SCF284, SCF285, SCF286, SCF287 Manual www.philips.com/welcome

Assembling the sterilizer

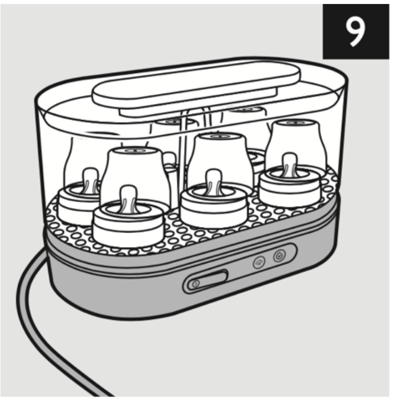

With only the small basket on the base to sterilize small items such as soothers (Figure 9).

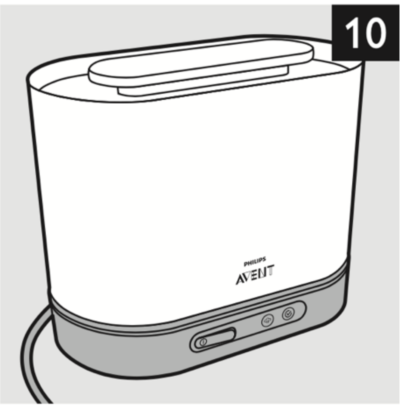

With only the large basket on the base to sterilize medium-sized items such as breast pumps, toddler plates or toddler cutlery (Fig. 10).

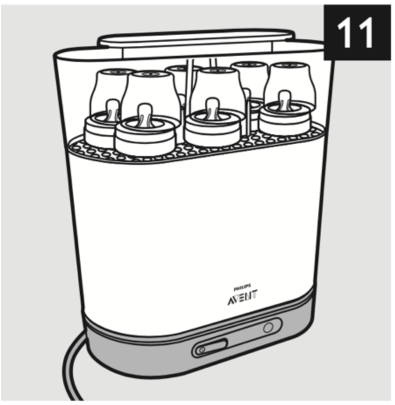

With the large basket and the small basket on the base to sterilize up to 6 11oz/330ml bottles.

Place the bottles upside down in the large basket and place the bottle dome caps, screw rings and teats in the small basket (Fig. 11).

Note: Make sure all parts that need to be sterilized are completely disassembled and place them in the basket with their openings pointing down to prevent them from filling up with water.

Source: Philips AVENT SCF284, SCF285, SCF286, SCF287 Manual www.philips.com/welcome

Using the appliance

- Use for example a clean baby bottle and pour exactly 100ml tap water directly into the water reservoir of the base.
- Assemble the sterilizer and place the items to be sterilized in it.
- Place the lid on top of the small or large basket
- Put the plug in the wall socket.
- Press the power-on button to switch on the appliance
- When the sterilization process is finished, the appliance switches off automatically for the SCF285/SCF284 sterilizer type and after 3 minutes for the SCF287/SCF286 type
- Remove the lid.
- Note: Wait until the appliance has cooled down before you remove the lid. Be careful, hot steam may come out of the sterilizer.
- Remove the bottles and other items from the basket. Use the tongs to remove the smaller items.
- Note: Be careful, the sterilized items may still be hot.

- Note: Make sure you wash your hands thoroughly before you touch sterilized items.
- Unplug the appliance and wait until the appliance has cooled down completely.
- Pour out any remaining water from the water reservoir and wipe the water reservoir dry.
- Note: The appliance does not work for 10 minutes after a sterilizing cycle; it needs to cool down first.

2. Sterilization of Symphony® Breast Pump Kit element

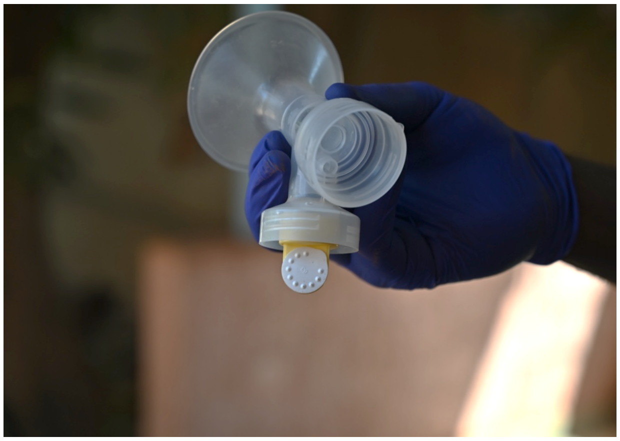

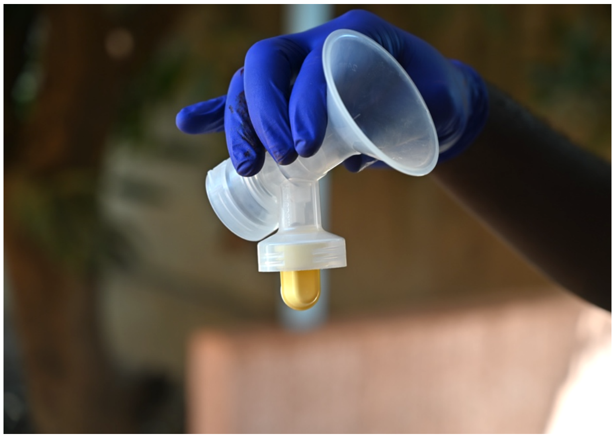

Kit elements: Valve (in white), Valve holder (yellow)

- Kit elements are not necessary to sterilize before first use.
- After collecting milk, sterilize the valve and its support. Clean the membrane with an alcohol pad.
- Let the kit dry then store it in a bag containing a desiccant, note the ID number of the participant.
- The morning of the next maternal milk collection, sterilize the participant's kit a second time.
- At the end of this collection, sterilize the kit again.

  Note: The bottles used to provide milk to children during body composition activity, should never be resealed after sterilization.

3. Breastmilk samples collection procedure

– Ask the woman which breast she last used to breastfeed the child and prepare the opposite breast for collection.

– Open the Symphony® Breast Pump Kit. Adapt the breast valve to the bottle. Connect the rounded (white) end of the tubing to the breast valve; connect the triangular (yellow) end of the tubing to the cap. Make sure the protective membrane covers the inside of the cap. Fit the cap to the pump and make sure it fits properly.

– Clean the breast twice with the BDZ pad.

> The Symphony® breast pump has two expression programs:
>
> - The INITIATE program helps pump-dependent mothers successfully initiate milk production. The program mimics the irregular sucking pattern of a full-term infant's first days of lactation, sometimes sucking faster, sometimes resting. Never use this program.
>
> - The MAINTAIN program is designed to stimulate milk production once lactation is activated (milk supply), in order to boost and maintain adequate milk production.

– To start the MAINTAIN program, press the left button "♦ ". A "Start" message followed by "INITIATE pressS" will appear. Do not touch anything. Wait 10 seconds and the pump will start the MAINTAIN program by default. The message "Start MAINTAIN" will appear, followed by three small drops of " ♦♦♦ ".

– To start, set the suction to 10 drops and ask the woman if this does not cause discomfort. If it does not cause discomfort, increase the suction drop by drop until it does cause discomfort and come to the previous drop.

– Wait for the milk to start flowing.

After two minutes, the pump will automatically switch from phase 1 "stimulation" to phase 2 "expression".

- Stimulation: phase 1 is indicated by the small drops on the screen " ♦♦♦♦♦♦♦♦♦♦ ". Its objective is to stimulate the lactation process.
- Expression: Phase 2 is indicated by the black rectangles on the screen "██████████". Its purpose is to pump milk.

If the milk has not started flowing by the end of the second phase, and the pump automatically switches to the expression phase (phase 2), return to the "stimulation" phase by pressing the "⇩♦♦" button once.

If the milk starts to flow before the two-minute period expires, press the «⇩♦♦» button to switch to the expression phase.

- Once the milk starts to flow, cover the plastic bottle with aluminum foil to protect it from the sun. completely empty the breast.
- Once the breast has been emptied, remove the Symphony pump kit and keep it in the original packaging.
- Check the level:
  - a. If the volume of milk exceeds the indicated level 1 US OZ (25mL) proceed to pipetting (and then to step 10 of the SOP).
  - b. If, after emptying the first breast, the amount of milk is less than 1 US OZ, repeat steps 1 to 8 (1. Clean the breast; 2. Adjust the pump; 3. Empty the breast completely). Mark that you have combined the milk from both breasts into one bottle on the collection form
- Turn the bottle upside down (invert) 3 times to mix, and using the Finntip™ Flex 1 - 200 µl pipet transfer 1.8 mL of the milk into 4 bar-coded Cryotubes covered with aluminum foil. Close

the tubes and place them in the Delta-T® cooler bag (10L) on a rack, before collecting the saliva.

> Steps 11 and 12 will only be done during breast milk collection occurring on the same day as the body composition activities.

- After collecting the milk samples in the 4 bar-coded Cryotubes, use the breast pump on the opposite breast to collect the maximum amount of milk for the baby.
- Give the bottle of milk to the baby.

4. Storage

- Transferred the 4 bar-coded Cryotubes into liquid nitrogen within 4 hours after collection of breastmilk.
- The 4 bar-coded Cryotubes will be put away in the -80°c later in the laboratory.

5. Troubleshooting & error and interferences

- If a participant has breastfed their children multiple times before the scheduled collection, they may not have enough milk at the time of collection. In this case, it would be better to reschedule the collection for a later date when the participant is likely to have a sufficient amount of breastmilk for sampling.
- Properly affixing and waiting a few seconds for the labels to adhere is crucial to prevent them from coming off the tubes once they are stored in the liquid nitrogen cylinder. Careless application of the labels by team members can cause this issue. To avoid this problem, it is essential to take the time to apply the labels to the entire surface of the tubes before storing them.

Collection of colostrum samples: Standard Operating Procedures (SOP) for the DenBalo Study

Lionel Olivier Ouédraogo,[1,2] Trenton Dailey-Chwalibóg,[1] Kelsey Fehr,[3] Natalie Rodriguez,[3] Meghan B. Azad,[3] Moctar Ouédraogo,[4] Anderson Compaoré,[4] Cheick Ahmed Ouattara,[4,5] Carl Lachat,[1] Laéticia Céline Toé,[1,6]

Abstract

Standard operating procedures are crucial to guarantee sample collection consistency and quality during a study. The quality of biological samples is all the more important when researchers aim to have a multi-omics approach. Many studies have been carried out in developed countries explaining the procedures used for biological sample collection for multi-omics analysis (Koh and al., 2022; Zreloff and al., 2023; Zubeldia-Varela and al., 2020). However, few to none of these studies were carried out in Africa, where working conditions are completely different and sometimes rudimentary.

This standard operating procedure (SOP) provides a guideline for colostrum sample collection in mothers enrolled in the study "Description and Comparison of Biological Vulnerability in Small, vulnerable newborns versus Healthy community controls in Urban Burkina Faso (DenBalo) Study: Gut Microbiota, Immune System, and Breastmilk Assembly and Development in the First Days and Weeks of Life" (ONZ-2022-0500, 050-2022/CEIRES du 16 September 2022). Colostrum samples will be distributed to multiple laboratories for analysis of macronutrients, micronutrients, oligosaccharides, growth factors, immunoglobulins, cytokines, metabolites and microbes.

The aim of this SOP is to establish a standardized process for colostrum sample collection ensuring consistency and quality of the sample collection process. To that purpose, this SOP details the steps needed to perform high quality sample collection, including material and tools preparation, specimen collection, labeling, handling and storage, and the list of required tracking documents to fill out during the process. By following this SOP, healthcare professionals can optimize the quality and reliability of colostrum samples collected. Our SOP could be a reference guideline on the collection of colostrum samples in Africa, specifically in nursing mothers during the neonates 'first few weeks of life.

Keywords: Standard Operating Procedure (SOP), colostrum sample, macronutrients, micronutrients, oligosaccharides, growth factors, immunoglobulins, cytokines, metabolites, microbes, early weeks of life.

---

[1] Department of Food Technology, Safety and Health, Faculty of Bioscience Engineering, Ghent University, Ghent, Belgium
[2] Centre Muraz, Bobo-Dioulasso, Burkina Faso
[3] Manitoba Interdisciplinary Lactation Centre (MILC), Department of Pediatrics and Child Health, University of Manitoba: Winnipeg, Manitoba, CA
[4] Agence de Formation de Recherche et d'Expertise en Santé pour l'Afrique (AFRICSanté)
[5] École Doctorale de Santé Publique, Université Nazi Boni, Bobo-Dioulasso, Burkina Faso
[6] Unité Nutrition et Maladies Métaboliques, Institut de Recherche en Sciences de la Santé (IRSS)

Notes

- Colostrum production lasts for the first three to five days after delivery and the onset of lactation
- Colostrum is thick and yellowish (lemon-colored).
- Collect colostrum with 80ml bottles only.
- Do not use the breast pump for colostrum collection.
- Tips to help milk let-down:
    - Ask the participant to relax.
    - Perform a breast massage ;
    - Apply warm compresses ;
    - Ask the participant to imagine that her baby is breastfeeding.
    - The breast massage can be performed by the health worker or by the participant herself.
    - Massage should not be painful.
- Manual breast expression is performed by the participant herself with the assistance of the DENBALO team.

MATERIALS

MATERIALS	QUANTITIES
Pen	01
Soap	01
Benzalkonium Chloride (BDZ) wipes	04
80ml Plastic bottle	02
Aluminum foil	01
2ml bar-coded sterile Cryotubes	06
Cryolabels	01 sheet
Rack	01
1ml Pasteur pipette LDPE (Low-Density Polyethylene)	01
Delta-T® cooler bag (10L)	01
Biohazard Bag	01

PROCEDURE

1. Breast massage

- DENBALO team health workers must wash their hands with soap.
- Ask the woman which breast she last breastfed on and prepare the opposite breast for collection.
- In addition to using one of the tricks to promote milk let-down, the participant must perform a breast massage to collect colostrum:
- The health worker must show the participant the different steps :
  - Ask the participant to hold the breast with her less skillful hand, the four fingers well below the breast.
  - With the four fingers of the opposite hand, using all four fingers, massage gently in a circular motion from the top towards the nipple.
  - Perform this massage from top to bottom, left to right, right to left, each time moving from the outside towards the nipple. The participant can help herself with gravity by leaning forward.

2. Manual breast expression

- Wash your hands with soap
- Clean the breast to be collected twice with the Benzalkonium Chloride (BDZ) wipes.
- Use an 80ml plastic bottle wrapped with aluminium foil to protect the sample from sunlight.
- To facilitate the ejection reflex, you can gently massage your breast.
- Lean forward a little so that the milk falls into the 80ml plastic bottle covered with aluminium foil.

- Form a "C" with your thumb and forefinger. Their ends should face each other, like pliers (see photo no. 1).

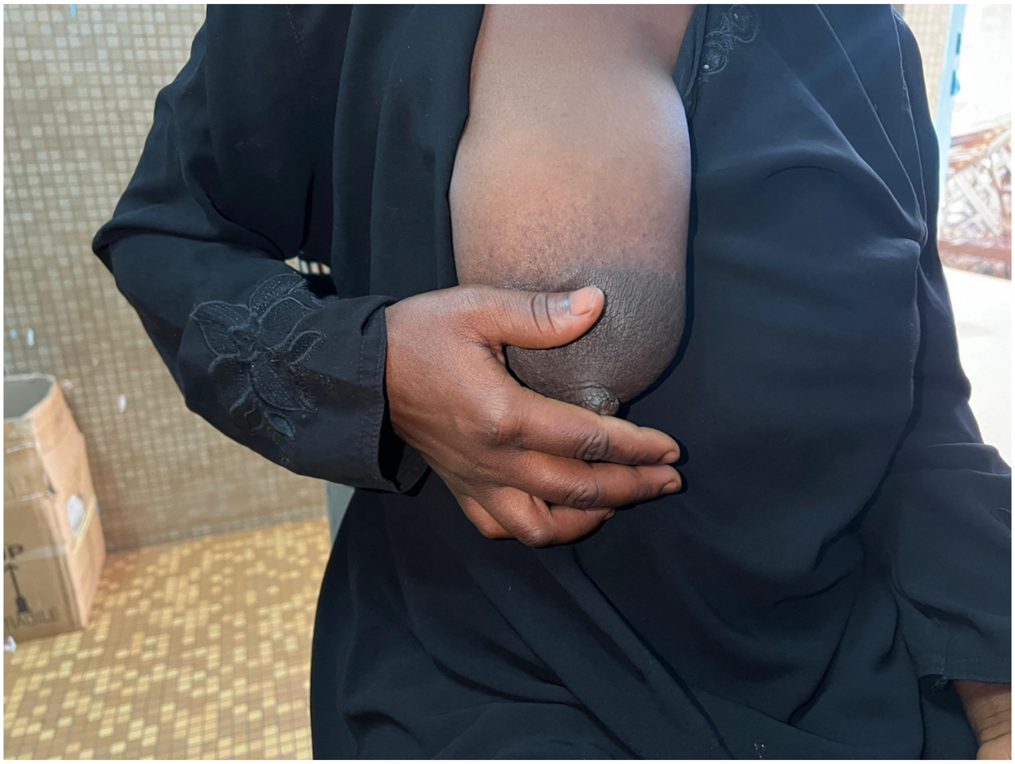

Photo 1: Thumb and forefinger forming a “C”

- Place your thumb and forefinger on either side, 2 to 5 cm from the nipple. You'll find the ideal distance with practice (see photo no. 2).

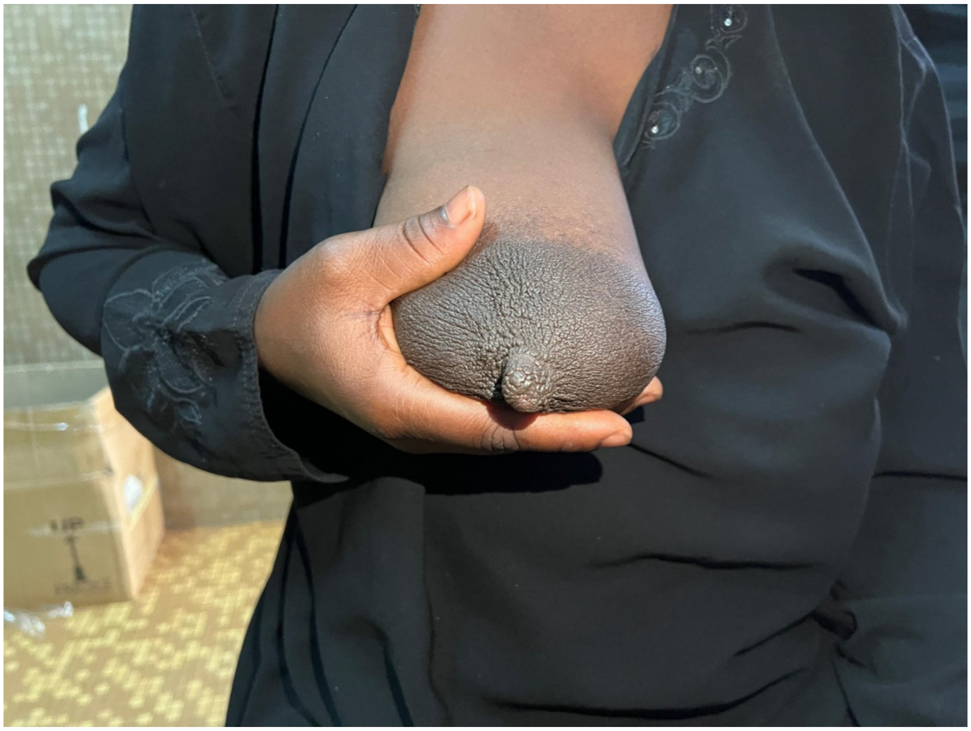

Photo 2: Thumb and forefinger on either side, 2 to 5 cm from the nipple

- Press your fingers into your breast, pushing horizontally towards the ribs (see photo no. 3).

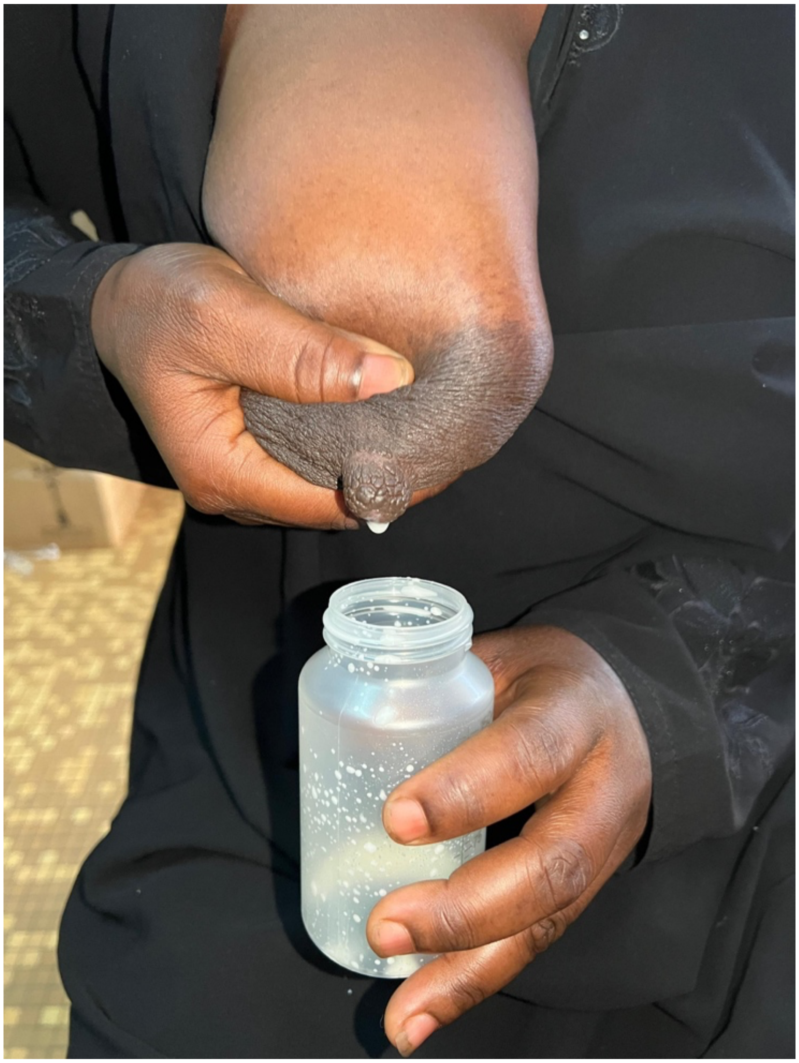

Photo 3: Thumb and forefinger press into the breast

- Keeping your fingers depressed, bring your thumb and forefinger together, closing them as if they were pliers. It's not necessary to exert strong pressure. The movement should not leave a mark on the breast, nor should it hurt.
- Repeat the pincer movement several times, rhythmically, just as your baby does when suckling.
- Be careful not to slide your fingers over the skin. Keep your fingers firmly planted on your breast at all times, without ever stretching the nipple. Stretching the nipple would be painful and not very effective.

- Move your fingers around your breast to empty it.
- Milk flows drop by drop at first, then begins to emerge in small streams. With practice, you willl become faster and more efficient.
- Collect 10ml of milk in the 80ml plastic bottle covered with aluminium foil.
- After collection, ask the participant to wash the collected breast with water to remove any residual Benzalkonium Chloride (BDZ) wipes.

3. Aliquots preparation

- Invert the 80ml plastic bottle 5 times and using the 1ml Pasteur pipette LDPE (Low-Density Polyethylene)transfer 1.8mL of milk into 4 x 2ml bar-coded sterile cryotubes previously covered with aluminium foil.
- Store aliquots in a Delta-T® cooler bag (10L)

4. Storage

- Samples must be stored in the liquid nitrogen cylinder within 4 hours after collection

Bibliography

Koh, E. J., Kim, S. H., & Hwang, S. Y. (2022). Sample management: a primary critical starting point for successful omics studies. *Molecular and Cellular Toxicology*, *18*(2), 141–148. https://doi.org/10.1007/s13273-021-00213-x

Zreloff, Z. J., Lange, D., Vernon, S. D., Carlin, M. R., & Cano, R. J. (2023). Accelerating Gut Microbiome Research with Robust Sample Collection. *Research & Reviews. Journal of Microbiology and Biotechnology*, *12*(1), 33–47.

Zubeldia-Varela, E., Barber, D., Barbas, C., Perez-Gordo, M., & Rojo, D. (2020). Sample pre-treatment procedures for the omics analysis of human gut microbiota: Turning points, tips and tricks for gene sequencing and metabolomics. *Journal of Pharmaceutical and Biomedical Analysis*, *191*, 113592. https://doi.org/10.1016/j.jpba.2020.113592

Immunostaining: Standard Operating Procedures (SOP) for the DenBalo Study

Simon J. Tavernier,[1,2,3] Véronique Debacker,[2] Lionel Olivier Ouédraogo,[4,5] Laeticia Celine Toé,[4,6] Trenton Dailey-Chwalibóg,[4] Carl Lachat,[4] Filomeen Haerynck,[1,2]

Abstract

Standard operating procedures are crucial to guarantee sample processing consistency and quality during a study. Many studies have been carried out in developed countries explaining the procedures used for immunostaining. However, a little of those studies were carried out in Africa where working conditions are totally different and sometimes challenging.

This standard operating procedure (SOP) provides a guideline for immunostaining procedure in whole blood collected from mothers and neonates enrolled in the study "Description and Comparison of Biological Vulnerability in Small, vulnerable newborns versus Healthy community controls in Urban Burkina Faso (DenBalo) Study: Gut Microbiota, Immune System, and Breastmilk Assembly and Development in the First Days and Weeks of Life" (ONZ-2022-0500, 050-2022/CEIRES du 16 September 2022). Immunostained blood samples are stored long-term according to study protocol and are immunophenotyped using flowcytometry at the end of the study. In parallel, plasm was collected for enumeration of soluble biomarkers.

The aim of this SOP is to establish a standardized procedure for blood samples immunostaining ensuring the process consistency. To that purpose this SOP details steps to follow to undertake a good sample processing including material and tools preparation, specimen processing, labeling, handling and storage, antibodies mixture preparation and the list of required tracking documents to fill out during the process. By following this SOP, laboratory technicians can optimize the quality and reliability of the immunostained blood samples. Our SOP could be a reference guideline blood samples immunostaining in Africa specifically tailored for immunophenotyping.

Keywords: Standard Operating Procedure (SOP), Immunostaining, Immunophenotyping, Flowcytometry, Blood samples, Neonates, Mothers

---

[1] Primary Immunodeficiency Research Lab (PIRL), Department of Internal Medicine and Pediatrics, Ghent University, Ghent, Belgium

[2] Center for Primary Immunodeficiency, Jeffrey Modell Diagnosis and Research Center, Ghent University Hospital, Ghent, Belgium.

[3] Center for Medical Genetics, Ghent University Hospital, Ghent, Belgium

[4] Department of Food Technology, Safety and Health, Faculty of Bioscience Engineering, Ghent University, 9000 Ghent, Belgium

[5] Centre Muraz, Bobo-Dioulasso 01 BP 390, Burkina Faso

[6] Unité Nutrition et Maladies Métaboliques, Institut de Recherche en Sciences de la Santé (IRSS)

Notes

- Sampling logging content:
  - Generate sample number in the logbook.
  - Indicate the population type (small vulnerable/full term/adult)
  - Indicate sample specific timepoint:
    - Note down location and time of sampling.
    - Note down time of arrival in laboratory.
    - Note down volume of sample and unexpected aberrations of the sample (clogs, etc)
    - Note down the staining procedure start.
    - Note down the overnight incubation time.
    - Note down volume of plasma.
    - Note down the plasma freezing time.
    - Note down stained blood freezing time.
    - Note down location in freezer of plasma and cells.

MATERIALS

MATERIALS	QUANTITIES
Pen	01
Permanent marker	01
Laboratory log	01
Flow laminar	01
Pipet 50 - 250 µl	01
Pipet 100 – 1000 µl	01
1,5ml Pipet Pasteur	03
Pipet 0,5 - 10µl	01
Pipet 2-20µl	01
Pipet automatic	01
50 – 250µl tips	As needed
200 µl tips	As needed
1000 µl tip	As needed

1-10 µl sterile tips	As needed
10ml sterile tips	As needed
Hettich® ROTINA 420 R Centrifuge	01
VWR® MiniStar Microcentrifuge	01
Biosan® Microspin 12 High-speed Mini centrifuge	01
Labbox ® Vortex Stirrer with speed control V05	01
1,5ml eppendorf tubes	03
2ml Bar-coded cryotube	03
Eppendorf Isotherm System IsoRack	03
Phosphate-Buffered Saline (PBS)	20ml
Distilled water	20ml
Aluminium foil roll	01
15ml Tube	03
Antibodies	As needed
Buffer Reagents	As needed
Laboratory logbook	01

PROCEDURE

1. Preparation of buffers

	Buffers	Volume
Cytodelics fixation buffer (for 1 sample)	Cytodelics fixation concentrate	500ul
	Cytodelics fixation diluent	500ul
Cytodelics lysis buffer (for 1 sample)	Cytodelics 4X lysis buffer	1000ul
	ddH2O	3000ul
Cytodelics washing buffer (for 1 sample)	Cytodelics 5X wash buffer	800ul
	ddH2O	3200ul

2. Staining mixture

- **Staining mixture 1**

Parameter	Marker	Label	Volume by sample (ul)
UV510	CD8	BUV496	1
UV510	CD19	BUV496	2,5
UV605	CD62L	BUV615	1

UV670	CD11c	BUV661	2,5
V450	FceR1	BV421	1
V525	LD	eFluor506	1
B530	CD123	FITC	1
B710	IgD	BB700	1
B780	CD56	BB790-P	1
Y610	CD127	PE-Dazzle594	2,5
Y780	CD27	PE-Cy7	2,5
R730	CD38	APC-R700	1
R786	CD161	APC-Fire750	5
Fc block	Fc Block	/	5
Biotin	Va7.2	biotin	0,63
			28,63
Volume dPBS to add			21,37
Volume brilliant staining buffer to add			50
Total volume			100

– **Staining mixture 2**

	Marker	Label	Volume by sample (ul)
UV735	CD34	BUV737	2,5
V450	TCRgd	BV421	1
V677	CD39	BV650	2,5
V710	CD25	BV711	1
Y585	CD137	PE	1
			8
Volume dPBS to add			42
Volume brilliant staining buffer to add			50
Total volume			100

3. Staining protocol

Day one

– Add 200ul of blood to 1 x 1,5ml eppendorf tubes (Use Pipet 50 - 250 µl).

Note: If there is not enough blood to have the 200µl, collect at least 100µl of blood.

– Spin 3min 3000g 4C in Biosan® Microspin 12 High-speed Mini centrifuge.

- Prepare one 2ml Bar-coded cryotubes and put it on Eppendorf Isotherm System IsoRack. This 2ml Bar-coded cryotubes will be used to transfer the plasma after centrifugation.

  Note: Correctly label tube for plasma. .
- Take off the supernatant plasma, transfer to 2ml Bar-coded cryotube and store in the -80C freezer (Use Pipet 50 - 250 µl).

  Note: Immediately put the plasma in -80°c.
- Replace the plasma by equal volume of Phosphate-Buffered Saline (PBS) (80µl) and mix cell pellet by gently pipetting up and down (Use Pipet 50 - 250 µl).
- Keep on ice until ready for staining.
- Prepare staining mix 1 (Use Pipet 0,5 - 10µl and Pipet 2-20µl).

  Note 1: For the very first time that you use the antibodies, centrifuge them 3000g for 10min to pellet aggregates. Then each time you use the antibodies, a quick spin 3000g for 10 seconds.

  Note 2: Never forget that as soon as you add the antibodies mixture you must protect your samples from the light by using Aluminium foil roll.
- Add 100uL of staining 1 (Use Pipet 50 - 250 µl)
- Vortex with Labbox ® Vortex Stirrer with speed control V05 and stain 30min at 4°c.

  Note: Put it in the fridge 4°c for 30min.
- Add 1mL of PBS (Use Pipet 100 – 1000 µl).
- Centrifuge the samples at 300g for 5 min at room temperature with Biosan® Microspin 12 High-speed Mini centrifuge.
- Aspirate the supernatant but leave some volume at the bottom, do not decant (Use 1,5ml Pipet Pasteur)
- Prepare staining mix 2 (Use Pipet 0,5 - 10µl and Pipet 2-20µl)

Note 1: For the very first time that you use the antibodies, centrifuge them 3000g for 10min to pellet aggregates. Then each time you use the antibodies, a quick spin 3000g for 10 seconds.

Note 2: Never forget that as soon as you add the antibodies mixture you must protect your samples from the light.

- Add 100uL of staining 2 (Use Pipet 50 - 250 µl).
- Vortex by using Labbox ® Vortex Stirrer with speed control V05 and stain 16 to 20 hours at 4C (protected from the light in fridge).

  Note: Put it in the fridge 4°c for 16-20h.

Day two

- Prepare 2 x 15ml tubes (one for the lysis buffer and one for the wash buffer) and label them.
- Prepare the fixation buffer, it has to be at room temperature (Use Pipet 100 – 1000 µl).
- Add 1 ml fixation buffer to 1,5ml eppendorf tubes (Use Pipet 100 – 1000 µl).
- Incubate the samples 15 min at room temperature (RT) and vortex multiple times during the incubation-step by using Labbox ® Vortex Stirrer with speed control V05.
- Prepare lysisbuffer (Use Pipet automatic).
- Transfer the fixed cells from the 1,5ml eppendorf tubes to 15ml tube containing the 4ml of lysis buffer and vortex once with Labbox ® Vortex Stirrer with speed control V05 (Use Pipet 100 - 1000 µl).
- Incubate the samples 10-20 min until the sample is crystal clear red (usually this is OK after 10 min for 200µl whole blood).
- Do not proceed without complete red blood cells lysis.

  Note: If the red blood cells are not lysed yet after 20min, centrifuge the sample at 300g for 5 min at room temperature by using Hettich® ROTINA 420 R Centrifuge, remove the

supernatant get your pellet and resuspend it in 4ml of lysis buffer. Incubate it for another 10min.

- Centrifuge the samples at 300g for 5 min at room temperature by using Hettich® ROTINA 420 R Centrifuge.
- Aspirate the supernatant but leave some volume at the bottom, do not decant! (Use 1,5ml Pipet Pasteur).
- Add 4ml of 1/5 diluted wash buffer and resuspend the pellet (Use pipet automatic with 5ml tip
- Centrifuge the samples at 300g for 7 min at room temperature by using Hettich® ROTINA 420 R Centrifuge.
- Aspirate the supernatant but leave some volume at the bottom, do not decant! (Use 1,5ml Pipet Pasteur).

  Note: If the pellet remains red, repeat the washing step once more.
- Resuspend the sample in 250µl of CRYO#20 and transfer the samples to a 1,5ml eppendorf tube.

  Note: Make sure the samples remain cooled (work quickly or on ice) (Use Pipet 50 - 250 µl).

  Note: We use opaque eppendorf tubes to protect fluorochromes to unintented light exposure.
- Store the samples at -80°C

Collection of vaginal samples in mothers: Standard Operating Procedure (SOP) for the DenBalo Study

Lionel Olivier Ouédraogo,[1,2] Trenton Dailey-Chwalibóg,[1] Tanko Fatime Ramla,[3,4] Jo-Ann Passmore,[3] Heather Jaspan,[5] Moctar Ouédraogo,[6] Anderson Compaoré,[6] Cheick Ahmed Ouattara,[6,7] Carl Lachat,[1] Laéticia Céline Toé,[1,8]

Abstract

Standard operating procedures (SOPs) are crucial to guarantee sample collection consistency and quality during a study. The quality of biological samples is particularly important as researchers strive for a multi-omics approach. Many studies have been carried out in developed countries explaining the procedures used for biological samples collection for multi-omics analysis. However, few to none of those studies were carried out in Africa, where working conditions are totally different and sometimes rudimentary.

This SOP provides a guideline for vaginal samples collection in mothers enrolled in the study entitled "Description and Comparison of Biological Vulnerability in Small, vulnerable newborns versus Healthy community controls in Urban Burkina Faso (DenBalo) Study: Gut Microbiota, Immune System, and Breastmilk Assembly and Development in the First Days and Weeks of Life" (ONZ-2022-0500, 050-2022/CEIRES du 16 September 2022). Vaginal samples are dedicated to vaginal microbiome profile analysis.

The aim of this SOP is to establish a standardized procedure for collecting vaginal samples, ensuring consistency in the process. To achieve this aim, the SOP outlines the steps necessary for good sample collection including material and tools preparation, specimen collection, labeling, handling, storage and completion of required tracking documents. Adhering to this SOP will enable healthcare professionals to enhance the quality and reliability of collected vaginal samples. Furthermore, our SOP could serve as a reference guide for vaginal samples collection in Africa, specifically tailored for the analysis of vaginal microbiome profiles.

Keywords: Standard Operating Procedure (SOP), Pregnant women, Delivery, Vaginal swab, Vaginal microbiome profile

[1] Department of Food Technology, Safety and Health, Faculty of Bioscience Engineering, Ghent University, Ghent, Belgium
[2] Centre Muraz, Bobo-Dioulasso, Burkina Faso
[3] Institute of Infectious Disease and Molecular Medicine, Faculty of Health Sciences, University of Cape Town, South Africa
[4] The Medical Research Centre, Institute of Medical Research and Medicinal Plant Studies (IMPM), Ministry of Scientific Research and Innovation, Yaoundé, Cameroon
[5] Seattle Children's Research Institute | 307 Westlake Ave Seattle 98109 | (206) 8543336
[6] Agence de Formation de Recherche et d'Expertise en Santé pour l'Afrique (AFRICSanté)
[7] École Doctorale de Santé Publique, Université Nazi Boni, Bobo-Dioulasso, Burkina Faso
[8] Unité Nutrition et Maladies Métaboliques, Institut de Recherche en Sciences de la Santé (IRSS)

Notes

- Warn the participant that discharge, or bleeding may occur after a vaginal swab.
- Vaginal samples collected with the OMR-130 kits are performed on delivery day by both DENBALO's staff and maternity ward staff.
- Only DENBALO's staff collect vaginal samples using sterile swabs during echography visit days and participant home visits. Two sterile swabs are used for each collection, labelled va1c and va2s.
- The steps 2.6 to 2.17 are performed twice (once for va1c collection and once for va2s collection)
- Do not performed vaginal samples collection when the mother is seen in labor after rupture of membranes.

MATERIALS

Please ensure that the following material and tools are available in the correct quantities:

MATERIALS	QUANTITIES
OMR-130 kit	01
Pen	01
Soap	01
Gloves	02 pairs
Cryolabels	01 sheet
Sterile swab	01
2ml bar-coded Cryotubes	02
Scissors	01
Delta-T® cooler bag (10L) containing icepacks	01
Rack	01
Samsung Tablet	01

PROCEDURE

In the framework of our study, we will collect vaginal samples using two different methods: the OMR-130 kit and sterile swabs. Vaginal sampling with the OMR-130 kit will be performed only on the day of delivery, with samples preserved in a tube containing a stabilizing liquid. In contrast,

vaginal sampling with sterile swabs will be conducted during the prenatal follow-up period (at 29-30 weeks and 33-34 weeks), with samples kept dry in 2ml bar-coded Cryotubes.

1. **Vaginal sampling with the OMR-130 kit**

- The healthcare provider should perform the following steps:
- Place the participant in the supine position and explain the purpose and method of vaginal sampling. Reassure the participant as much as possible that the method is painless.
- Wash hands thoroughly with soap and water. Ensure that hands are dry before beginning the procedure.
- Wear gloves
- Stand or sit comfortably to be in the best position to perform the collection.
- Take the OMR-130 kit and extract the tube containing the stabilizing liquid (Fig 1)

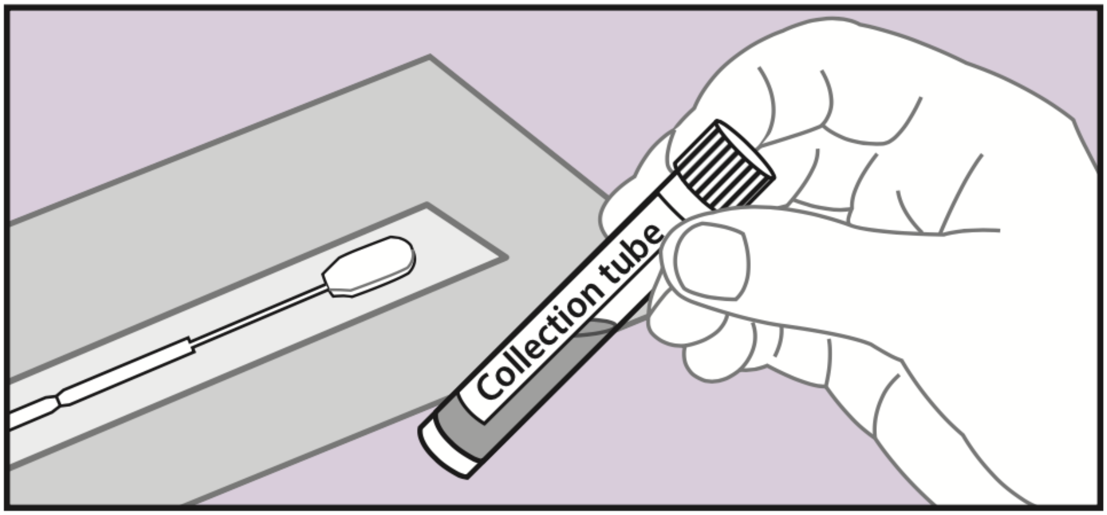

Figure 1: Extracting the tube containing the medium

Source:OMNIgene•VAGINAL | OMR-130 Manual

https://www.dnagenotek.com/us/support/collection-instructions/omnigene-vaginal/OMR-130.html

- Set aside the tube on a clean surface for later use.

- Extract the swab from the package by grasping the handle (Fig 2).

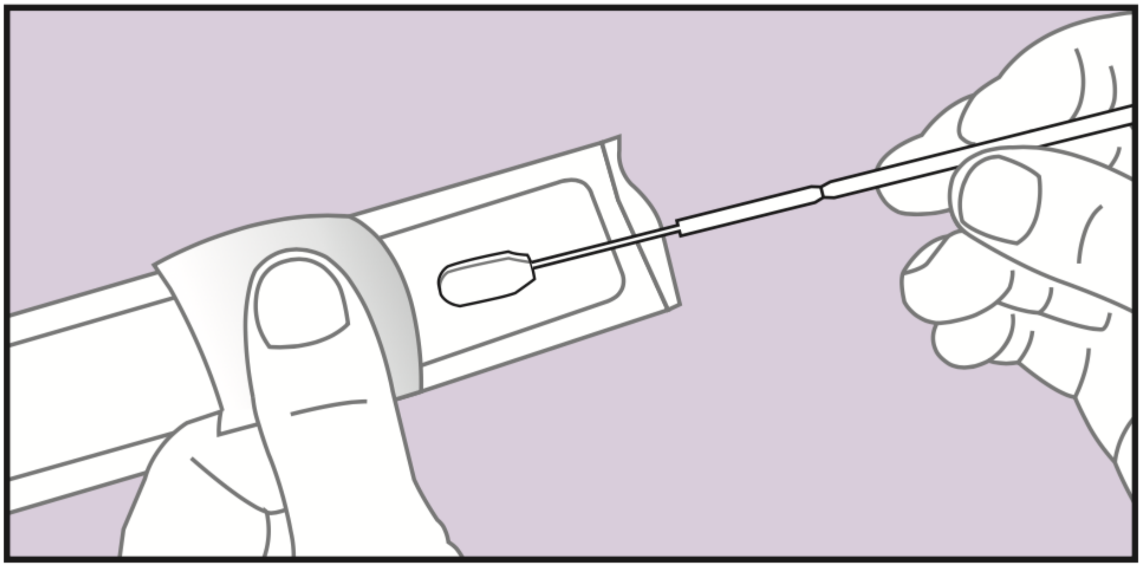

Figure 2 : Extract the swab from the package by holding only the handle

Source:OMNIgene•VAGINAL | OMR-130 Manual https://www.dnagenotek.com/us/support/collection-instructions/omnigene-vaginal/OMR-130.html

- Note 1: Hold the swab only by its handle. Avoid touching the swab below the break point (Fig 3).
- Note 2: Avoid touching any surface with the swab after it has been removed from its packaging.

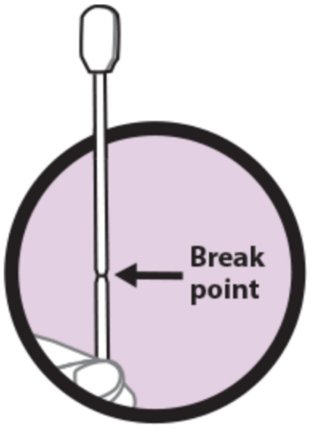

Figure 3 : Break point presentation

Source: OMNIgene•VAGINAL | OMR-130 Manual https://www.dnagenotek.com/us/support/collection-instructions/omnigene-vaginal/OMR-130.html

- Inform the participant that you will begin sample collection.
- Gently spread the participant's labia majora and minora and slowly and gently insert the swab 3-5 cm into the vagina (Fig 4).
- Note 3: Avoid twisting the swab during sampling.

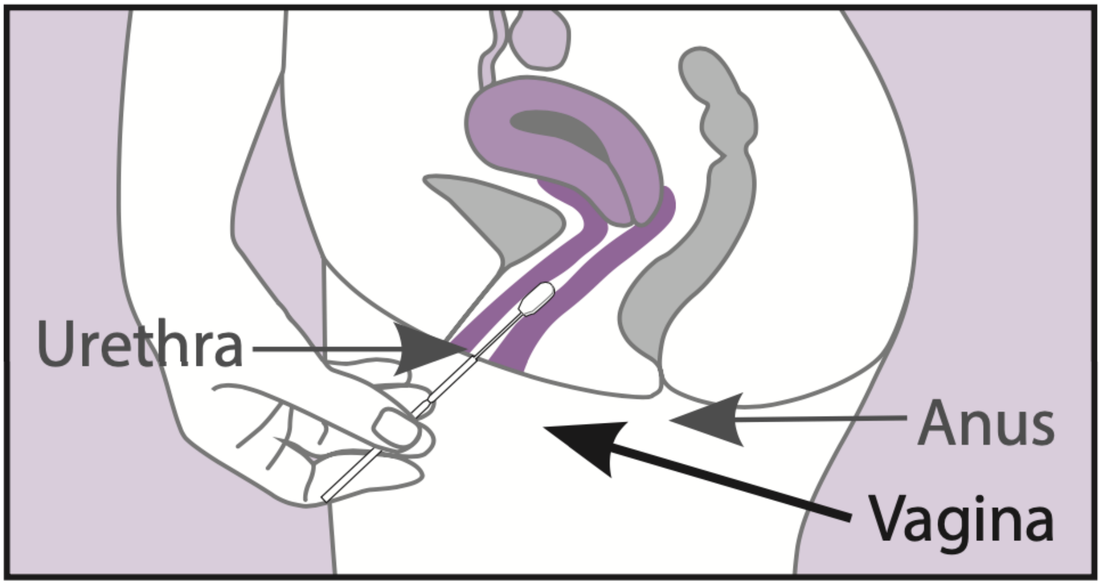

Figure 4: Introduction of the swab into the vagina and beginning of the sampling

Source: OMNIgene•VAGINAL | OMR-130 Manual https://www.dnagenotek.com/us/support/collection-instructions/omnigene-vaginal/OMR-130.html

- Conduct the sampling by gently scraping the vaginal walls in a circular motion. Perform several circular movements on both vaginal walls. This sampling procedure should last at least 20 seconds (Fig 5).

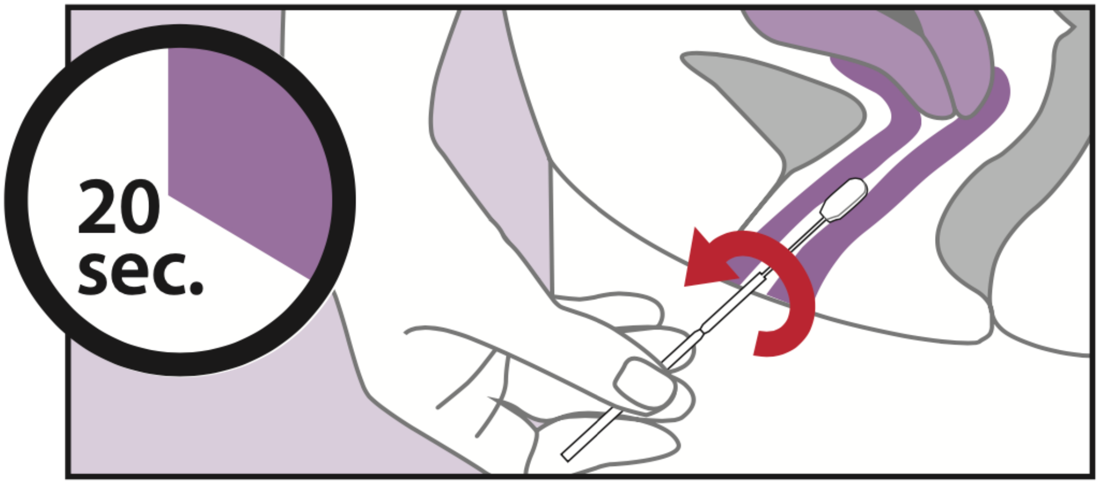

Figure 5: Vaginal sampling technique

Source: OMNIgene•VAGINAL | OMR-130 Manual https://www.dnagenotek.com/us/support/collection-instructions/omnigene-vaginal/OMR-130.html

- Note 4: Ensure that all sides of the swab have been used for vaginal sampling.
- Once sample collection is complete, carefully remove the swab from the vagina.
- Hold the swab firmly with one hand (avoid placing it on a surface or touching any objects) and use the other hand to open the tube containing the stabilizing liquid (Fig 6).

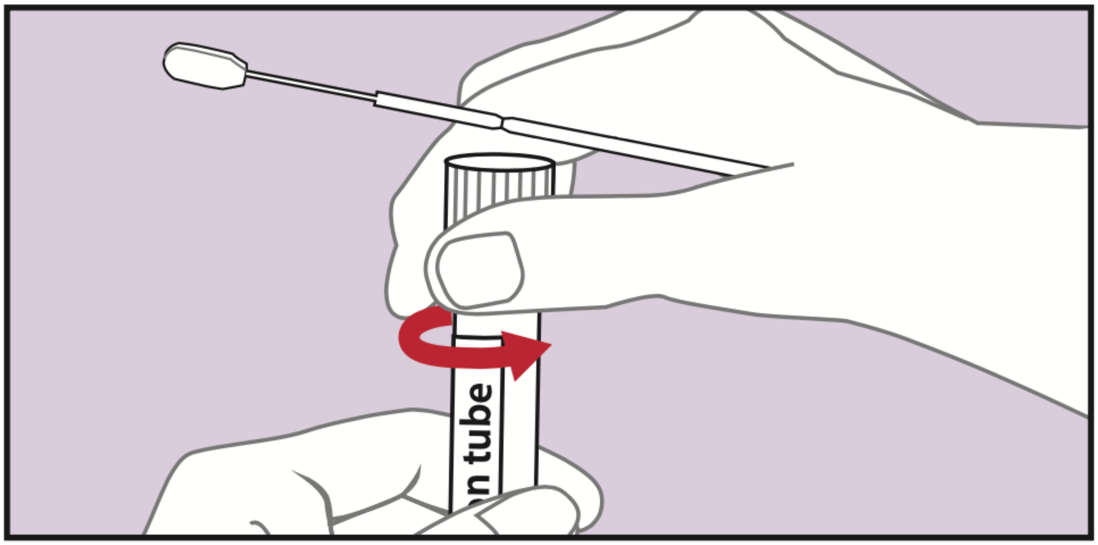

Figure 6 : Opening the sample tube with stabilizing liquid

Source: OMNIgene•VAGINAL | OMR-130 Manual

https://www.dnagenotek.com/us/support/collection-instructions/omnigene-vaginal/OMR-130.html

– Note 5: Be careful not to spill the stabilizing liquid contained in the tube.

– Note 6: Avoid placing the swab on a surface or allowing it to come into contact with any objects.

– Insert the swab into the tube and break it at the break point (Fig 7)

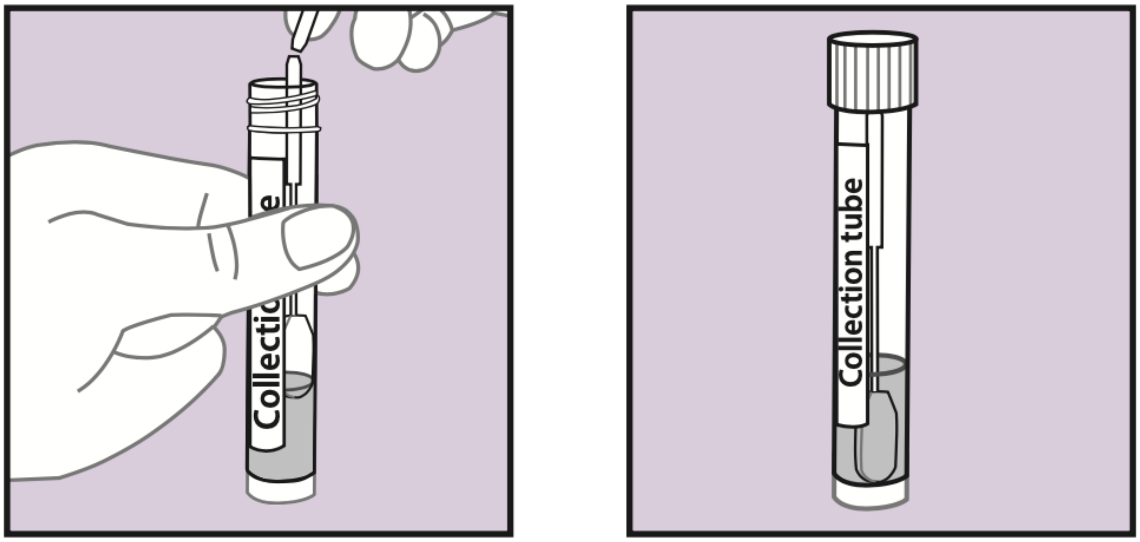

Figure 7: Inserting the swab into the tube containing the stabilizing liquid

Source: OMNIgene•VAGINAL | OMR-130 Manual

https://www.dnagenotek.com/us/support/collection-instructions/omnigene-vaginal/OMR-130.html

– Close the tube containing the swab tightly (Fig 8) and label it.

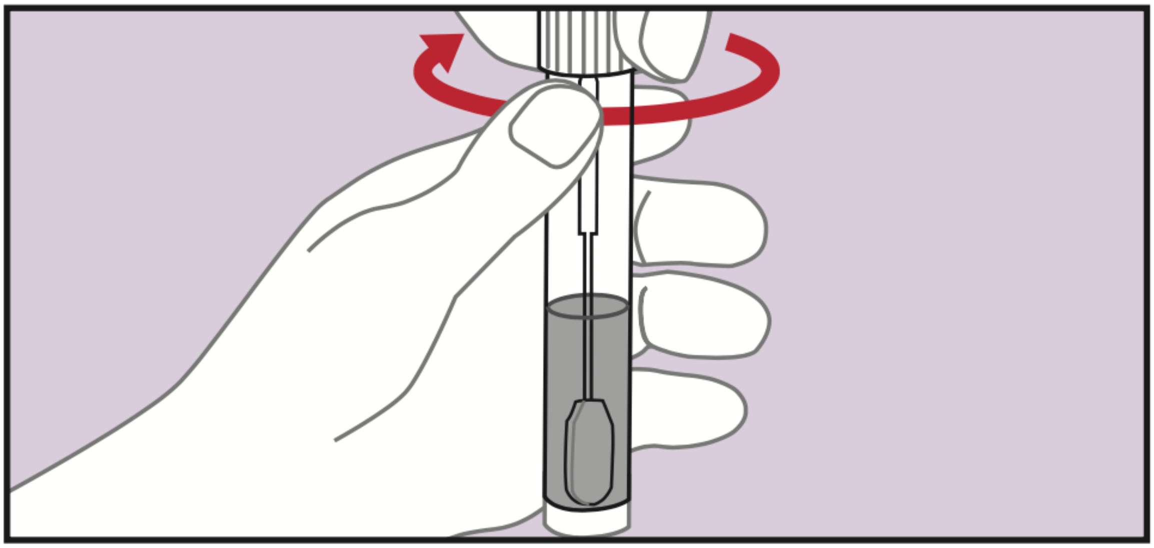

Figure 8 : Securing the Sample in a Stabilizing Liquid Tube

Source: OMNIgene•VAGINAL | OMR-130 Manual

https://www.dnagenotek.com/us/support/collection-instructions/omnigene-vaginal/OMR-130.html

– Fill in the CAPI (data collection sheet) that corresponds to the visit.

- Store the tube containing the swab at a temperature between 15°c and 25°c.

2. Vaginal sampling with sterile swabs

- The healthcare provider should perform the following steps:
- Place the participant in the supine position and explain the purpose and method of vaginal sampling. Reassure the participant to alleviate any concerns about discomfort during the procedure.
- Wash hands thoroughly with soap and water. Ensure that hands are dry before beginning the procedure.
- Wear gloves
- Stand or sit comfortably to be in optimal conditions to perform the collection.
- Retrieve the tube containing the sterile swab.
- Remove the swab from the package by grasping the handle.
- Note 1: Hold the swab by the handle only.
- Note 2: Avoid touching any surfaces with the swab once removed from its packaging.
- Inform the participant that you will begin sample collection.
- Gently spread the participant's vaginal lips and slowly and gently insert the swab 3-5cm into the vagina (Fig 4).
- Note 3: Do not twist the swab during sampling.
- Conduct the sampling by gently scraping the vaginal walls in a circular motion. Perform several circular movements on both vaginal walls. This sampling procedure should last at least 20 seconds (Fig 5).
- Note 4: Ensure that all sides of the swab have been used for vaginal sampling.

- Once sample collection is complete, gently remove the swab from the vagina.
- Hold the swab firmly in one hand (do not place it on a surface or touch an object) and with the other hand, open the 2ml bar-coded Cryotube.
- Insert the swab into the 2ml bar-coded Cryotubes and cut it with scissors, ensuring the swab fits inside the 2ml Cryotubes.
- Close the 2ml bar-coded Cryotube containing the swab tightly (Fig 8) and label it.
- Place the 2ml bar-coded Cryotube containing the swab in the Delta-T® Cooler Bag (10L).
- Complete the CAPI (data collection sheet) corresponding to the visit.
- Transport the 2ml bar-coded Cryotube containing the swab to the laboratory and store it at -80°c within 4-6 hours after sample collection.

Collection of umbilical cord blood samples: Standard Operating Procedure (SOP) for the DenBalo Study

Lionel Olivier Ouédraogo,[1,2] Trenton Dailey-Chwalibóg,[1] Yuri Bastos-Moreira,[1,3] Marthe De Boevre,[1] Moctar Ouédraogo,[4] Anderson Compaoré,[4] Cheick Ahmed Ouattara,[4,5] Carl Lachat,[1] Laéticia Céline Toé,[1,6]

Abstract

Standard operating procedures are crucial to guarantee sample collection consistency and quality during a study. The quality of biological samples is all the more important that researchers aim to have a multi-omics approach. Many studies have been carried out in developed countries explaining the procedures used for biological samples collection for multi-omics analysis. However, a limited number of studies have been carried out in an Africa setting, where working conditions are totally different and sometimes rudimentary.

This standard operating procedure (SOP) provides a guideline for umbilical cord blood samples collection in neonates enrolled in the study "Description and Comparison of Biological Vulnerability in Small, vulnerable newborns versus Healthy community controls in Urban Burkina Faso (DenBalo) Study: Gut Microbiota, Immune System, and Breastmilk Assembly and Development in the First Days and Weeks of Life" (ONZ-2022-0500, 050-2022/CEIRES du 16 September 2022). Umbilical cord blood samples are dedicated to black carbon exposure analysis and telomere length quantification.

The aim of this SOP is to establish a standardized process for umbilical cord blood samples collection ensuring consistency in samples collection. To that purpose, this SOP details steps to follow to undertake a good sample collection including material and tools preparation, specimen collection, labeling, handling and storage, and the list of required tracking documents to fill out during the process. By following this SOP, healthcare professionals can optimize the quality and reliability of umbilical cord blood samples collected. Our SOP could be a reference guideline on umbilical cord blood samples collection in Africa specifically tailored for black carbon exposure analysis and telomere length quantification.

Keywords: Standard Operating Procedure (SOP), Umbilical cord blood samples, Black carbon exposure analysis, Telomere length quantification, Newborns

---

[1] Department of Food Technology, Safety and Health, Faculty of Bioscience Engineering, Ghent University, Ghent, Belgium
[2] Centre Muraz, Bobo-Dioulasso, Burkina Faso
[3] Center of Excellence in Mycotoxicology and Public Health, MYTOXSOUTH® Coordination Unit, Faculty of Pharmaceutical Sciences, Ghent University, 9000 Ghent, Belgium.
[4] Agence de Formation de Recherche et d'Expertise en Santé pour l'Afrique (AFRICSanté)
[5] École Doctorale de Santé Publique, Université Nazi Boni, Bobo-Dioulasso, Burkina Faso
[6] Unité Nutrition et Maladies Métaboliques, Institut de Recherche en Sciences de la Santé (IRSS)

Notes

- Samples collected at night are performed by the maternity ward staff. Those collected during working hours are performed by DENBALO's staff.
- Umbilical cord blood samples collection should be done within a maximum of 30 minutes after placenta expulsion and before collection of placenta tissue samples.
- The maximum time limit from collection to storage of 2ml cryotubes in liquid nitrogen is 2 hours for DENBALO's staff.
- The maximum time limit from collection to storage of 2ml bar-coded cryotubes in the fridge (2°C-8°C) is 2 hours for the maternity ward staff.
- Steps 1.11 to 1.17 of the procedure are not performed by the maternity ward staff. After the step 1.10 staff store the samples.
- Collect cord blood in all preterm births, regardless of the time of birth. For term births, collect cord blood only during working hours.
- Follow this order of samples collection: collect,
    - First the cord blood in the 500-µL violet EDTA microtainer tube
    - Then the 10µL VAMS
    - And finally, the 4ml EDTA tube

MATERIALS

MATERIALS	QUANTITIES
Pen	01
Soap	01
Phosphate-buffered saline (PBS) solution (VWR International, USA)	At least 100ml
Sterile gauze	03
Tray	01
5-10CC syringes	01
18G needles (VWR International, China)	01

500-µl violet BD microtainer EDTA tube (BD, Franklin Lakes, NJ, USA)	01
Neoteryx® microsampler (VAMS) 10µl (Torrance, CA, USA)	01
2ml bar-coded Cryotubes (Biosigma, Cona, VE, Italy)	02
Delta-T® cooler bag (10L) containing icepacks (Delta-T, Germany)	01
Rack	01
Cryolabels	01 sheet
4 mL EDTA tube (BD, Franklin Lakes, NJ, USA)	01
Biohazard needle container	01
Biohazard Bag	01
Samsung Tablet	01

PROCEDURE

1. Collection Procedure

- After delivery and expulsion of the placenta, wear gloves and place the placenta on a tray without removing the forceps placed by the staff who performed the delivery.
- Clean the excessive blood on the cord blood using 3 Phosphate-buffered saline (PBS) soaked sterile gauzes.
- Adapt an 18G needle to a 5cc or 10cc syringe.
- Prick an artery on the umbilical cord using the needle, then once in the artery draw blood from the cord. Draw at least 5 mL of blood with the syringe (Figure 1)

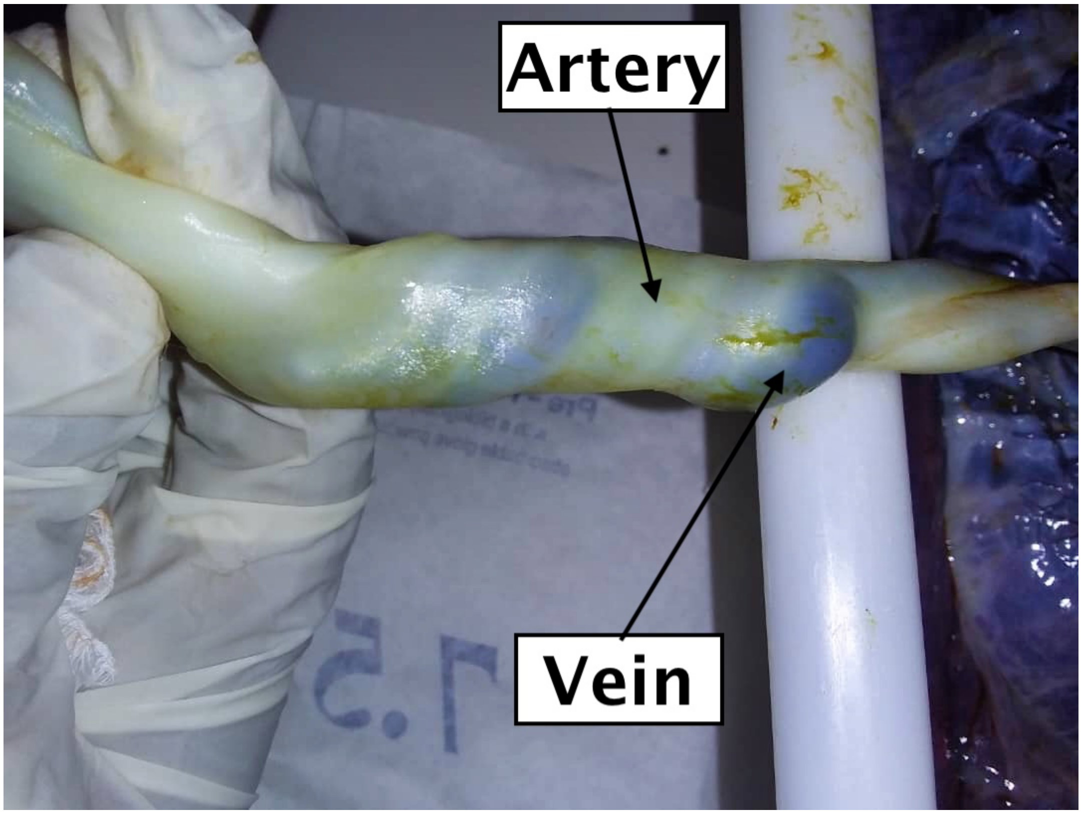

Figure 1: Location of the arteries in the cord

- From the 5ml collected:
  - Transfer 500µL of collected blood into the 500µL EDTA microtainer tube,
  - Then put 1 drop of blood on a clean surface (the outside of the VAMS kit) to collect the 10µL VAMS.
  - Finally transfer the remaining blood contained in the syringe into the 4mL tube.
  - CAUTION: The 4mL EDTA tube has a vacuum system that immediately aspirates the blood from the syringe, so it is not necessary to depress the plunger of the syringe to transfer the blood from the syringe to the 4mL EDTA tube.
  - Gently mix by tilting 180° the two EDTA tubes (500µl microtainer and 4ml tube) 10 times immediately after collection.

- Label the 500µL microtainer EDTA tube acco_saed_db###e.
- Place the 500µL microtainer EDTA tube on the rack.
- Open the 4mL EDTA tube.
- Label 2 x 2mL bar-coded cryotubes as follow:
  - Cryotube1: acco._saca_db###e
  - Cryotube2: acco._sate_db###e.
  - Using a graduated transfer pipette (1mL) transfer 2mL of blood from the 4mL EDTA tube into the 2 pre-labeled 2mL bar-coded cryotubes.
  - Ensure that the cryotubes are tightly closed and that there are no leaks.
- Place the two 2mL bar-coded Cryotubes on the same rack as the 500µL microtainer EDTA tube (BD, Franklin Lakes, NJ, USA) and store them in the Delta-T® Cooler Bag (10L).
- Store the VAMS in their zip lock bag and label the zip lock bag acco_sv1ab_db###e

– Fill in the CAPI (data collection sheet) corresponding to the visit (delivery)

– Dispose of all biomedical waste in the biohazard bag

2. Storage

– The two 2ml cryotubes should be stored within a maximum of 2 hours after the delivery in liquid nitrogen. Once the liquid nitrogen tank is filled with cord blood aliquot cryotubes, transfer the liquid nitrogen tank to the laboratory for further storage of samples in the -80°c freezer.

– The 500µL microtainer EDTA tube should be transferred within a maximum of 4 hours after collection to the immunology laboratory.

– The 10µL VAMS should be transferred to the laboratory within 3 days.

- For samples collected at night by the maternity ward staff. The 500µL microtainer EDTA tube and the 4ml EDTA tube are stored in the fridge at 2°c – 8°c. The 10µL VAMS are put in their zip lock bag and kept at room temperature. The following morning DENBALO's staff will aliquot, and store collected samples according to the first three steps outlined in the storage section of the procedure.

Late-term Pregnancy Ultrasound for Assessment of Gestational Age: Standard Operating Procedures (SOP) for the DenBalo Study

Trenton Dailey-Chwalibóg, M.P.H., Ph.D.,[1] Anne C.C. Lee, M.D., M.P.H.,[2] Blair J. Wylie, M.D.,[3] Cheick Ahmed Ouattara, M.D., M.P.H.,[4,5] Hermann Ouattara, M.D.,[6] Jan Aerts, M.D.,[7] Moussa Coulibaly, M.D.,[6] Moctar Ouédraogo, M.S.,[4] Lionel Olivier Ouédraogo, M.S., M.D.,[4,8] Laéticia Céline Toé, M.D., M.S.,[1,9] Oumarou Thombiano, M.D.,[6] Patrick Kolsteren, M.D., Ph.D.,[1] Marie Carteau, M.D.,[10] Zachari Nikiéma, M.D.[11]

Abstract

Purpose: These standard operating procedures provide sonographers with the general information on fetal biometric imaging for the "Description and Comparison of Biological Vulnerability in Small, vulnerable newborns versus Healthy community controls in Urban Burkina Faso (DenBalo) Study: Gut Microbiota, Immune System, and Breastmilk Assembly and Development in the First Days and Weeks of Life" (ONZ-2022-0500, 050-2022/CEIRES du 16 septembre 2022). Scope: All sonographers involved in performing fetal ultrasound imaging must familiarize themselves with the content of this SOP.

Keywords: Abdominal Circumference (AC), Biparietal diameter (BPD), Femur Length (FL), Transverse Cerebellar Diameter (TCD).

[1] Department of Food Technology, Safety and Health, Faculty of Bioscience Engineering, Ghent University, Ghent, Belgium
[2] Brigham and Women's Hospital, Harvard Medical School, Boston, MA, United States
[3] Massachusetts General Hospital, Division of Maternal-Fetal Medicine, Department of Obstetrics and Gynecology, Harvard Medical School, Boston, MA, United States
[4] Agence de Formation de Recherche et d'Expertise en Santé pour l'Afrique (AFRICSanté), Bobo-Dioulasso, Burkina Faso
[5] École Doctorale de Santé Publique, Université Nazi Boni, Bobo-Dioulasso, Burkina Faso
[6] Centre Médical avec Antenne Chirurgicale de Dô, Bobo-Dioulasso, Burkina Faso
[7] AZ Turnhout Campus Sint Jozef, Turnhout, Belgium
[8] Centre Muraz, Bobo-Dioulasso, Burkina Faso
[9] Unité de nutrition et maladies métaboliques, L'Institut de Recherche en Sciences de la Santé (IRSS), Bobo-Dioulasso, Burkina Faso
[10] Faculté de Médecine, Université Paris Cité, Paris, France
[11] Clinique Universelle du Houet, Bobo-Dioulasso, Burkina Faso

Notes

– Fetal biometry should be assessed in the following order:

1. Biparietal diameter
2. Head circumference
3. Transcellular diameter
4. Abdominal circumference
5. Femur length

– All images must be saved.

MATERIALS

MATERIALS	QUANTITIES
Ultrasound machine	01
Ultrasound transmission gel	01 bottle
Paper towel	01 roll
USB key	01
Laptop	01
Printer	01
A4 paper sheet	01
Pen	01

PROCEDURE

1. Biparietal Diameter (BPD)

- Biparietal Diameter (BPD) is the maximum diameter of a transverse section of the fetal skull at the level of the parietal eminences (cf. **Figure 1**).[1]

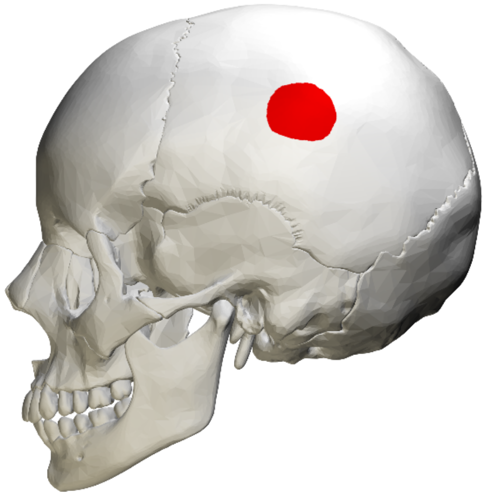

Figure 1. Lateral view of parietal eminence in the human skull

Source: BodyParts3D/Anatomography (CC BY-SA 2.1 JP)

- Identify the fetal head and orient the transducer such that the skull is imaged side-to-side on the screen.[2] Ensure that the skull is oval in shape and symmetrical.
- Find the appropriate plane by identifying the thalamus and the cavum septum pellucidum (CSP) as anatomical landmarks.

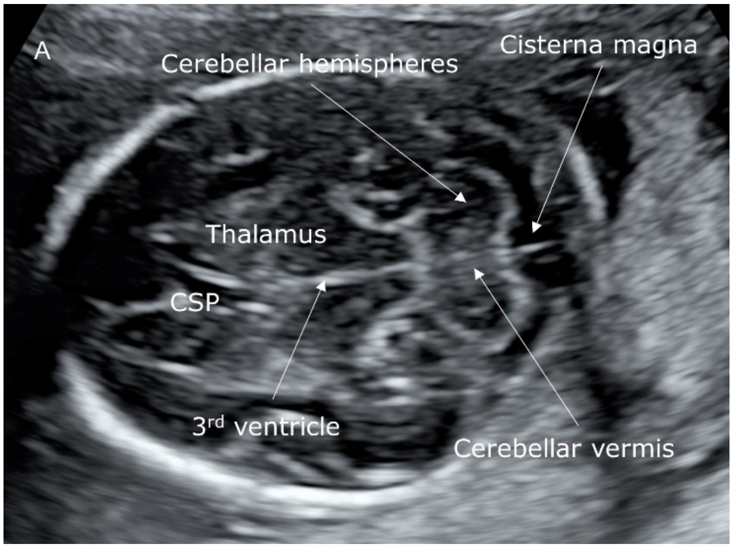

Figure 2. Transverse cerebellar cross-sectional ultrasound image of the fetal head at 21^{+}2 weeks of gestation showing the thalamus, cavum septum pellucidum, third ventricle, cerebellar hemispheres, and cerebellar vermis.

Source: Yang et al. 2021[3] (CC BY 4.0)

– Ensure appropriate magnification to make optimum use of the field of view.[4] The fetal image should ideally occupy more than 50%.[2]

– Place callipers at the widest part of the skull, perpendicular to the midline echo.[1] Place the top caliper on the outer edge of the prximal calvarial wall and the bottom caliper on the outer edge of the distal calvarial wall (i.e., outer edge to outer edge).[1,5]

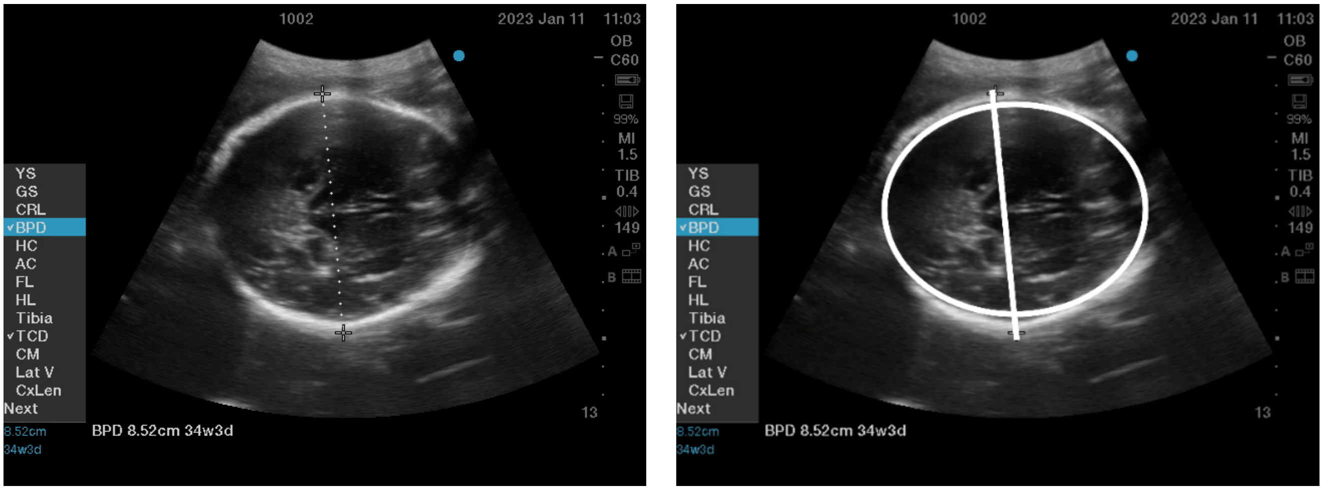

Figure 3. Representative sample image of biparietal diameter

– BPD quality control is assessed using the following criteria:

BPD (outer-outer)							
Zooming	Frozen in correct plane			Caliper placement			Total
Good magnification (30% of image size)	Skull is oval and bone visible throughout	Thalamus is visible	Skull side to side on screen	Calipers placed perpendicular to long axis of skull	Top caliper placed on outer portion of the skull	Bottom caliper placed on outer portion of the skull	Highest score = 7 per image

2. Head circumference (HC)

– Ensure good magnification of the image (with the fetal head occupying at least 30% of the image).

– Adjust the gain to obtain the sharpest image.

- Ensure that the skull is oval in shape and visible all the way around.
- Look for the butterfly (thalamus) and the equal sign (cavum septum pellucidum). Early in the second trimester, it may be difficult to visualize the cavum.
- The cerebellum should NOT be in the image. Place the ellipse around the OUTSIDE border of the skull, which is visible as the bright white line. Take care not to include the skin, which is a fainter line beyond the skull.
- The HC is measured by placing the elliptical cursors on the outer edge of the skull. See reference image below.

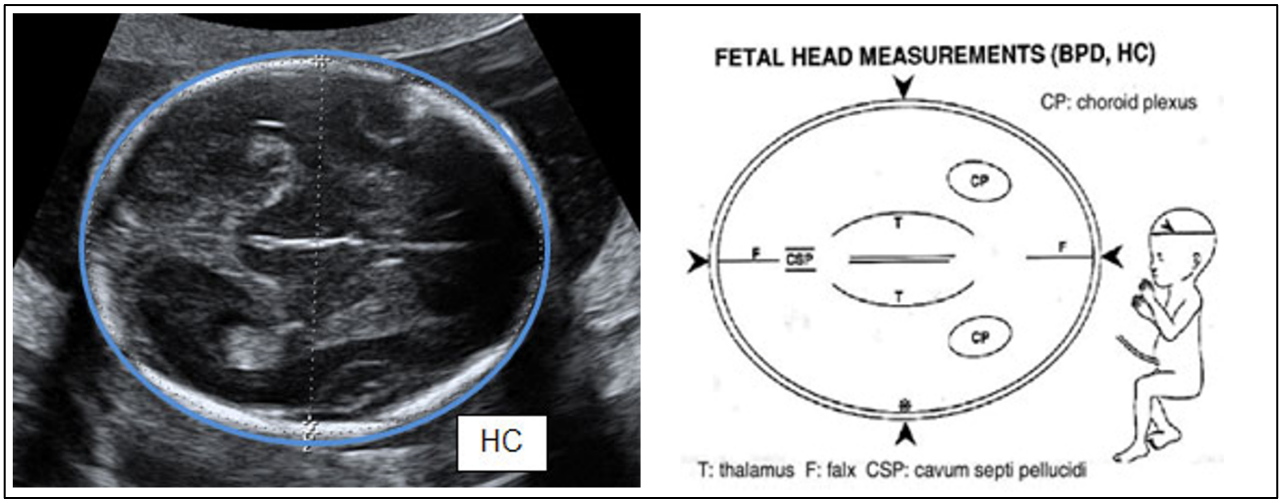

- HC quality control is assessed using the following criteria:

HC							
Zooming	Frozen in correct plane			Caliper placement			Total
Good magnification (30% of image size)	Skull is oval and bone visible throughout	Thalamus visible	Cavum septum pellucidum visible	Skull side to side on screen	Ellipse placed around the outer circumference	Skin is not included in the caliper placement	Highest score= 7 per image

3. Transcerebellular diameter (TCD)

- After obtaining an adequate image for the BPD, rotate the transducer inferiorly and to image the cerebellum. The cerebellum will appear as peanut-shaped figure (or figure of 8) in the back of the skull behind and a little lower than the thalamus. Later in the third trimester, the cerebellum appears more pyramidal in shape.
- Ensure good magnification of the image (with the fetal head occupying at least 50% of the image) and adjust the gain to obtain the sharpest image.
- You will likely see the midline structures (thalamus, cavum septum pellucidum) but at times in the third trimester to optimize imaging of the cerebellum these shapes may be shadowed out. The head need not be imaged side to side on the screen. Especially late in gestation, the posterior fossa may need to be closer to the transducer to avoid shadowing from the skull over the cerebellum.
- Ensure that the cerebellum is visible at the point of its greatest width. This requires scanning up and down the cerebellum to find the maximal width.
- The TCD is measured from the outer edge of the nearer cerebellar hemisphere to the outer edge of the more distant cerebellar hemisphere (outer-to-outer). The following key steps should be followed: The calipers should be placed across the greatest width of the cerebellum. This will be perpendicular to the long axis of the skull. The top caliper should be placed touching the outer aspect of the nearer cerebellar hemisphere, and the lower caliper touching the outer aspect of the farther cerebellar hemisphere. See reference image below.

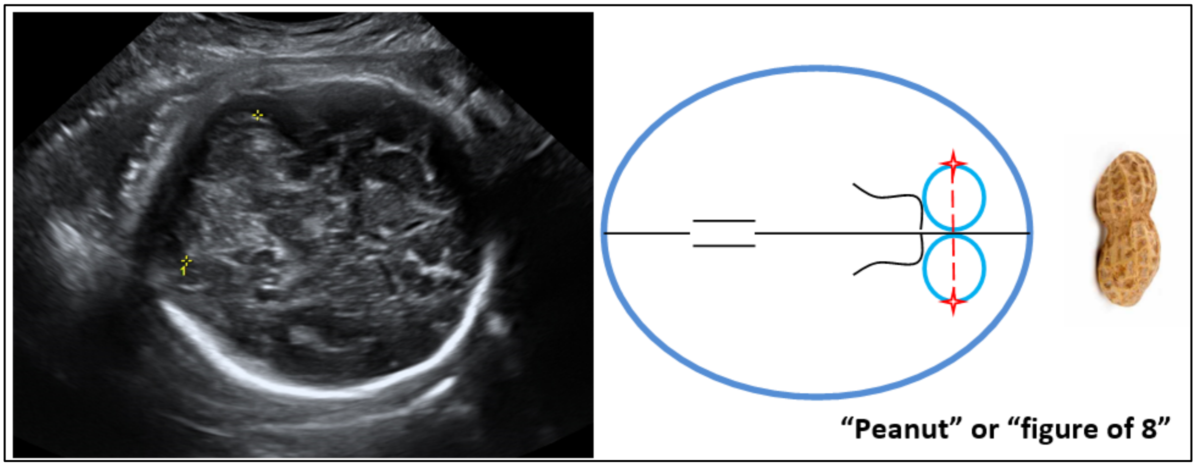

– TCD quality control is assessed using the following criteria:

TCD					
Zooming	Frozen in correct plane		Caliper placement		Total
Good magnification (30% of image size)	Cerebellum visible in image	Cerebellar hemispheres appear symmetric (upper and lower similar in size)	Top caliper placed on outer margin of upper cerebellar hemisphere	Lower caliper placed on outer margin of lower cerebellar hemisphere	Highest score = 5 per image

4. Abdominal circumference (AC)

– First, find the head then scan down the baby's spine until just below the level of the heart. The abdomen should fill at least 30% of the monitor.

– Make sure the belly is round not oval. The sonographer should avoid applying too much pressure with the transducer, which can distort the shape.

- The stomach should be visible as a black circle/ellipse. The umbilical vein (small black oval or rectangle) should be located in the anterior third of the abdomen. The kidneys and bladder should not be visible. The heart should also not be in the picture.
- The spine should be preferably positioned at the 3 or 9 o'clock position to avoid shadowing.
- The ribs should encircle about half of the abdomen and should be continuous rather than interrupted. This ensures that the plane is correct rather than angled.
- The AC is measured by placing the elliptical cursors on the outer edge of the abdominal wall. Place the round caliper to best approximate the circumference around the soft tissue of the abdomen (not at the level of the bright white ribs, but at the skin). This is different from the head circumference. See reference image below.

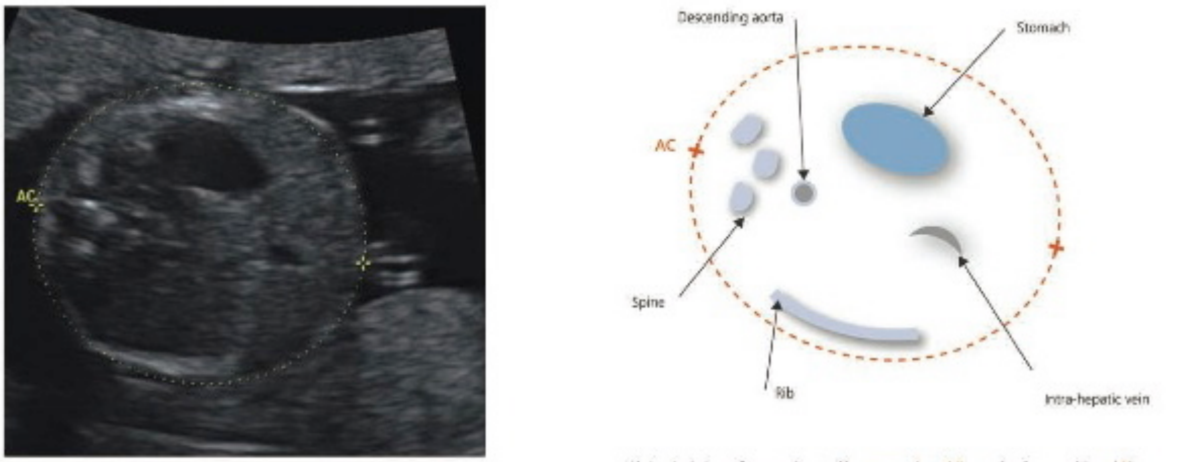

Abdominal circumference denoted by orange dotted line and caliper markings (+)

- AC quality control is assessed using the following criteria:

AC							
Zooming	Frozen in correct plane			Caliper placement			Total
Good magnification (30% of image size)	Abdomen appears round, not oval	Stomach and umbilical vein visible	Kidneys and heart not visible	Ribs are not interrupted but continuous	Vertebrae at 3 or 9 o'clock	Ellipse placed around skin line, not rib line	Highest score = 7 per image

5. Femur length (FL)

- Identify the femur and differentiate the femur from the humerus or the bones of the lower extremities (tibia and fibula). The femur is the single bone in the upper leg (thigh) and therefore only one bone should be visible. Rather than randomly looking for a bone to measure that might be the humerus, the sonographers will need to take care that they follow the thorax to the pelvis and identify the femur bone attached to the pelvis.
- Ensure you have good magnification and gain for the best quality images
    - The femur fills almost the entire screen (at least 30% magnification or more).
    - Adjust the gain to obtain the sharpest image from the fetus.
- Align the transducer along the long axis of the bone so that the beam is perpendicular to the shaft (bone is side to side on screen). This ensures that dropout will not occur.
- The femur length should only be measured when the femur is horizontal (beam is perpendicular) or at an angle less than 45° and shadows evenly - at least from both ends.

– Ensure that the measured ends are blunt and not pointed (see black arrows in figure below) to ensure that one is measuring the full extent of the femur bone, and not including the trochanter in the measurement.

– The length is measured from blunt end to blunt end parallel to the shaft.

– The calipers are placed along the diaphyseal shaft and at each outer end (outer to outer) end. The secondary ossification centers at the epiphysis should not be measured. See reference image below.

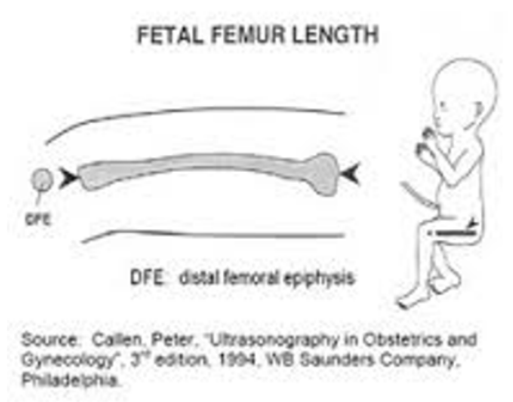

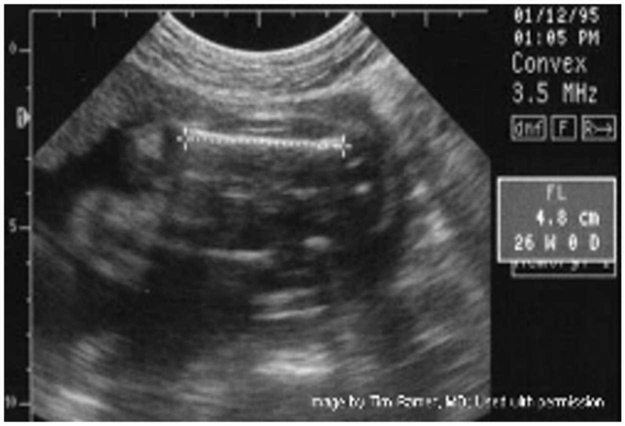

– FL quality control is assessed using the following criteria:

FL							
Zooming	Frozen in correct plane				Caliper placement		Total
Good magnification (30% of image size)	Femur imaged side-to-side on screen	Only one bone in this portion of extremity	Upper femur measured	Full extent of femur visualized (solid straight line)	Calipers placed at edge of echogenic bone (outer to outer)	Secondary ossification centers not measured	Highest score = 7 per image

Deuterium oxide (D2O) weighing and labeling : Standard Operating Procedures (SOP) for the DenBalo Study

Laéticia Céline Toé,[1,2] Lionel Olivier Ouédraogo,[1,3] Moctar Ouédraogo,[4] Cheick Ahmed Ouattara,[4,5] Anderson Compaoré,[4] Carl Lachat,[1] Trenton Dailey-Chwalibóg[1]

Abstract

Standard operating procedures are essential for ensuring the accurate and consistent administration of deuterium oxide (D2O) doses in studies of body composition. In this study, D2O doses were precisely weighed to the nearest 0.0001 g using a precision balance. The body composition of mothers was evaluated using the stable isotope dilution method with D2O. Saliva samples were then analyzed for D2O concentration via Fourier-transform infrared (FTIR) spectrophotometry, utilizing the Agilent 4500 Series device.

Keywords: Standard operating procedure, deuterium oxide, mothers, stable isotope dilution method, body composition.

---

[1] Department of Food Technology, Safety and Health, Faculty of Bioscience Engineering, Ghent University, Ghent, Belgium
[2] Unité Nutrition et Maladies Métaboliques, Institut de Recherche en Sciences de la Santé (IRSS)
[3] Centre Muraz, Bobo-Dioulasso, Burkina Faso
[4] Agence de Formation de Recherche et d'Expertise en Santé pour l'Afrique (AFRICSanté)
[5] École Doctorale de Santé Publique, Université Nazi Boni, Bobo-Dioulasso, Burkina Faso

NOTES

- Deuterium oxide should not be weighed or labeled in the laboratory due to the risk of contamination from non-consumable chemicals. This procedure can be safely performed in a clean kitchen environment.
- The DenBalo study biologist, who is required to wear nitrile gloves, is responsible for preparing the dose.
- For DenBalo study the dose is 30g or 27ml.

MATERIALS

MATERIALS	QUANTITIES
0.0001-gram precision electronic scale (model SAB 224i, Adams Equipment, Felde, Germany)	01
Graduated cylinder	01
Labels	01 sheet
Permanent marker or pen	01
60ml autoclavable polypropylene bottle with screw, leak-free cap	01
Nitrile gloves	01
Paper towel	01 roll
50ml falcon tube	01
Laboratory logbook	01

PROCEDURE

- Uncap deuterium bottle and pour into 50ml falcon tube. Immediately recap the deuterium bottle.
- Tare the empty scale

- Weigh the 60ml autoclavable polypropylene bottle with its lid and note the weight of the empty and closed bottle in a laboratory logbook.
- Remove the 60ml autoclavable polypropylene bottle from the balance and open it.
- Pour the deuterium into the 60ml autoclavable polypropylene bottle to one-third volume.
- Close the 60ml autoclavable polypropylene bottle, weigh and record the new weight (bottle + lid + deuterium).
- If the difference between the new weight (bottle + lid + deuterium) and the initial weight (bottle + lid) is 30g, label the 60ml autoclavable polypropylene bottle and store it in the refrigerator at 2 - 8°c.
- If the weight difference is less than 30g add deuterium using the 10ml oral syringe to add small volumes accurately.
- If the weight difference is greater than 30g, use the 10ml oral syringe to remove the excess volume.
- Following dose preparation, document the deuterium dosage details and record the storage information in the laboratory logbook.

Collection of saliva samples: Standard Operating Procedures (SOP) for DenBalo Study

Laéticia Céline Toé,[1],[2] Lionel Olivier Ouédraogo,[1],[3] Moctar Ouédraogo,[4] Cheick Ahmed Ouattara,[4],[5] Anderson Compaoré,[4] Carl Lachat,[1] Trenton Dailey-Chwalibóg[1]

Abstract

Standard operating procedures are crucial to guarantee sample collection consistency and quality during a study. The importance of high-quality biological samples is amplified by researchers' pursuit of a multi-omics approach. Many studies have been carried out in developed countries explaining the procedures used for biological samples collection for multi-omics analysis. However, a little or none of those studies were carried out in Africa where working conditions are totally different and sometimes rudimentary.

This standard operating procedure (SOP) provides a guideline for saliva samples collection in mothers enrolled in the study "Description and Comparison of Biological Vulnerability in Small, vulnerable newborns versus Healthy community controls in Urban Burkina Faso (DenBalo) Study: Gut Microbiota, Immune System, and Breastmilk Assembly and Development in the First Days and Weeks of Life" (ONZ-2022-0500, 050-2022/CEIRES du 16 September 2022). In this study the deuterium oxide (D2O) method was used to assess breast milk intake in newborns and to assess body composition in mothers.. The D2O concentration in the saliva will be analyzed using Fourier-transform infrared (FTIR) spectrophotometry, with the Agilent 4500 Series device.

Keywords: Standard operating procedure, deuterium oxide, mothers, stable isotope dilution method, body composition.

[1] Department of Food Technology, Safety and Health, Faculty of Bioscience Engineering, Ghent University, Ghent, Belgium
[2] Unité Nutrition et Maladies Métaboliques, Institut de Recherche en Sciences de la Santé (IRSS)
[3] Centre Muraz, Bobo-Dioulasso, Burkina Faso
[4] Agence de Formation de Recherche et d'Expertise en Santé pour l'Afrique (AFRICSanté)
[5] École Doctorale de Santé Publique, Université Nazi Boni, Bobo-Dioulasso, Burkina Faso

Notes

- DenBalo Study staff member should wear gloves during the procedure.
- This procedure is carried out after the weighing and labeling of deuterium oxide.
- The procedure is performed on both mothers and children.
- IMPORTANT!!! Bring all the bottles that contained the deuterium doses back to the lab to be weighed to calculate the precise amount of deuterium administered to each participant.

MATERIALS

MATERIALS	QUANTITIES
60ml autoclavable polypropylene bottle containing 27ml of deuterium	01
Gloves	02
2ml Bar-coded cryotube	02
Cotton ball	02
Drinking Straw	01
Small ziplock bags	02
Medium size ziplock bag	02
20ml Single-use syringe	01
10ml Single-use syringe	01
Biohazard bag	02
Delta-T® cooler bag (10L)	01

Procedure

1. Perform anthropometric measurements in mothers and children

- Open the corresponding CAPI
- Make sure that the open CAPI matches the identification number written on the written on the identification card placed in the mother and child health booklet
- Normally, the height of the mother and the child are already known. Take the weight of both and record it on the CAPI

2. Conditions for deuterium oxide administration

- Explain the test procedure and obtain the mother's approval
- The health worker should wear a new pair of gloves between saliva collections (different gloves for mother and child)
- All equipment used for saliva collection must be dry
- 60ml autoclavable polypropylene bottle and cryotubes are for single use only

3. Collecting pre-dose saliva

- Label 2 x 2ml cryotubes, 1 for the mother and 1 for the child. The label must include:
    - The identification number of the participant (child or mother)
    - The date
    - The mention "J001" indicating that it is the pre-dose saliva sample
- Ensure that the participant has not consumed any food or drink for at least 30 minutes prior to the saliva collection.
- For the mother: Ask the participant to chew a cotton ball for at least 2 minutes to ensure that it is well soaked

– For the child: Wrap a cotton ball around a straw and move it around inside the child's cheeks until it is thoroughly moistened (figure 1). Several repetitions may be necessary to obtain the necessary amount of saliva (about 1 ml).

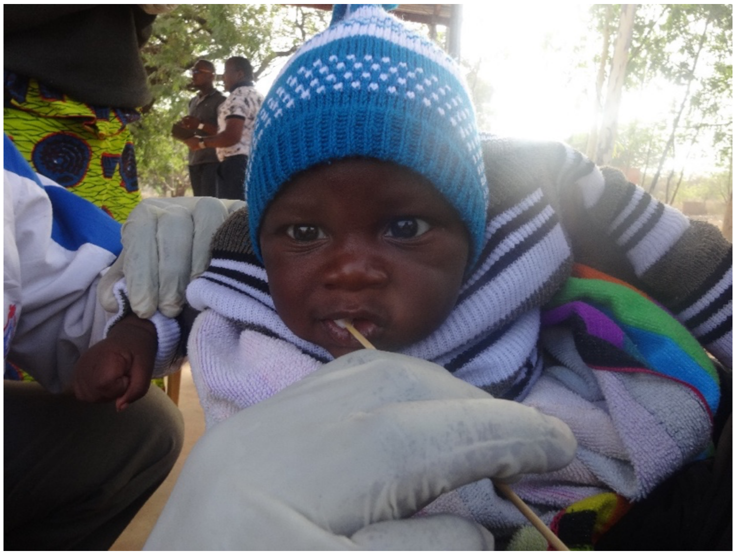

Figure 1: Procedure for Saliva Collection Using Cotton Ball and Straw

– Collect the cotton ball(s) in a single-use syringe of 20 ml for the mother and 10 ml for the child, after removing the plunger.

– Readjust the plunger of the syringe, take the appropriate cryotube and squeeze the saliva into the cryotube.

– Recap the cryotube and discard the syringes into your biohazard bag.

– Place the bags containing the mother's and child's saliva into 2 separates small ziplock bags and then place these 2 bags into a medium size bag.

– Label the medium-sized bag with:

  - The identification number of the participants (child and mother)
  - The date
  - J001 indicating that this is the sample before the deuterium dose is administered

- Store the samples in your Delta-T® cooler bag (10L)
- In the adapted questionnaire, enter the time of obtaining the saliva and the quantity of saliva obtained in ml.

4. Administering the deuterium dose to the mother

- Take the 60ml autoclavable polypropylene bottle containing the deuterium dose
- Note the number and weight of the 60ml autoclavable polypropylene bottle + cap + deuterium on the CAPI
- If the dose of deuterium oxide was stored in a cool place and is frozen, the dose vial must be allowed to thaw completely before use
- Using a straw, give the dose to the mother. The dose must be completely administered to the mother.
- Pour drinking water into the bottle, re-cap, shake and give the water to the mother. Repeat this a second time.
- Note the time of administration for the mother on the CAPI
- Store the empty deuterium bottles with their respective caps in a ziplock bag. Make sure that each bottle keeps its own cap. Do not interchange caps!

5. Collection of the first post-dose saliva sample ("saliva J001, J003, J004, J013, J014")

- Label 2 x 2ml cryotubes, 1 for the mother and 1 for the child. The label should include:
    - The identification number of the participant (child or mother)
    - The date
    - **J001, J003, J004, J013, J014** indicating the corresponding day
- Take the saliva samples as described above and store them in the cooler in 2 ziplock bags.

6. Preserve samples for transport and analysis

- Put the bags containing the mother's and child's saliva into 2 separates small ziplock bags and then put these 2 bags into a medium bag.
- Label the medium-sized bag with:
  - The identification number of the participants (child and mother)
  - The date
  - **J001, J003, J004, J013, J014** indicating the corresponding day
- Samples stored in a refrigerator at 4-8°C must be analyzed within 7 days
- Samples stored at -80°C can be kept for several years
- IMPORTANT!!! Bring all the bottles that contained the deuterium doses back to the lab to be weighed to calculate the precise amount of deuterium administered to each participant.

Collection of placenta samples: Standard Operating Procedure (SOP) for the DenBalo Study

Lionel Olivier Ouédraogo,[1,2] Trenton Dailey-Chwalibóg,[1] Lieselot Y. Hemeryck,[3] Lynn Vanhaecke,[3] Moctar Ouédraogo,[4] Anderson Compaoré,[4] Cheick Ahmed Ouattara,[4,5] Carl Lachat,[1] Laéticia Céline Toé,[1,6]

Abstract

Standard operating procedures are crucial to guarantee sample collection consistency and quality during a study. The quality of biological samples is all the more important when researchers envision a multi-omics approach. Many studies have been carried out in developed countries, explaining the procedures used for biological sample collection for multi-omics analysis. However, little or none of those studies were carried out in Africa where working conditions are totally different and sometimes rudimentary.

This standard operating procedure (SOP) provides a guideline for placenta sample collection in mothers enrolled in the study “Description and Comparison of Biological Vulnerability in Small, vulnerable newborns versus Healthy community controls in Urban Burkina Faso (DenBalo) Study: Gut Microbiota, Immune System, and Breastmilk Assembly and Development in the First Days and Weeks of Life” (ONZ-2022-0500, 050-2022/CEIRES du 16 September 2022). Placenta samples are dedicated for DNA adductomics analysis using the Orbitrap Exploris 120 mass spectrometer.

The aim of this SOP is to establish a standardized process for placenta sample collection, ensuring process consistency. To that purpose, this SOP details steps to follow to undertake a good sample collection, including material and tools preparation, specimen collection, labeling, handling and storage, and the list of required tracking documents to fill out during the process. By following this SOP, healthcare professionals can optimize the quality and reliability of placenta samples collected. Our SOP could be a reference guideline on placenta sample collection in Africa, specifically tailored for DNA adductomics analysis.

Keywords: Standard Operating Procedure (SOP), Placenta samples, DNA adductomics

[1] Department of Food Technology, Safety and Health, Faculty of Bioscience Engineering, Ghent University, Ghent, Belgium
[2] Centre Muraz, Bobo-Dioulasso, Burkina Faso
[3] Laboratory of Integrative Metabolomics, Faculty of Veterinary Medicine, Ghent University, Merelbeke, Belgium
[4] Agence de Formation de Recherche et d’Expertise en Santé pour l’Afrique (AFRICSanté)
[5] École Doctorale de Santé Publique, Université Nazi Boni, Bobo-Dioulasso, Burkina Faso
[6] Unité Nutrition et Maladies Métaboliques, Institut de Recherche en Sciences de la Santé (IRSS)

Notes

- Do not collect placental tissue beyond 30 minutes after delivery.
- The maximum time limit from collection to storage of 2 mL cryotubes in liquid nitrogen is 1 hour for DENBALO's staff.
- The maximum time limit from collection to storage of 2 mL cryotubes in the freezer (0 °C - 2 °C) is 30 minutes for the maternity ward staff.
- The labelling and the CAPI completion steps are not performed by the maternity ward staff. They immediately store the samples in the freezer after collection.

MATERIALS

MATERIALS	QUANTITIES
Pen	01
Soap	01
PBS solution	At least 100 mL
Sterile gauze	03
50 mL Falcon tube	01
Tray	01
Scalpel blade	01
Scalpel sleeve	01
Tweezers	04
2 mL bar-coded Cryotubes	02
Delta-T® cooler bag (10 L) containing ice	01
Rack	01
Cryolabels	01 sheet
Biohazard needle container	01
Biohazard Bag	01
Samsung Tablet	01

PROCEDURE

1. Collection of placenta samples

- Wear gloves
- Pour about 25 mL of PBS in the 50mL falcon tube. Recap tightly and save for further use.
- Place the placenta on the tray with the fetal side of the placenta facing you (Figure 1).

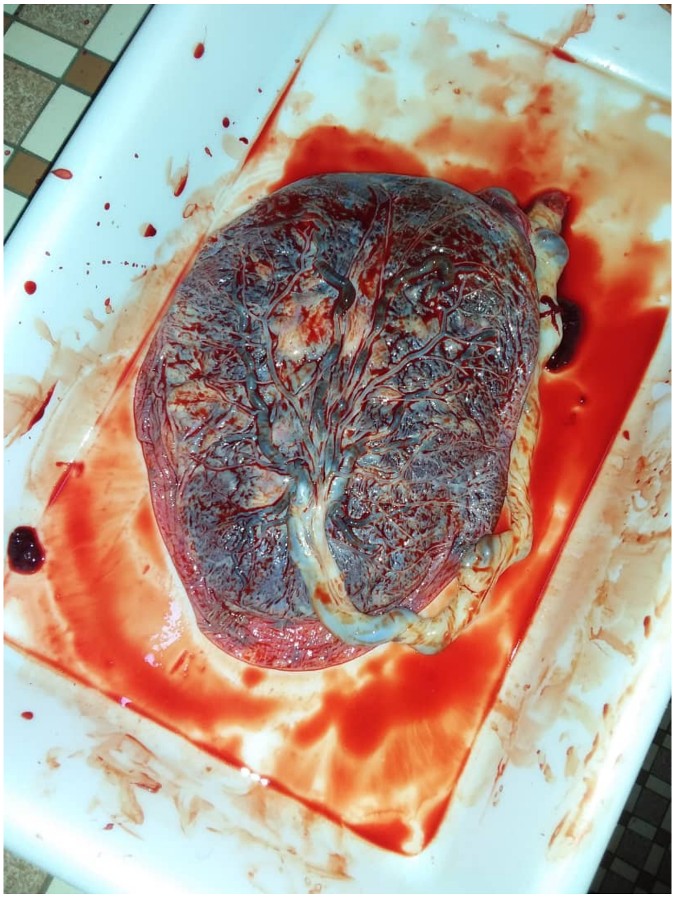

Figure 1: Fetal side of placenta

- Clean the excessive blood on the placenta using 3 PBS (Phosphate-buffered saline) soaked sterile gauzes.
- Identify an area approximately 2 cm from the umbilical cord between the large veins on the fetal side of the placenta for sampling.

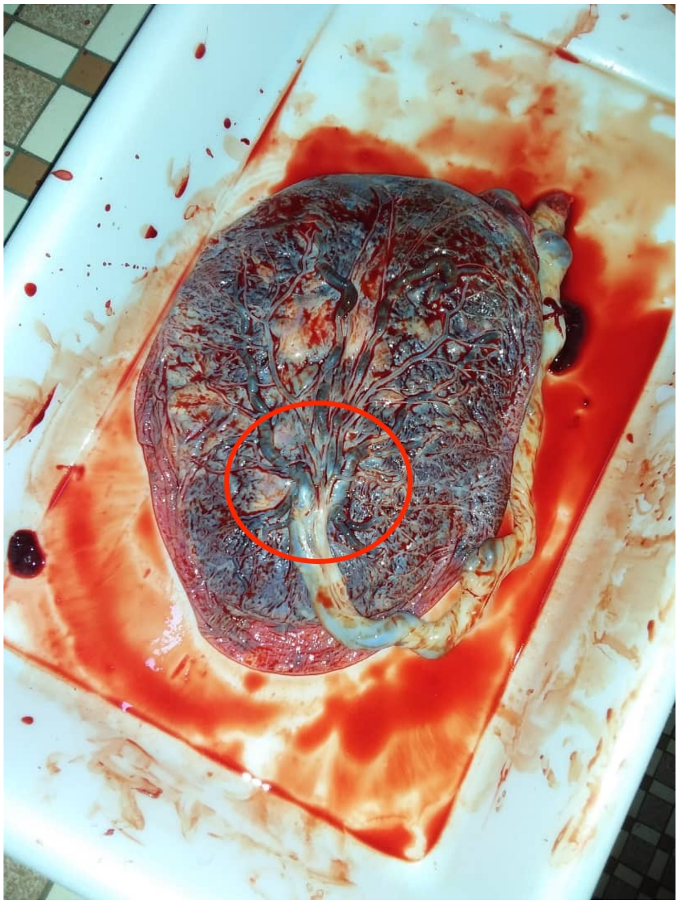

Figure 2: Collection area (red circle)

- Adapt a new scalpel blade on a disinfected scalpel sleeve.
- Using the scalpel, cut a 1.5 cm deep strip of placental tissue (enough to fill a 2mL tube).
- Collect 2 placenta tissue samples.
- Take each collected sample with the tweezers and dip in the 50 mL Falcon tube containing PBS. Shaking the tweezers gently to remove as much maternal blood as possible.
- Repeat the cleaning step in PBS three times to remove the maternal blood.
- Place the collected and cleaned tissue samples in 2 x 2 mL cryotubes.
- Label the cryotubes as follows:
    - 1st tissue sample: acco_pla1_db###e1

- 2nd tissue sample: acco_pla2_db###e1

- Place the cryotubes in the Delta-T® cooler bag immediately after collection.
- Fill in the CAPI corresponding to the visit (delivery).

2. Sample transport and storage

- Cryotubes should not remain in the Delta-T® cooler bag for more than 25 minutes and should be transferred as soon as possible into the liquid nitrogen tank at the health center.
- Once the liquid nitrogen tank is full, send it to the laboratory for transfer to the -80 °C freezer.
- For the maternity ward staff: immediately put the 2 placenta tissue samples in the freezer (0 °C – 2 °C) after collection and labelling. DENBALO’s staff will store the collected samples in the liquid nitrogen the following morning.
